# Modelling the Impact of Phenotypic Heterogeneity on Cell Migration: A Continuum Framework Derived from Individual-Based Principles

**DOI:** 10.1007/s11538-025-01502-5

**Published:** 2025-08-08

**Authors:** Rebecca M. Crossley, Philip K. Maini, Ruth E. Baker

**Affiliations:** Mathematical Institute, University of Oxford, Woodstock Road, Oxford, OX2 6GG United Kingdom

**Keywords:** Phenotypic structuring, Collective cell migration, Coarse-graining, T cells, Go-or-grow, Range expansion

## Abstract

Collective cell migration plays a crucial role in numerous biological processes, including tumour growth, wound healing, and the immune response. Often, the migrating population consists of cells with various different phenotypes. This study derives a general mathematical framework for modelling cell migration in the local environment, which is coarse-grained from an underlying individual-based model that captures the dynamics of cell migration that are influenced by the phenotype of the cell, such as random movement, proliferation, phenotypic transitions, and interactions with the local environment. The resulting, flexible, and general model provides a continuum, macroscopic description of cell invasion, which represents the phenotype of the cell as a continuous variable and is much more amenable to simulation and analysis than its individual-based counterpart when considering a large number of phenotypes. We showcase the utility of the generalised framework in three biological scenarios: range expansion; cell invasion into the extracellular matrix; and T cell exhaustion. The results highlight how phenotypic structuring impacts the spatial and temporal dynamics of cell populations, demonstrating that different environmental pressures and phenotypic transition mechanisms significantly influence migration patterns, a phenomenon that would be computationally very expensive to explore using an individual-based model alone. This framework provides a versatile and robust tool for understanding the role of phenotypic heterogeneity in collective cell migration, with potential applications in optimising therapeutic strategies for diseases involving cell migration.

**Supplementary Information:**

The online version contains supplementary material available at 10.1007/s11538-025-01502-5.

## Introduction

Mathematical models are essential tools for helping us to understand the key features and mechanisms underpinning biological processes, such as collective cell migration. Various modelling techniques exist to analyse cell behaviour during migration, ranging from microscopic, individual cell-based models to macroscopic-level, continuum-based models.

Stochastic individual-based models track the dynamics of single cells, describing migration through rules that dictate cell interactions with one another and their environment (Anderson and Rejniak 2007; Van Liedekerke et al. 2015; Wang et al. 2015; Cornell et al. 2019; West et al. 2023). Deterministic continuum models, on the other hand, usually focus on the collective migration of cells into external tissue, stroma, or the local environment, containing, for example, chemo-attractants, adhesive substances or other cell populations that impact cell migration (Trepat et al. 2012; Merino-Casallo et al. 2022). They utilise a variety of different mathematical approaches, such as partial differential equations (PDEs), that describe the evolution of cell densities and are amenable to both computational and analytical exploration. While some PDE models are adaptations of classical invasion models from other contexts, many are derived from first principles as the deterministic, continuum limit of stochastic, discrete models, such as individual-based models. This process of formally deriving the deterministic model ensures that the continuum equation provides a mean-field representation of the underlying dynamics of the individual cells and their environment, which is valid in specific parameter regimes (Macfarlane et al. 2022).

Mathematical models for cell invasion often assume that the cell population is phenotypically homogeneous, meaning every cell behaves identically in terms of division, movement and interactions with the local environment. However, such homogeneity is rarely present in biological systems. During collective cell migration, it is common to observe different cell types working together to facilitate invasion. The observable differences in physical or biochemical characteristics present within most cell populations are known as phenotypic heterogeneity. Over recent years, the role of phenotypic heterogeneity in cell populations has garnered significant attention. Models have been developed to account for distinctly different cell behaviours, using a discrete number of phenotypes (Chauviere et al. 2010; Stepien et al. 2018; Crossley et al. 2024b; Falcó et al. 2024; Carrillo et al. 2024), as well as for a continuous spectrum of phenotypes, whereby population members exhibit a variety of behaviours to different degrees (Bouin et al. 2012; Macfarlane et al. 2022). Variability in cell phenotypes can be incorporated into mathematical models of cell dynamics involving differential equations by introducing a variable to describe the phenotypic state of the cells.

When there is a discrete set of known cell phenotypes with distinct behaviors, it may be most appropriate to model the phenotypic state as a discrete variable, typically using integer values. However, when there are numerous (or potentially infinite) phenotypes with incremental differences or smoothly transitioning behaviours between them, then a continuously structured phenotype model might be more pertinent. In this case, the resulting evolution equation for the cell population density often takes the form of a non-local reaction-diffusion equation (Arnold et al. 2012; Berestycki et al. 2015; Lorenzi and Painter 2022), or a non-local advection-reaction-diffusion equation (Celora et al. 2021; Lorenzi et al. 2022; Celora et al. 2023). Understanding the role of different cell phenotypes during collective migration can guide experimental design, enhance understanding of cell behaviors, and aid the development of treatments for diseases where collective cell migration is crucial, such as during wound healing. However, a critical question arises when these continuum models are constructed phenomenologically, without derivation from an underlying individual-based model. In such cases, we may not fully understand the biological significance of the terms within the model, especially in realising their connection to the behaviours of the individual cells and their interactions with the local environment, leaving the meaning of various terms in the model ambiguous. Moreover, we may unknowingly be making intrinsic assumptions about cell behaviour that could be invalid. This research, therefore, focuses on constructing a general continuum model that is explicitly derived from the individual-based behaviours of the cells and their interactions with surrounding environmental features. This approach ensures that each term in the resulting continuum model has a well-defined interpretation in relation to the underlying cell dynamics, providing clarity and a deeper understanding of the biological processes being modelled. We are not, however, concerned with a detailed quantitative comparison between individual- and population-level models as this is already well explored in the literature (Ardaševa et al. 2020; Byrne and Drasdo 2009; Lorenzi et al. 2020; Lorenzi and Painter 2022; Macfarlane et al. 2022; Murray et al. 2009, 2011; Schaller and Meyer-Hermann 2006).

Therefore, in this article, we present a methodology for deriving a continuously structured PDE model for general cell migration into the local environment that is robustly derived from an underlying individual-based model that takes into account the individual interactions between the cells and their local environment. Using this approach, we demonstrate the model’s applicability to various biological scenarios, highlighting the flexibility of this general framework and the consistent, coherent connections between the microscale behaviours captured in the individual-based model and the macroscale descriptions in the resulting PDE model. For the purposes of the derivation of the continuum model, we have chosen cell invasion into the local environment in general, but due to its generality, the local environment could be replaced with a variety of substances, such as neighbouring tissues or organs, a tissue engineering scaffold or a wound, during healing. The applications in this article are carefully selected to illustrate a wide range of cell behaviours by employing different functional forms that describe the probabilities of movement in both physical and phenotypic spaces, as well as the behaviours governing growth at the individual-based level. However these applications were not chosen with the aim to provide detailed biological insights at this stage.

In Sec. 3.2, we consider a simplified model, without phenotype-driven migration, that shows how different growth mechanisms in the population impact the cell phenotypes present throughout a population over time. Next, in Sec. 3.3, we study the migration-proliferation dichotomy (that states that cells can either migrate or proliferate but cannot do both at the same time) for cells migrating into the extracellular matrix (ECM), by extending this model to consider a continuum of phenotypes with a trade-off between the cells ability to grow and divide and their ability to move and degrade ECM. In this model, we consider a range of different environmental features, such as the density of ECM, which we postulate could impact the phenotypic drift of the cells, and compare the resulting phenotypic structure of the invading cell population. Finally, in Sec. 3.4, we examine the results of the general macroscopic framework for depicting the phenotypic and spatial dynamics of T cells infiltrating into a tumour, as described by microscopic individual-based interactions. Here we consider the phenotype of the T cells to describe their exhaustion levels. Then, to summarise, future research directions and concluding remarks are discussed in Sec. 4.

## The Individual-Based Model

In order to incorporate microscopic descriptions of the interactions occurring between cells and their local environment, we formulate a phenotype-structured, on-lattice, individual-based model for collective cell migration.

In this model, the cells are represented as individual, discrete agents and we assume that the features of the local environment we are interested in, such as the ECM or other cell types, occupy some finite volume in a limited space. In order to fit in with the individual-based framework, we therefore choose to model the local environmental as being composed of individual, discrete elements of the same finite volume as the cells. Depending on the phenotype of the individual cell and the number of cells and elements of the local environment in the same lattice site, each individual cell has a capacity to undergo random, undirected movement, heritable phenotypic changes and proliferation, that can be adapted to the specific biological application of interest by employing appropriate individual-based rules to describe these changes. Furthermore, we assume that each individual cell can also interact with the surrounding environment, and that the cell’s capacity to impact their local environment depends on the phenotype of the cell.

Considering a one-dimensional spatial domain, we allow the cells and the local environment to be distributed in the region $x\in [X_{\text {min}}, X_{\text {max}}].$ We describe the phenotypic state of each individual cell through a structuring variable $y\in [Y_{\text {min}}, Y_{\text {max}}].$

In this individual-based model, we discretise the time variable $t\in \mathbb {R}^{+}$, as $t_h=h\Delta _t$ with $h\in \mathbb {N}$ and $\Delta _t\in \mathbb {R}^{+}.$ We discretise the spatial variable into an integer number of lattice sites $x_i = X_{\text {min}} + \Delta _x(i-1)$ for $\Delta _x\in \mathbb {R}^{+}$ and $i=1,\dots , N_x+1.$ We discretise the phenotype variable using $y_j = Y_{\text {min}}+ \Delta _y(j-1)$ for $\Delta _y\in \mathbb {R}^{+}$ and $j=1,\dots , N_y+1.$ In this case, $\Delta _t, \Delta _x, \Delta _y\in \mathbb {R}^{+}$ are the time-, space- and phenotype-step, respectively.

We introduce the dependent variable $n_i^j(t_h)\in \mathbb {N}_0$ to model the number of cells that occupy a position on the lattice ${x_i} \times {y_j}\in [X_{\text {min}}, X_{\text {max}}]\times [Y_{\text {min}}, Y_{\text {max}}]$ at time $t_h,$ where $\mathbb {N}_0$ represents the natural numbers, including zero. Then, we define the total cell number at a spatial position $x_i$ at time $t_h$ as $N_i(t_h)= \sum _{j=1}^{N_y+1}n_i^j(t_h)\in \mathbb {N}_0$ and the number of elements of the local environment at spatial position $x_i$ at time $t_h$ is denoted by $e_i(t_h)\in \mathbb {N}_0.$

### Modelling the Dynamics of the Cells

We denote by $p({\textbf{n}}, {\textbf{e}}, t_h)$ the joint probability that the number of cells in spatial position $x_i$ in phenotypic state $y_j$ (for $i=1,\dots , N_x+1$ and $j=1,\dots , N_y+1$) at time $t_h$ ($h\in \mathbb {R}$) is given by ${\textbf{n}}=([n_1^1, \dots , n_{N_x+1}^1], \dots , [n_1^{N_y+1}, \dots , n_{N_x+1}^{N_y+1}])$ and that the number of discrete, constitutive elements of the local environment in spatial position $x_i$ for $i=1,\dots , N_x+1$ is given by ${\textbf{e}}=[e_1, \dots , e_i, \dots , e_{N_x+1}].$

Between a time step $t_h$ and $t_{h+1}$ (equivalently described by ${{t_h}}+\Delta _t$), each cell in phenotypic state $y_j\in [Y_{\text {min}}, Y_{\text {max}}]$ at position $x_i\in [X_{\text {min}}, X_{\text {max}}]$ can undergo random movement, heritable phenotypic changes and cell proliferation independently of time and according to the following assumptions in this section. We note here that we write $n_i^j(t_h)$ as $n_i^j$ and $e_i(t_h)$ as $e_i$ for simplicity going forward.

#### Random Cell Movement

We model cell movement in space as an on-lattice, biased random walk between neighbouring lattice sites. The probability of cell movement can depend on a number of factors, such as the local environment or the phenotype of the cell, but it is easy to relax these assumptions to consider other variables of interest. In particular, we introduce the following two changes in state vector that describe movement left or right in physical space of a single cell in phenotypic state $y_j$ into position $x_{i\pm 1}$ from position $x_i$:

$$\begin{aligned} L_{i,j}^{\text {m}}&: [n_1^j, \dots , n_{i-1}^j, n_{i}^{j}, \dots , n_{N_x+1}^j] \longrightarrow [n_1^j, \dots , n_{i-1}^j+1, n_{i}^{j}-1, \dots , n_{N_x+1}^j], \\&\quad \text {for} \quad i=2, \dots , N_x+1, \quad j=1,\dots , N_y+1,\\ R_{i,j}^{\text {m}}&: [n_1^j, \dots , n_{i}^j, n_{i+1}^{j}, \dots , n_{N_x+1}^j] \longrightarrow [n_1^j, \dots , n_{i}^j-1, n_{i+1}^{j}+1, \dots , n_{N_x+1}^j], \\&\quad \text {for} \quad i=1, \dots , N_x, \quad j=1,\dots , N_y+1, \end{aligned}$$

where $L_{i, j}^{\text {m}}, R_{i,j} ^{\text {m}}: \mathbb {N}^{N_x+1}\rightarrow \mathbb {N}^{N_x+1}.$ We assume that the probability of cell movement depends on the phenotype of the cell and on the number of cells and elements of the local environment in the target site, rather than the lattice site that they are currently in. As such, we define the probability of movement to the left, to spatial position $x_{i-1}$ from $x_i$, during a single time step $\Delta _t$, as

$$\begin{aligned} \beta _{-}(j, N_{i-1},e_{i-1})\in [0,1], \qquad i=2, \dots , N_x+1, \quad j=1,\dots , N_y+1, \end{aligned}$$

and the probability of movement to the right, to spatial position $x_{i+1}$ from $x_i$, during a single time step $\Delta _t$, as described by the change to the state vector $R_{i,j}^{\text {m}}$, as

$$\begin{aligned} \beta _{+}(j, N_{i+1},e_{i+1})\in [0,1], \qquad i=1, \dots , N_x, \quad j=1,\dots , N_y+1, \end{aligned}$$

which depends on the phenotypic state of the cell, *j*, the number of elements of the local environment and the total number of cells in the target site. In order to ensure that cells cannot move to a physical site “outside of the domain" $x\in [X_{\text {min}}, X_{\text {max}}]$, we assume that cells cannot move left out of site $i=1$, or right out of site $i=N_x+1$, such that $\beta _{-}(j, N_{0}, e_{0})=0$ and $\beta _{+}(j, N_{N_x+2}, e_{N_x+2})=0.$ For $i=1, \dots , N_x+1, \; j=1,\dots , N_y+1$, cells remain in their current site (*i.e.*, do not move) with probability

$$\begin{aligned} 1-\beta _{+}(j,N_{i+1},e_{i+1})-\beta _{-}(j, N_{i-1},e_{i-1})\in [0,1]. \end{aligned}$$

#### Cell Proliferation

In order to model cell proliferation, we assume that a dividing cell is instantaneously replaced by two identical cells of equal volume to one another and the parent cell, such that the daughter cells inherit the spatial position and phenotypic state of the parent cell. As such, the corresponding change in state vector during a time step $\Delta _t$ can be written as

$$\begin{aligned} G_{i, j}&: [n_1^j, \dots , n_{i}^j, \dots , n_{N_x+1}^j] \longrightarrow [n_1^j, \dots , n_{i}^j -1, \dots , n_{N_x+1}^j],\\&\quad \text {for} \quad i=1, \dots , N_x+1, \quad j=1,\dots , N_y+1. \end{aligned}$$

To represent phenotype-dependent cell proliferation, we assume that the probability of a proliferation event is dependent on the phenotypic state of the cell, and the total number of cells and elements of the local environment in the same physical site as the cell that is dividing. Therefore, we define the probability that a cell in site *i* with phenotype *j* proliferates during time step $\Delta _t$, as

$$\begin{aligned} \gamma (j, N_i,e_i)\in [0,1], \qquad \qquad i=1, \dots , N_x+1, \quad j=1,\dots , N_y+1. \end{aligned}$$

The probability of a cell not undergoing proliferation during a time step $\Delta _t$ can then be written as

$$\begin{aligned} 1-\gamma (j, N_i,e_i)\in [0,1], \qquad \qquad i=1, \dots , N_x+1, \quad j=1,\dots , N_y+1. \end{aligned}$$

#### Cell Phenotypic Changes

During a single time step, $\Delta _t$, we model transitions in phenotype space from state $y_j$ to $y_{j\pm 1}$ via the following changes in state vectors:

$$\begin{aligned} D_{i,j}^{\text {p}}&: [n_i^1, \dots , n_{i}^{j-1}, n_{i}^{j}, \dots , n_{i}^{N_y+1}] \longrightarrow [n_i^1, \dots , n_{i}^{j-1}+1, n_{i}^{j}-1, \dots , n_{i}^{N_y+1}], \\&\quad \text {for} \quad i=1, \dots , N_x+1,\quad j=1, \dots , N_y+1, \\ U_{i,j}^{\text {p}}&: [n_i^1, \dots , n_{i}^{j}, n_{i}^{j+1}, \dots , n_{i}^{N_y+1}] \longrightarrow [n_i^1, \dots , n_{i}^{j}-1, n_{i}^{j+1}+1, \dots , n_{i}^{N_y+1}], \\&\quad \text {for} \quad i=1, \dots , N_x+1,\quad j=1, \dots , N_y+1, \end{aligned}$$

where $D_{i,j}^{\text {p}}, U_{i,j}^{\text {p}}: \mathbb {N}^{N_y+1}\rightarrow \mathbb {N}^{N_y+1}.$ A cell in site *i* transitions from phenotypic state $y_j$ to $y_{j+1}$ during time step $\Delta _t$ with a probability that depends on the phenotypic state of the cell and the total number of cells and elements of the local environment in the site *i*. Therefore, we can write that this transition, described by the change in state vector $U_{i,j}^{\text {p}}$, occurs with a probability

$$\begin{aligned} \mu _{+}(j, N_i, e_i)\in [0,1], \qquad i=1, \dots , N_x+1, \quad j=1, \dots , N_y+1. \end{aligned}$$

Similarly, a cell in site *i* transitions from phenotypic state $y_j$ to $y_{j-1}$ during time step $\Delta _t$ with a probability that depends on the phenotypic state of the cell and the total number of cells and elements of the local environment in the site *i*. Therefore, we can write that this transition, described by the change in state vector $D_{i,j}^{\text {p}}$, occurs with a probability

$$\begin{aligned} \mu _{-}(j, N_i, e_i)\in [0,1], \qquad i=1, \dots , N_x+1, \quad j=1, \dots , N_y+1. \end{aligned}$$

In order to ensure cells cannot transition to phenotypic states “outside of the domain" $y_j\in [Y_{\text {min}}, Y_{\text {max}}]$, we implement $\mu _{-}(1, N_i,e_i) = 0$ and $\mu _{+}(N_y+1, N_i, e_i) = 0.$ Taking this into consideration, the probability that a cell in phenotypic state $y_j$ and spatial position $x_i$ will not change phenotype during a time step $\Delta _t$ is given by

$$\begin{aligned} 1- \mu _{+}(j, N_i, e_i) - \mu _{-}(j, N_i, e_i)\in [0,1], \qquad i=1, \dots , N_x+1, \quad j=1, \dots , N_y+1. \end{aligned}$$

### Modelling the Dynamics of the Local Environment

We model degradation of elements of the local environment through contact with cells in the same physical site. Other cell-environment interactions, such as haptotaxis, or environmental changes such as production by cells, could also be considered here. The same methodology as presented in these sections can be followed to determine the corresponding population-level PDE. In particular, we define the change in state vector $H_i: \mathbb {N}^{N_x+1}\rightarrow \mathbb {N}^{N_x+1}$ to describe degradation of an element of the local environment in spatial position $x_i$ as:

$$\begin{aligned} H_i:[e_1, \dots , e_i, \dots , e_{N_x+1}] \longrightarrow [e_1, \dots , e_i +1, \dots , e_{N_x+1}], \qquad i=1, \dots , N_x+1. \end{aligned}$$

We assume that the probability of degradation of an element of the local environment during time step $\Delta _t$ depends on the number of cells in each phenotypic state *j* in the same spatial position $x_i$. As such, we define the probability of a cell in site *i* of phenotype *j* degrading an element of the local environment during a time step $\Delta _t$ as

$$\begin{aligned} \lambda (j, n_i^j)\in [0,1], \qquad i=1, \dots , N_x+1, \quad j=1, \dots , N_y+1,. \end{aligned}$$

### The Corresponding Continuum Model

In order to derive the corresponding continuum model describing the dynamics of the entire population of cells and the local environment over time, we employ a process known as coarse-graining. This procedure is described in full in the Supplementary Information. Assuming that the probability of two or more events occurring in time step $\Delta _t$ is sufficiently small that it can be ignored, the master equation, which describes the evolution of the probability density over time, is given by

1 $$\begin{aligned}&\Delta _t \dfrac{\partial }{\partial t} p({\textbf{n}}, {\textbf{e}}, t_h) + O(\Delta _t^2)\nonumber \\&\quad = \sum _{i=1}^{N_x+1}\sum _{j=1}^{N_y+1}\mu _{-}(j+1, N_i, e_i)\left\{ (n_i^{j+1}+1)p(U_{i,j}^{\text {p}}{\textbf{n}}, {\textbf{e}}, t_h)-n_i^{j+1}p({\textbf{n}}, {\textbf{e}}, t_h)\right\} \nonumber \\&\qquad + \sum _{i=1}^{N_x+1}\sum _{j=1}^{N_y+1}\mu _{+}(j-1, N_i, e_i)\left\{ (n_i^{j-1}+1)p(D_{i,j}^{\text {p}}{\textbf{n}}, {\textbf{e}}, t_h)-n_i^{j-1}p({\textbf{n}}, {\textbf{e}}, t_h)\right\} \nonumber \\&\qquad + \sum _{i=1}^{N_x} \sum _{j=1}^{N_y+1}\beta _{-}(j, {N_i,e_i)}\left\{ (n_{i+1}^{j}+1)p(R_{i,j}^{\text {m}}{\textbf{n}}, {\textbf{e}}, t_h)-n_{i+1}^{j}p({\textbf{n}}, {\textbf{e}}, t_h)\right\} \nonumber \\&\qquad + \sum _{i=2}^{N_x+1} \sum _{j=1}^{N_y+1}\beta _{+}(j, N_i,e_i)\left\{ (n_{i-1}^{j}+1)p(L_{i,j}^{\text {m}}{\textbf{n}}, {\textbf{e}}, t_h)-n_{i-1}^{j}p({\textbf{n}}, {\textbf{e}}, t_h)\right\} \nonumber \\&\qquad + \sum _{i=1}^{N_x+1} \sum _{j=1}^{N_y+1}\left\{ \gamma (j, N_i-1,e_i)(n_i^j-1)p(G_{i,j}{\textbf{n}}, {\textbf{e}}, t_h)-\gamma (j, N_i,e_i)n_i^jp({\textbf{n}}, {\textbf{e}}, t_h)\right\} \nonumber \\&\qquad + \sum _{i=1}^{N_x+1}\sum _{j=1}^{N_y+1} \lambda (j, n_i^j)\left\{ (e_i+1) p({\textbf{n}}, H_i{\textbf{e}}, t_h) - e_ip({\textbf{n}}, {\textbf{e}}, t_h)\right\} . \end{aligned}$$

Briefly, the first two lines on the right hand side correspond to changes in the phenotypic state of the cell, the second two correspond to changes in the physical position of the cell, the penultimate line describes proliferation of the cell and the final line describes degradation of the local environment.

#### The Coarse-Grained Model of the Cells

As is standard in the literature, we define the ensemble average for the function, *f*, of the number of cells at position $i=1,\dots , N_x+1$ in state $j=1,\dots , N_y+1$ and number of elements of local environment in lattice site $i=1,\dots , N_x+1$ in the following way:

2 $$\begin{aligned} {{\langle f(n_i^j, e_i)\rangle = \sum _{\textbf{n}}\sum _{\textbf{e}}f(n_i^j, e_i)p({\textbf{n}}, {\textbf{e}}, t_h).}} \end{aligned}$$

We can therefore formally derive (as seen in Supplementary Information Sec. S1) the following equation describing the evolution of the mean number of cells in physical site $i=1,\dots , N_x+1$ and phenotypic state $j=1,\dots , N_y+1$ based on the rules described in Sec. 2.1:

3 $$\begin{aligned} \dfrac{\partial }{\partial t} \langle n_i^j\rangle&= \dfrac{1}{\Delta _t}\langle \beta _{+}(j, N_i,e_i)n_{i-1}^j\rangle +\dfrac{1}{\Delta _t} \langle \beta _{-}(j, N_i,e_i)n_{i+1}^j\rangle \nonumber \\&\quad -\dfrac{1}{\Delta _t} \langle \beta _{-}(j, N_{i-1},e_{i-1})n_i^j\rangle -\dfrac{1}{\Delta _t}\langle \beta _{+}(j, N_{i+1},e_{i+1})n_i^j\rangle \nonumber \\&\quad +\dfrac{1}{\Delta _t}\langle \mu _{+}(j-1, N_i,e_i)n_i^{j-1}\rangle +\dfrac{1}{\Delta _t}\langle \mu _{-}(j+1, N_i,e_i)n_i^{j+1}\rangle \nonumber \\&\quad -\dfrac{1}{\Delta _t}\langle \mu _{+}(j, N_i,e_i)n_i^j\rangle -\dfrac{1}{\Delta _t}\langle \mu _{-}(j, N_i,e_i)n_i^j\rangle \nonumber \\&\quad +\dfrac{1}{\Delta _t} \langle \gamma (j, N_i,e_i)n_i^j\rangle . \end{aligned}$$

We now derive a PDE description of Eq. (3) by taking limits as $\Delta _x\rightarrow 0$, $\Delta _y\rightarrow 0$ and $\Delta _t\rightarrow 0$. In order to do this, the discrete values of $\langle {n}_i^j(t_h)\rangle$ and $\langle {e}_i(t_h)\rangle$ are written in terms of the continuous variables *n*(*x*, *y*, *t*) and *e*(*x*, *t*), describing the cell and local environment density, respectively, along with

$$\begin{aligned} \rho (x,t)= \int _{y=Y_{\text {min}}}^{y=Y_{\text {max}}}n(x, y, t) \textrm{d}y, \end{aligned}$$

describing the total cell density. We find that, correct to $\mathcal {O}(\Delta _t)$:

4 $$\begin{aligned} \dfrac{\partial n(x, y, t)}{\partial t}&= \dfrac{1}{\Delta _t}\beta _{+}(y, \rho (x, t),e(x, t))n(x-\Delta _x, y, t)\nonumber \\&\quad +\dfrac{1}{\Delta _t} \beta _{-}(y, \rho (x, t),e(x, t))n (x+\Delta _x, y, t) \nonumber \\&\quad -\dfrac{1}{\Delta _t} \beta _{-}(y, \rho (x-\Delta _x, t),e(x-\Delta _x, t))n(x,y,t) \nonumber \\&\quad -\dfrac{1}{\Delta _t}\beta _{+}(y, \rho (x+\Delta _x, t),e(x+\Delta _x, t))n(x,y,t) \nonumber \\&\quad +\dfrac{1}{\Delta _t}\mu _{+}(y-\Delta _y, \rho (x, t),e(x, t))n(x, y-\Delta _y, t)\nonumber \\&\quad +\dfrac{1}{\Delta _t}\mu _{-}(y+\Delta _y, \rho (x, t),e(x, t))n(x, y+\Delta _y, t)\nonumber \\&\quad -\dfrac{1}{\Delta _t}\mu _{+}(y, \rho (x, t), e(x, t))n(x,y,t) \nonumber \\&\quad -\dfrac{1}{\Delta _t}\mu _{-}(y, \rho (x, t),e(x, t))n(x,y,t)\nonumber \\&\quad +\dfrac{1}{\Delta _t} \gamma (y, \rho (x, t),e(x, t))n(x,y,t). \end{aligned}$$

Employing a Taylor series expansion around (*x*, *y*), rearranging and collecting terms, we obtain

$$\begin{aligned} \dfrac{\partial }{\partial t}n(x,y,t)&=\dfrac{\Delta _x}{\Delta _t}\dfrac{\partial }{\partial x}\Big (\left( \beta _{-}(y, \rho (x,t), e(x,t))-\beta _{+}(y, \rho (x,t), e(x,t))\right) n(x,y,t)\Big ) \\&\quad + \dfrac{\Delta _x^2}{2\Delta _t} \dfrac{\partial }{\partial x}\Bigg (\Big (\beta _{-}\left( y, \rho (x,t), e(x,t)\right) +\beta _{+}\left( y, \rho (x,t), e(x,t)\right) \Big )\dfrac{\partial }{\partial x} n(x,y,t) \\&\quad - n(x,y,t)\dfrac{\partial }{\partial x}\Big (\beta _{-}\left( y, \rho (x,t), e(x,t)\right) +\beta _{+}\left( y, \rho (x,t), e(x,t)\right) \Big )\Bigg ) \\&\quad +\dfrac{\Delta _y}{\Delta _t}\dfrac{\partial }{\partial y} \left( \Big (\mu _{-}\left( y, \rho (x,t), e(x,t)\right) -\mu _{+}\left( y, \rho (x,t), e(x,t)\right) \Big )n(x,y,t)\right) \\&\quad +\dfrac{\Delta _y^2}{2\Delta _t} \dfrac{\partial ^2}{\partial y^2}\left( \Big (\mu _{-}\left( y, \rho (x,t), e(x,t)\right) +\mu _{+}\left( y, \rho (x,t), e(x,t)\right) \Big )n(x,y,t)\right) \\&\quad +\dfrac{1}{\Delta _t}\gamma \left( y, \rho (x,t), e(x,t)\right) n(x,y,t). \end{aligned}$$

We take the parabolic limit as $\Delta _x, \, \Delta _y, \, \Delta _t \rightarrow 0$ simultaneously (assuming $n(x,y,t)\sim O(1)$), and define

$$\begin{aligned} \lim _{\Delta _x, \Delta _t\rightarrow 0} \dfrac{\Delta _x}{\Delta _t} \Big (\beta _{-}(y, \rho (x,t), e(x,t))-\beta _{+}(y, \rho (x,t), e(x,t))\Big )&= v^m(y, \rho (x,t), e(x,t)), \\ \lim _{\Delta _x, \Delta _t\rightarrow 0} \dfrac{\Delta _x^2}{2\Delta _t} \Big (\beta _{-}(y, \rho (x,t), e(x,t))+\beta _{+}(y, \rho (x,t), e(x,t))\Big )&= D^m(y, \rho (x,t), e(x,t)), \\ \lim _{\Delta _y, \Delta _t\rightarrow 0} \dfrac{\Delta _y}{\Delta _t} \Big (\mu _{-}(y, \rho (x,t), e(x,t))-\mu _{+}\left( y, \rho (x,t), e(x,t)\right) \Big )&= v^p(y, \rho (x,t), e(x,t)), \\ \lim _{\Delta _y, \Delta _t\rightarrow 0} \dfrac{\Delta _y^2}{2\Delta _t} \Big (\mu _{-}(y, \rho (x,t), e(x,t))+\mu _{+}\left( y, \rho (x,t), e(x,t)\right) \Big )&= D^p(y, \rho (x,t), e(x,t)), \\ \lim _{\Delta _t\rightarrow 0} \dfrac{1}{\Delta _t} \gamma \left( y, \rho (x,t), e(x,t)\right)&= r(y, \rho (x,t), e(x,t)), \end{aligned}$$

such that the final equation governing the dynamics of the cell population is given by

5 $$\begin{aligned} \dfrac{\partial }{\partial t}n(x,y,t)&= \dfrac{\partial }{\partial x}\Big (v^m(y, \rho (x,t), e(x,t))n(x,y,t)\Big ) \nonumber \\&\quad +\dfrac{\partial }{\partial x}\Bigg (D^m\left( y, \rho (x,t), e(x,t)\right) \dfrac{\partial }{\partial x} n(x,y,t) \nonumber \\&\qquad \qquad \qquad - n(x,y,t)\dfrac{\partial }{\partial x}D^m\left( y, \rho (x,t), e(x,t)\right) \Bigg ) \nonumber \\&\quad +\dfrac{\partial }{\partial y} \Big ( v^p\left( y, \rho (x,t), e(x,t)\right) n(x,y,t)\Big ) \nonumber \\&\quad +\dfrac{\partial ^2}{\partial y^2}\Big ( D^p\left( y, \rho (x,t), e(x,t)\right) n(x,y,t)\Big ) \nonumber \\&\quad +r\left( y, \rho (x,t), e(x,t)\right) n(x,y,t). \end{aligned}$$

The differential equation governing the cell population evolution over time is complemented with the initial condition

6 $$\begin{aligned} n(x,y,0)=n_0(x,y),\end{aligned}$$

and is subject to zero-flux boundary conditions at $x=X_{\text {min}}, X_{\text {max}}$ and $y=Y_{\text {min}}, Y_{\text {max}}$, which are derived in Supplementary Information Sec. S1.1.1 and given by

7 $$\begin{aligned}&v^mn +D^m\dfrac{\partial n}{\partial x}-n\dfrac{\partial D^m}{\partial x} = 0 \qquad \qquad \text {at} \quad x=X_{\text {min}}, \end{aligned}$$

8 $$\begin{aligned}&-v^mn -D^m\dfrac{\partial n}{\partial x}+n\dfrac{\partial D^m}{\partial x} = 0 \qquad \qquad \text {at} \quad x=X_{\text {max}}. \end{aligned}$$

on the physical domain and

9 $$\begin{aligned}&v^pn +\dfrac{\partial }{\partial y}(D^pn)= 0 \qquad \qquad \text {at} \qquad y=Y_{\text {min}}, \end{aligned}$$

10 $$\begin{aligned}&-v^pn -\dfrac{\partial }{\partial y}(D^pn)= 0 \qquad \qquad \text {at} \qquad y=Y_{\text {max}}, \end{aligned}$$

at the ends of phenotype space. The differences in the boundary conditions in phenotype and physical space are observed as a result of the varied assumptions underlying the movement probabilities. In physical space, the probability of movement depends on the surrounding number of cells and elements of the local environment in the target site, whereas the probability of movement in phenotype space depends on the number of cells and elements of the local environment in the same site as the cell.

#### The Coarse-Grained Model of the Local Environment

Using probabilistic approximations of the same form as those underlying Eq. (3), we recover the following equation describing the evolution of elements of the local environment in site *i* over time:

11 $$\begin{aligned} \Delta _t \dfrac{\partial }{\partial t} \langle e_s\rangle =- \sum _{j=1}^{N_y}\langle \lambda (j, n_s^j)e_s\rangle . \end{aligned}$$

Defining

$$\begin{aligned} \lim _{\Delta _t\rightarrow 0}\dfrac{1}{\Delta _t} \lambda (y, n(x,y,t))= \nu (y, n(x, y, t), \end{aligned}$$

which we can substitute into Eq. (11), rearrange and take limits as $\Delta _x, \Delta _y, \Delta _t \rightarrow 0$, to find that the differential equation for the evolution of the density of the local environment, *e*(*x*, *t*), is given by

12 $$\begin{aligned} \dfrac{\partial }{\partial t} e(x,t) = - \int _{y=Y_{\text {min}}}^{y=Y_{\text {max}}} \nu (y, n(x, y, t)) e(x, t) \textrm{d}y. \end{aligned}$$

The corresponding initial condition is then

13 $$\begin{aligned} e(x,0)=e_0(x). \end{aligned}$$

Now that we have derived the coarse-grained model in full (Eqs. (5)-(10), (12) and (13)), we present a series of applications that demonstrate the utility of this framework through the choice of specific functional forms for the functions $v^m\left( y, \rho (x,t), e(x,t)\right)$, $D^m\left( y, \rho (x,t), e(x,t)\right)$, $v^p\left( y, \rho (x,t), e(x,t)\right)$, $D^p(y, \rho (x,t), e(x,t))$, $r (y, \rho (x,t), e(x,t))$ and $\nu (y, n(x,y,t))$. We will assume in this article that all movement in physical space is undirected, and therefore we take $\beta _{+}(y, \rho (x,t), e(x,t))=\beta _{-}(y, \rho (x,t), e(x,t)$, such that $v^m=0$ hereon in. Nevertheless, the general form of the governing equations is retained, enabling readers to readily adapt the framework to cases involving directed movement or other specific applications.

## Broad Spectrum Applications in Mathematical Biology

In this section, we showcase the versatility of the PDE modelling framework given by Eqs. (5)-(10), (12) and (13) by applying it to several exemplar biological scenarios. These applications demonstrate how the PDE framework effectively captures emergent population-level dynamics across diverse biological contexts. By considering a range of different underlying characteristics and interaction rules (prescribed in Supplementary Information Sec. S2), we showcase the ability of these models to encode complex behaviours while maintaining analytical and computational tractability.

### Simulation Methods

The deterministic, continuum counterpart of the individual-based model described in Sec. 2 is given by the PDEs in Eqs. (5) and (12), with boundary conditions given in Eqs. (7)-(10) and initial conditions given in Eqs. (6) and (13). To solve this system numerically, we use an advection-diffusion-reaction (A-DR) scheme that discretises the spatial variable *x* using a central finite difference stencil modified from previous work (Crossley et al. 2023), employing ghost points to enforce the zero-flux boundary conditions. The full system of discretised equations can be found in the Supplementary Information. In the phenotypic axis, *y*, we use a finite volume scheme, which divides the axis into $N_y+1$ sites of equal width. The advective component is controlled using the Koren limiter (Koren 1993). The resulting system of ordinary differential equations are then integrated in time using python’s in-built ordinary differential equation solver scipy.integrate.solve_ivp with the explicit Runge-Kutta integration method of order 5 and time step $\Delta _t=0.1$. The phenotype step is $\Delta _y=0.02$ and the spatial step is $\Delta _x=0.1$, both of which were chosen to be sufficiently small to ensure that we observed convergence in the solutions. Where constant speed, constant profile travelling waves are observed, the speed is estimated numerically by saving the solution at each time point, interpolating to find the critical spatial position $x^*$ such that $\rho (x^{*}, t)=0.1$ and then calculating the difference between two sequential critical spatial points and dividing by the time step between them. At each spatial position, the mean phenotype is obtained by computing the density-weighted sum of phenotypes and normalising by the total local density. Code is available for all computations at the following GitHub repository: https://github.com/beckycrossley/cont_phen.

### Phenotypic Structuring during Range Expansion

Understanding how cell populations expand and evolve is a fundamental question in biology, particularly in contexts such as tumour growth, microbial colony expansion, and tissue development. A key aspect of these processes is tracking cell lineages to uncover how phenotypic traits propagate and shape population dynamics over time. In this section, we demonstrate how this modelling framework provides a convenient and effective approach for studying these lineage dynamics within an evolving population. Specifically, we consider a phenotypically structured population of homogeneous cells (*i.e.*, cells that share the same underlying behaviour but are distinguishable by a phenotypic marker) to gain deeper insights into how individual lineages contribute to the overall invasion process. By analysing the spatio-temporal evolution of the phenotypic structure as the population spreads, we highlight how this approach enables the systematic tracking of cell lineages during range expansion.

Previous studies, such as those by Marculis et al. (2020), have investigated similar population dynamics using stage-structured integrodifference equations (Marculis and Lewis 2020), with further extensions incorporating trade-offs between reproductive and dispersal abilities (Marculis et al. 2020). While these approaches offer valuable insights into structured population dynamics, they rely on discrete phenotypic stages, which may limit the resolution of evolutionary and ecological interactions.

In contrast, this work provides a more nuanced perspective by modelling the evolution of a continuously structured phenotype, $y\in [0,1]$, over space, $x\ge 0$, and time, $t\ge 0.$ This allows for a finer representation of phenotypic variation and subsequent exploration of its role during population expansion. Specifically, we describe the spatio-temporal evolution of the cell population, *n*(*x*, *y*, *t*), using the following governing equation:

14 $$\begin{aligned} \dfrac{\partial }{\partial t} n(x,y,t) = \dfrac{\partial ^2}{\partial x^2} n(x,y,t) + r\left( \rho (x,t)\right) n(x,y,t). \end{aligned}$$

As in Marculis et al. (2020), we study Eq. (14) subject to two different functions describing net cell proliferation:

15 $$\begin{aligned} r_K(y, \rho (x,t)) = 1-\rho (x,t), \end{aligned}$$

for Fisher-KPP type invasion (pulled waves) and

16 $$\begin{aligned} r_A(y, \rho (x,t)) = (1-\rho (x,t))(\rho (x,t)-p^{*}), \end{aligned}$$

for the Allee effect, with $p^{*}\in (0, 1/2)$ (corresponding to pushed waves), where $\rho (x,t)=\int _{y=0}^{y=1}n(x,y,t)\textrm{d}y.$ The individual-based functions underlying these continuum equations can be found in Supplementary Information Sec. S2.1. We compliment this setup with an initial condition that ensures initial phenotypic structuring of the population, which can then be tracked over time. Specifically, we take

17 $$\begin{aligned} u_0(x,y)={\left\{ \begin{array}{ll} 1 \quad \text {if}\;\; {{x=5y}}, \\ 0 \quad \text {otherwise.} \end{array}\right. } \end{aligned}$$

**Fig. 1:**
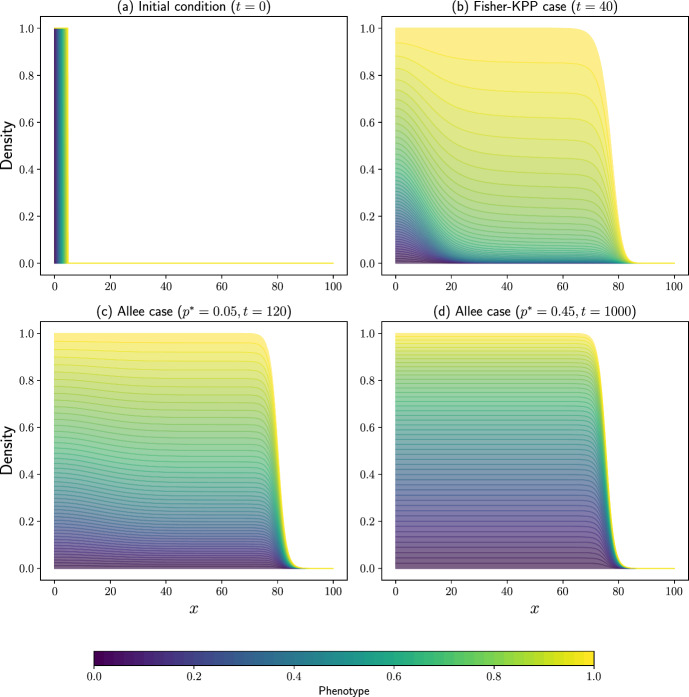
Evolution of the phenotypic structure of cells in Eq. (14) subject to various growth terms. (a) The initial distribution of the cells with different phenotypes. (b) The spatial structure of the invading wave subject to the Fisher-KPP growth term (Eq. (15)). Results in (c) and (d) show the spatial structure of the invading wave subject to the Allee effect (Eq. (16))

Recalling that cells in this case are homogeneous, and thus the phenotypic variable $y\in [0,1]$ is used purely to label cells as they evolve, we can see that Fig. 1 shows the phenotypic structure of the population of cells as they invade, subject to the aforementioned growth terms (Eq. (15) and Eq. (16)). Specifically, Fig. 1(b) shows the spatial structure of Eq. (14) with the Fisher-KPP growth term (Eq. (15)). As invasion progresses, the leading edge of the expanding population is dominated by cells that originated from the rightmost part of the initial distribution, with phenotypes closer to $y=1$. Over time, the density of these dominant phenotypes increases, and due to diffusion, cells with these traits spread backward, integrating into the bulk of the population.

This is a form of what is known as *surfing* (Klopfstein et al. 2006)–a phenomenon well-studied in the context of drifting genetic mutations in expanding populations. Surfing occurs when areas of low cell density allow space for increased growth, such as along the invading front (see Fig. 1) (Excoffier et al. 2009).

The structure observed in the case of the Fisher-KPP type growth is often described as a ‘vertical pattern’ (Marculis et al. 2020), which indicates that the total population is composed of different underlying phenotypes at different points in space, demonstrating a high level of spatial structuring. However, we see that even in a continuously structured cell population, it is those cells with the phenotypes that were nearest the front of the initial distribution of cells that dominate during range expansion. The lower phenotypes, which began at the rear of the invading population, primarily remain there and diminish in size over time and increasing space.

Alternatively, if we now consider the case of Allee growth (Eq. (16)) in Eq. (14), then the numerical results in Fig. 1 display a different pattern of invasion. As the cells invade, a much larger proportion of all of the initially present phenotypes remain present in the travelling wave front at later times, and throughout the bulk of the invading population. As in the Fisher-KPP case, the cells in the rightmost portion of the initial population, with phenotypes near 1, contribute the largest portions to the wave. However, with the addition of the Allee effect, there now exist contributions from all initially present phenotypic states throughout the wave. This is because the Allee effect introduces a dependency on the total local population density, which means cells at the leading edge, in areas of low density, have reduced proliferation, preventing them from rapidly outcompeting other phenotypes within the cell bulk. We note that by increasing the strength of the Allee effect, by taking $p^{*}$ closer to 0.5, the proportion of all of the phenotypes present in the wave becomes closer to one another (equalises). The spatial pattern observed in this case is described as ‘horizontal’ as it does not differ in space, but still shows high phenotypic variation in the front (Roques et al. 2012).

These structural differences at the wave front agree with those observed by Roques et al. (2012) who consider a similar system but with discrete, rather than continuous, phenotypes. These results indicate that the lineage structure of an invading wave could potentially be used to distinguish between the underlying growth mechanisms of the cell populations. It is also notable that the speed of cell invasion subject to the Allee effect (Eq. (16)) is significantly slower than the speed of invasion in the Fisher-KPP case (Eq. (15)). As such, although the Allee effect maintains diversity across the travelling wave front, it also decreases the speed of invasion in doing so, and so a trade-off is observed between diversity maintenance and speed of population invasion, which favours faster invasion for a weaker Allee effect.

### A Go-Or-Grow Model of Cells Invading the Extracellular Matrix (ECM)

In this section, we apply the aforementioned system of Eqs. (5)-(10), (12) and (13) to a general model for collective cell migration into the ECM, exploring how individual cell-level properties give rise to emergent population-level behaviours. By examining the interactions between cells and their environment at a population-level, we aim to uncover the underlying individual cell-ECM mechanisms that drive large-scale migration patterns and spatial organization, providing insight into how cell processes shape collective movement in biological systems.

The ECM is a complex network of proteins and carbohydrates that supports and provides structure for migrating cells (Winkler et al. 2020; Crossley et al. 2024a). Its composition varies by location, making its role in collective migration difficult to define. However, a well-agreed notion is that cells need to breakdown the ECM in order to make space in which to invade (Nagase and Woessner 1999; Visse and Nagase 2003; Nagase et al. 2006; Jabłońska-Trypuć et al. 2016).

In this work, we aim to model the fundamental trade-off in energetic costs associated with motility and proliferation, known as the “go-or-grow" hypothesis (Hatzikirou et al. 2012), while also incorporating the role of ECM degradation by migrating cells. Our goal is to use this framework to bridge the gap between individual-cell behaviours and emergent population-level dynamics.

Previous mathematical models have captured this trade-off by considering only two discrete phenotypes (Crossley et al. 2024b), due to limitations in the available mathematical frameworks. However, biological evidence strongly supports the existence of a continuum of phenotypes rather than a simple dichotomy (Bendall and Nolan 2012; Campbell et al. 2021). By extending prior work to a continuously structured phenotypic model, we provide a more biologically realistic representation of go-or-grow dynamics, allowing us to explore how individual-level decision-making translates into large-scale invasion patterns.

We model the cells, denoted *n*(*x*, *y*, *t*), as able to move, proliferate and degrade the ECM, *e*(*x*, *t*). To model the trade-off between ECM degradation, motility and proliferation, we assume that cells with a larger value of the phenotype variable, $y\in [0,1]$, have a higher proliferative potential but a lower motility and lower ECM degrading potential whilst, in comparison, cells with a lower value of the phenotypic variable *y* correspond to those with a higher motility and higher ECM degrading potential, but a lower proliferative potential. Therefore, cells with phenotype $y=0$ degrade the ECM most rapidly and are the most motile, but they are unable to proliferate. On the other hand, cells with phenotype $y=1$ are the most proliferative, but cannot degrade the ECM or move. As in Crossley et al. (2024b), we implement a linear relationship between the phenotype of the cells and the associated ability to degrade the ECM, move and proliferate.

The initial functions describing the probabilities of transitions in the individual-based model underlying these continuum equations can be found in Supplementary Information Sec. S2.2. Hereon in, we focus on the corresponding continuum functions stated below.

Following the volume exclusion principles described by Crossley et al. (2023, 2024b), we assume that the probability of a cell moving randomly in physical space linearly decreases as the occupancy of space increases. As described earlier, we also introduce the assumption that the probability of a cell moving randomly increases as the phenotype of the cell decreases. As such, the function describing movement in physical space can be written as

$$\begin{aligned} D^m(y, \rho (x,t), e(x,t)) = (1-y)\left( 1-\dfrac{\rho (x,t)+e(x,t)}{\kappa }\right) , \end{aligned}$$

where $\kappa >0$ is the total available density for cells and ECM, known as the carrying capacity. Similarly, for all cells, the probability of cell proliferation linearly decreases as the space around the cell fills with other cells and ECM, but it also decreases as the phenotype decreases, such that

$$\begin{aligned} r(y, \rho (x,t), e(x,t)) = y\left( 1-\dfrac{\rho (x,t)+e(x,t)}{\kappa }\right) . \end{aligned}$$

Furthermore, we assume that the degradation rate of the ECM is proportional to the density of surrounding cells and decreases as the phenotype of the cells increases. We write this as

$$\begin{aligned} \nu (y, n(x, y, t)) = (1-y)n(x,y,t). \end{aligned}$$

One major challenge in understanding the cell phenotype dynamics during invasion is the lack of direct experimental observations, as visualizing phenotypic transitions in real time is extremely difficult. As a result, there is limited guidance in the literature on the appropriate mathematical forms for these transitions, leaving a key gap in the understanding of how phenotype-dependent behaviours shape collective migration.

This highlights the final undetermined functions in Eqs. (5) and (12), that describe the transitions between cell phenotypes. In this section, we systematically investigate the impact of different density-dependent phenotypic transition rules, $\mu _{\pm }(y, \rho (x,t), e(x,t))$, along with their associated drift and diffusion terms, $v^p(y, \rho (x,t), e(x,t))$ and $D^p(y, \rho (x,t), e(x,t))$, respectively, as summarised in Table 1. By exploring how the invasion dynamics change under different assumptions, we aim to determine whether population-level (and therefore potentially more observable) behaviours can provide insight into the underlying, unobservable phenotypic structures, offering a potential approach for inferring hidden biological mechanisms from macroscopic invasion patterns.

**Table 1:** Table listing the functions employed in Eq. (5) describing the probabilities of transitions up and down in phenotype space, resulting in the phenotypic drift, ${{v^p}}(y, \rho (x,t), e(x,t))$, and phenotypic diffusion, ${{D^p}}(y, \rho (x,t), e(x,t))$, functions shown

Phenotypic drift	$\mu _{-}(y, \rho , e)$	$\mu _{+}(y, \rho , e)$	$v^p(y, \rho ,e)$	$D^p(y, \rho , e)$
Cell-dependent	$\dfrac{\rho }{\kappa }$	$1-\dfrac{\rho }{\kappa }$	$2\dfrac{\rho }{\kappa }-1$	1
ECM-dependent	$\dfrac{e}{\kappa }$	$1-\dfrac{e}{\kappa }$	$2\dfrac{e}{\kappa }-1$	1
Space-dependent	$\dfrac{\rho +e}{\kappa }$	$1-\dfrac{\rho +e}{\kappa }$	$2\dfrac{\rho +e}{\kappa }-1$	1

We investigate three phenotypic drift mechanisms influencing cell transitions: firstly, cell-dependent drift, where cells shift toward more motile phenotypes at higher cell densities; next, ECM-dependent drift, where cells adopt more ECM-degrading phenotypes as ECM density increases; and finally, space-dependent drift, where cells become more proliferative as available space increases. Each drift term is bounded between zero and one, which is consistent with the constraints placed on the total cell and ECM density. By comparing these mechanisms for phenotype change, we assess how different environment- and density-dependent phenotypic transitions shape the structure of the migrating cell front.

We simulate this system of Eqs. (5)-(10), (12) and (13) subject to the following initial conditions:

18 $$\begin{aligned} n_0(x,y)&= \dfrac{{\text {exp}\left( -100(x^2 + y^2)\right) }}{\max \left( \int _{X_{\text {min}}}^{X_\text {max}} {\text {exp}\left( -100(x^2 + y^2)\right) } \, \textrm{d}x \right) }, \end{aligned}$$

19 $$\begin{aligned} e_0(x)&={\left\{ \begin{array}{ll} 0 \quad \quad \text {if} \;\; n_0(x,y)>0.001, \\ 0.5 \quad \text {otherwise.} \end{array}\right. } \end{aligned}$$

**Fig. 2:**
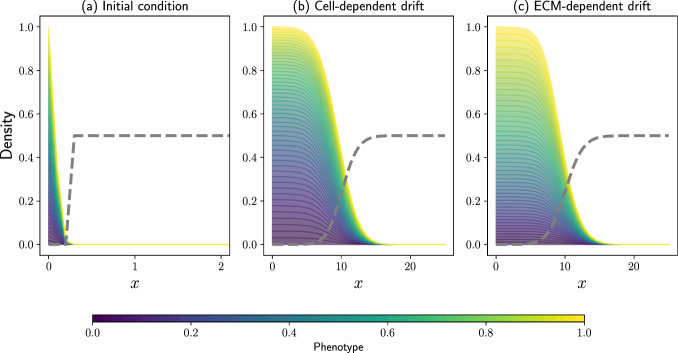
Evolution of the phenotypic structure of cells in Eqs. (5)–(12) subject to various phenotypic drift terms, with the corresponding ECM density shown as a dashed grey line. (a) The initial distribution of the ECM and the cells with different phenotypes. (b) The spatial structure of the invading wave subject to cell-dependent phenotypic drift. (c) The spatial structure of the invading wave subject to ECM-dependent phenotypic drift. Results in (b) and (c) are all plotted at $t=30$ and simulations are carried out with $\kappa =1$. See Table 1 for explicit forms of the phenotypic drift terms

By examining the simulation results shown in Fig. 2 we can see that for a fully mixed initial population of cells, the phenotypic drift terms considered produce travelling wave solutions with similar, constant speeds of invasion. See Supplementary Information Figs. S1 and  S2 for a comparison including space-dependent phenotypic drift, which shows very similar results to cell-dependent drift.

In the case of cell-dependent phenotypic drift, we notice that there is a larger density of cells with lower phenotypes, which correspond to more motile and ECM degrading but less proliferative cells, in the bulk of the wave. At the front, we instead see a higher proportion of more proliferative and immobile cells, which are able to divide into the available space. As such, in the bulk of the wave the mean phenotype remains the same but as *x* increases, we observe a gradual increase in mean phenotype at the wave front where extremely low cell densities induce cells to switch to a proliferative phenotype. Very similar patterning is observed in the space-dependent phenotypic drift case, without the sharp change in phenotype at the very front of the travelling wave (results in Supplementary Information). In this context, this model predicts that the density of the ECM has minimal impact on the phenotypic structure of the invading wave.

However, when examining ECM-dependent phenotypic drift in Fig. 2(c), we notice that the cells with higher phenotypes, corresponding to less motile and less degrading but more proliferative, now constitute a larger proportion of the population behind the invading front. Compared to cell- or space-dependent phenotypic drift, the mean phenotype of cells in the bulk is significantly higher. However, at the front of the invading wave in the ECM-dependent case, we see a larger proportion of cells with low phenotypes, namely, cells that have a higher ability to degrade the ECM and move into the subsequently available space.

As a result of the phenotypic structures developed when the system is subject to different phenotypic drift functions, the spatial structure of the individual phenotypes within the invading wave could be used to better understand the underlying mechanisms governing phenotypic transitions during collective cell migration into the ECM.

### T Cell Exhaustion

In this subsection, we demonstrate the broad applicability of the framework by deriving a population-level PDE model for T cell exhaustion, starting from an individual-based description of the underlying dynamics. This approach systematically coarse-grains the underlying cell processes, ensuring that the resulting PDEs are not merely phenomenological but instead retain a direct mechanistic link to individual cell properties. Specifically, we incorporate a T cell population with varying levels of exhaustion invading into a tumour, and examine the role of phenotype-dependent drift in shaping its exhaustion dynamics. This application highlights how this framework can be adapted to capture complex immune responses while preserving biologically meaningful connections between individual- and population-level behaviour.

T cells are a key component of the immune system, with an important role to play in locating and attacking tumour cells (Weninger et al. 2001). When space is available, T cells will infiltrate into the tumour where they kill malignant cells by releasing cytotoxic enzymes. During the sustained activation of T cells required to combat and restrict further growth of a tumour, T cells will differentiate and eventually “exhaust” (Yi et al. 2010). This occurs as a result of continued exposure to the antigens of the tumour cells and as T cells exhaust, their effector functions reduce (Blank et al. 2019; Chow et al. 2022). Exhaustion results in diminished cytokine production, impaired proliferation and reduced motility in the T cells (Miller et al. 2019). Completely exhausted T cells are no longer able to move or grow (Wherry and Ahmed 2004), and have impaired toxicity, which reduces their ability to kill off tumour cells (Jiang et al. 2015). Furthermore, T cells will die much faster the more exhausted they are (Wherry and Ahmed 2004). Understanding the dynamics of the T cells as they infiltrate a tumour and their exhaustion during this process is crucial to developing treatments for tumours, and so we develop a mathematical model to gain further insights.

As such, after applying the T cell specific individual-based model functions described in the Supplementary Information Sec. S2.3 to the coarse-graining process, we investigate the spatio-temporal evolution of T cell density, denoted *T*(*x*, *y*, *t*), invading into a population of tumour cells, with density denoted by *C*(*x*, *t*). Similarly to before, we define $\rho (x,t)$ as

$$\begin{aligned} \rho (x,t)= \int _{y=Y_{\text {min}}}^{y=Y_{\text {max}}}T(x, y, t) \textrm{d}y. \end{aligned}$$

In this application, the phenotype of the cells, $y\in [0,1]$, represents the exhaustion of the T cells. As such, T cells with phenotype $y=1$ are naïve, and are able to move freely and randomly in physical space, whilst also attacking nearby tumour cells and dividing (Worbs and Förster 2009; Reina-Campos and Goldrath 2019). However, T cells with phenotype $y=0$ are considered exhausted, or terminally-differentiated memory T cells, which are considered to be in a resting state (Sprent and Surh 2011; van den Broek et al. 2018). The resulting model takes the following form:

20 $$\begin{aligned} \dfrac{\partial }{\partial t}T(x,y,t)&= \dfrac{\partial }{\partial x}\bigg ({{D^m}}\big (y, \rho (x,t), C(x,t)\big )\dfrac{\partial }{\partial x} T(x,y,t) \nonumber \\&\quad \qquad \qquad - T(x,y,t)\dfrac{\partial }{\partial x}{{D^m}}\big (y, \rho (x,t), C(x,t)\big )\bigg ) \nonumber \\ &\quad +\dfrac{\partial }{\partial y} \bigg ({{v^p}}\big (y, \rho (x,t), C(x,t)\big )T(x,y,t)\bigg )\nonumber \\ &\quad +\dfrac{\partial ^2}{\partial y^2}\bigg ({{D^p}}\big (y, \rho (x,t), C(x,t)\big )T(x,y,t)\bigg )\nonumber \\ &\quad +{{r}}\big (y, \rho (x,t), C(x,t)\big )T(x,y,t), \end{aligned}$$

where the net proliferation of the T cells, which depends on the exhaustion of the T cells and the available surrounding space for growth, can be described by

$$\begin{aligned} {{r}}(y, \rho (x,t), C(x,t)) = \gamma _1y\left( 1-\frac{\rho (x,t)+C(x,t)}{\kappa }\right) -\gamma _0(1-y), \end{aligned}$$

where $\gamma _1\ge 0$ describes the growth rate, and $\gamma _0\ge 0$ describes the death rate of the T cells. $\kappa >0$ is the carrying capacity for the cells, as described in Sec. 3.3.

T-cell movement in physical space is assumed to be random and undirected, but it is prevented by the presence of other T cells or tumour cells (in line with the volume-filling assumptions described in Sec. 3.3). As such, diffusion in physical space is given by

$${{D^m}}(y, \rho (x,t), C(x,t)) = y \bigg (1-\dfrac{\rho (x,t)+C(x,t)}{\kappa }\bigg ),$$

which describes a higher diffusion rate for T cells with a higher phenotype.

Furthermore, we wish to model the phenotypic transitions of the T cells, or how exhausted the T cells become as they move, grow and interact with tumour cells. We have already assumed that the higher the phenotype of a T cell, the higher its probability of moving and growing, and this in turn will exhaust it. We also know that the tumour cells can be killed by the T cells, which we assume will further exhaust the T cells at a rate proportional to the number of interactions they have with the surrounding tumour cells. Therefore, we model the drift in phenotype space as

$${{v^p}}(y, \rho (x,t), C(x,t)) = y(k_1+k_2C(x,t)),$$

where $k_1, k_2 \ge 0$ describe the exhaustion rate of the T cells as a result of movement and growth, and as a result of interactions with the tumour cells, respectively. We allow for some small randomness in the exhaustion levels of the T cells by including a diffusive term in phenotype space of the form

$$\begin{aligned} {{D^p}}(y, \rho (x,t), C(x,t))=\varepsilon \ll 1. \end{aligned}$$

Now, it is well-known that tumour cells are also mobile and able to grow (Suresh 2007). As such, we can derive an equation similar to Eq. (12) which also includes terms describing random movement and proliferation derived in the same manner as those in Eq. (5). The resulting equation governing the evolution of the density of the tumour cells in space, $x\ge 0$, and time, $t\ge 0$, is therefore given by

21 $$\begin{aligned} \dfrac{\partial }{\partial t} C(x,t)&= \dfrac{\partial }{\partial x}\bigg ({{D^C}}\big (\rho (x,t), C(x,t)\big )\dfrac{\partial }{\partial x} C(x,t) \nonumber \\&\qquad \qquad \qquad - C(x,t)\dfrac{\partial }{\partial x}{{D^C}}\big (\rho (x,t), C(x,t)\big )\bigg ) \nonumber \\ &\quad -\int _{y=0}^{y=1}{{\nu }}(y, T(x,y,t)) C(x,t) \text {d}y + {{g}}(\rho (x, t), C(x,t))C(x,t). \end{aligned}$$

We note here that the term $D^C(\rho (x,t), C(x,t))$ behaves similarly to $D^m(y, \rho (x,t), C(x,t))$ and that ${{g}}(\rho (x,t), C(x,t))$ behaves like the growth term in the previous application, but without the phenotype dependence.

Assuming that the motility and proliferation of tumour cells is restricted in regions of high cell density, we take

$$\begin{aligned} {{g}}(\rho (x,t), C(x,t))&=r_C\left( 1-\dfrac{\rho (x,t)+C(x,t)}{\kappa }\right) , \\ {{D^C}}(\rho (x,t), C(x,t))&=1-\dfrac{\rho (x,t)+C(x,t)}{\kappa }, \end{aligned}$$

with $r_C\ge 0$ describing the growth rate of the tumour cells. Finally, T cells with a higher phenotype are less exhausted, and therefore kill tumour cells at a higher rate. As such, we write

$$\begin{aligned} {{\nu }}(y, T(x,y,t)) = \bar{\lambda }yT(x,y,t), \end{aligned}$$

where $\bar{\lambda }\ge 0$ describes the rate of degradation of the tumour cells by the T cells, or the toxicity of the T cells on the tumour.

We simulate the system of Eqs.  (20)–(21) subject to the following initial conditions:

22 $$\begin{aligned} T_0(x,y)&= \dfrac{\text {exp}\left( -100(x^2+(y-1)^2)\right) }{\max \left( \int _{X_{\text {min}}}^{X_\text {max}} \text {exp}\left( -100(x^2+(y-1)^2)\right) \, \textrm{d}x \right) }, \end{aligned}$$

23 $$\begin{aligned} C_0(x)&={\left\{ \begin{array}{ll} 0 \quad \quad \text {if} \;\; n_0(x,y)>0.001, \\ 0.5 \quad \text {otherwise.} \end{array}\right. } \end{aligned}$$

**Fig. 3:**
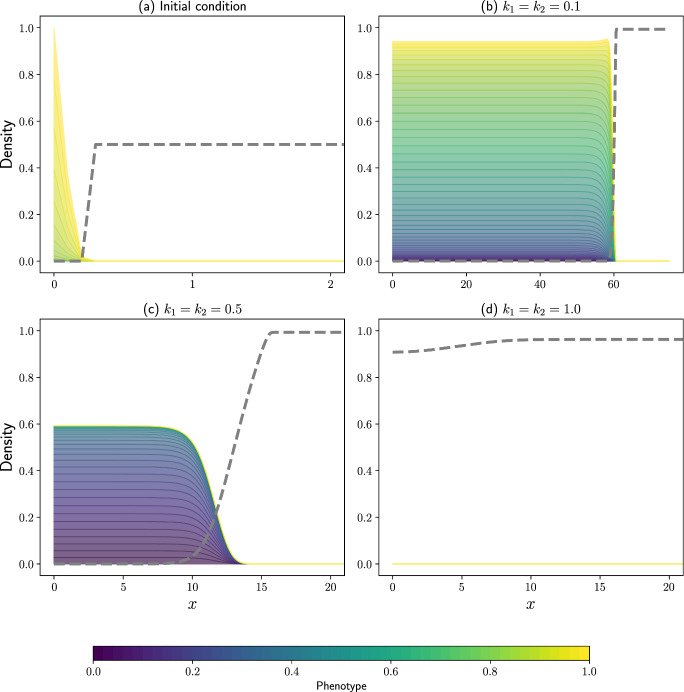
Evolution of the phenotypic structure of population of T cells invading into population of tumour cells as described in Eqs. (20)–(21) subject to various exhaustion rates, with the corresponding tumour cell density shown as a dashed grey line. (a) The initial distribution of the cells. Results in (b), (c) and (d) show the spatial structure of the invading wave subject to exhaustion rates 0.1, 0.5 and 1.0, respectively. Solutions are plotted at $t=50$, and simulations are carried out with $\varepsilon =0.01$, $\gamma _0=1$, $\gamma _1=10$, $r_C=0.1$, $\kappa =1$, and $\bar{\lambda }=10$

Examining Fig. 3 it is clear that two main invasion behaviours are exhibited, which depend on the parameters of the system. The first, which can be observed in Fig. 3(b) and Fig. 3(c), shows T cells that attack the tumour cells and produce travelling wave type profiles that invade through the tumour cells into the domain, killing off the tumour as they do so.

In these simulations, altering the parameters of the system, namely the exhaustion rate of the T cells, leads to a range of different predicted behaviours. The initial population of T cells were naïve. As such, the phenotypic structure of the invading wave of T cells with a lower exhaustion rate consisted of a larger range of phenotypes of cells (see Fig. 3(b)). Consequently, the invading wave retains cells with a high phenotype. Comparatively, by increasing the exhaustion rate of the T cells, a travelling wave of invasion can still be observed (see Fig. 3(c), for example), but with a much more restricted set of phenotypes in the bulk of the wave. The very front of the wave contains T cells with higher phenotypes that are the least exhausted, but the total density of T cells invading into the tumour cells is decreased, and subsequently, so is the invasion speed.

In another scenario, whereby the growth of the tumour cells exceeds the degradation of the tumour cells by the T cells, results similar to those in Fig. 3(d) are found, where the T cells exhaust extremely quickly as a result of a high number of interactions with tumour cells. This continued exposure quickly exhausts the T cells, which in turn increases death and creates available space for subsequent colonization by the tumour cells. In Fig. 3(d), all of the T cells have died and we observe that the tumour cells (plotted as a dashed grey line) are growing to fill the domain far ahead of the location of the initial population of T cells.

Due to the large number of parameters in Eqs. (20)–(21), complete exhaustion and death of the T cells can also be observed by decreasing the diffusion coefficient of the T cells, increasing (decreasing) the death (growth) rate of the T cells, decreasing the toxicity of the T cells or increasing the growth or motility rates of the tumour cells. In all of these cases, the tumour cells will eradicate the T cells and growth of the tumour to fill the available space will be observed. This is an example of competitive exclusion (Strobl et al. 2020). The opposite effects on each of these parameters provides examples of when the T cells are able to either completely eradicate or prevent further growth of the tumour cells. These behaviours have been observed experimentally (Schreiber et al. 2011) and thus the modelling results shown in this work could provide useful insights into developing treatments for tumours in the future, once biologically realistic parameter sets are investigated.

## Discussion

In conclusion, numerous biological processes can be described in a simple form by phenotypically structured cell migration. Yet, general frameworks for modelling this, built from vastly adaptable underlying individual-based principles, are largely understudied (Villa et al. 2024). In this article, we have demonstrated how to derive a general model for phenotypically structured cell migration from the underlying individual-based processes, that can be used to reproduce a variety of results in the literature, whilst modelling a continuum of phenotypes.

Whilst we have illustrated that the general modelling framework is easily adaptable and can be applied to a range of biological systems considering spatial invasion of structured populations, we highlight the need for adaptation of the underlying assumptions to the specific modelling question of interest. For instance, the continuum model derived in this work employed distinctly different boundary conditions in the physical and phenotypic domains. These differences arose from the varying assumptions about the processes governing movement in each domain. However, these conditions can be straightforwardly modified through analogous derivations. We therefore stress that this work represents an important first step towards generalising this modelling approach.

In changing the form of the phenotypic drift terms, care must also be taken to ensure that limits taken in moving from the individual-based derivation to the continuum model are satisfied. For reaction-diffusion models with drift terms, the impact of these scalings and quantitative comparisons between the individual-based and resulting continuum models in such limits are well-studied in the literature (Lorenzi et al. 2020; Macfarlane et al. 2022).

The continuum modelling framework presented in this work describes invasion in two dimensions: space and phenotype. We could extend this into higher dimensions, both spatially and phenotypically, such that two or three dimensional experiments, such as those looking at the evolution of tumour spheroids for example, can be more accurately modelled, and the results validated against data. An example of a two-dimensional phenotype space could be the separation of proliferation and motility into two different dimensions, rather than the one-dimensional trade-off discussed in the example in this work.

Beyond this, we have applied this system of Eqs. (5)-(10), (12) and (13) to several biological scenario to demonstrate its versatility and utility. We recognise that these applications are for illustrative purposes only, and thus we employ several biological simplifications. We therefore propose that this framework could provide a base model for continuum modelling of invasion processes derived from underlying individual-based assumptions. The modelling framework presented would therefore benefit from expanding or adapting the forms of the functions to the specific biological application of interest, and the addition of extra details from various sub-models in the literature that may have been validated with experimental data would be needed in order to use this modelling approach to infer specific conclusions about the application of interest. For example, in the go-or-grow application, we have considered the volume-filling effects of both the cells and the ECM. However, if we were to consider modelling a chemoattractant, for example, the volume-filling assumptions underlying this model would no apply to the chemoattractant itself. As such, the derivation of the model would need to be adjusted accordingly, using the same methodology to determine the resulting population-level model.

Additionally, in the case of T cell infiltration into tumour cells, where we interpret the phenotype of the cell as its exhaustion level, we are making a first step into the spatio-temporal modelling of T cell movement with phenotypic structuring, and there remains a large number of questions to be asked. For example, what are the specific functions that most accurately describe the exhaustion of the T cells, or what does it really mean to be exhausted in this context (Blank et al. 2019)?

Overall, when simulation results from a particular biological scenario present vastly different phenotypically structured solutions, this general modelling framework could provide a useful tool for developing understanding of the mechanisms underlying heterogeneity during migration, such as the bet-hedging strategies observed as a result of environmental pressures in Almeida et al. (2024). The framework presented in this work has been derived from descriptions of interactions at an individual-based level and can be easily adapted to investigate a variety of biological applications. As a result, the versatility of this tool could help us understand the role of heterogeneity in a wide range of circumstances and its resulting insights could ultimately be used to help inform subsequent important treatment decisions.

## Supplementary Information

Below is the link to the electronic supplementary material.

Supplementary file 1 (pdf 761 KB)

## References

[CR1] Almeida L, Denis JA, Ferrand N et al (2024) Evolutionary dynamics of glucose-deprived cancer cells: insights from experimentally informed mathematical modelling. J R Soc Interface 21(210):20230587 38196375 10.1098/rsif.2023.0587 PMC10777142

[CR2] Anderson A, Rejniak K (2007) Single-cell-based models in biology and medicine. Springer Science & Business Media

[CR3] Ardaševa A, Anderson AR, Gatenby RA et al (2020) Comparative study between discrete and continuum models for the evolution of competing phenotype-structured cell populations in dynamical environments. Phys Rev E 102(4):042404 10.1103/PhysRevE.102.042404 PMC10900972 33212726

[CR4] Arnold A, Desvillettes L, Prévost C (2012) Existence of nontrivial steady states for populations structured with respect to space and a continuous trait. Commun on Pure and App Anal 11(1):83–96

[CR5] Bendall SC, Nolan GP (2012) From single cells to deep phenotypes in cancer. Nat Biotechnol 30(7):639–647 22781693 10.1038/nbt.2283

[CR6] Berestycki N, Mouhot C, Raoul G (2015) Existence of self-accelerating fronts for a non-local reaction-diffusion equations. arXiv preprint arXiv:1512.00903

[CR7] Blank CU, Haining WN, Held W et al (2019) Defining ‘T cell exhaustion’. Nat Rev Immunol 19(11):665–674 31570879 10.1038/s41577-019-0221-9 PMC7286441

[CR8] Bouin E, Calvez V, Meunier N et al (2012) Invasion fronts with variable motility: phenotype selection, spatial sorting and wave acceleration. Comptes Rendus Math 350(15–16):761–766

[CR9] van den Broek T, Borghans JA, Van Wijk F (2018) The full spectrum of human naive T cells. Nat Rev Immunol 18(6):363–373 29520044 10.1038/s41577-018-0001-y

[CR10] Byrne H, Drasdo D (2009) Individual-based and continuum models of growing cell populations: a comparison. J Math Biol 58:657–687 10.1007/s00285-008-0212-0 18841363

[CR11] Campbell NR, Rao A, Hunter MV et al (2021) Cooperation between melanoma cell states promotes metastasis through heterotypic cluster formation. Dev Cell 56(20):2808–2825 10.1016/j.devcel.2021.08.018 PMC8551056 34529939

[CR12] Carrillo JA, Lorenzi T, Macfarlane FR (2024) Spatial segregation across travelling fronts in individual-based and continuum models for the growth of heterogeneous cell populations. arXiv preprint arXiv:2412.08535 10.1007/s11538-025-01452-y PMC12089248 40388053

[CR13] Celora GL, Byrne HM, Zois CE (2021) Phenotypic variation modulates the growth dynamics and response to radiotherapy of solid tumours under normoxia and hypoxia. J Theor Biol 527:110792 34087269 10.1016/j.jtbi.2021.110792

[CR14] Celora GL, Byrne HM, Kevrekidis P (2023) Spatio-temporal modelling of phenotypic heterogeneity in tumour tissues and its impact on radiotherapy treatment. J Theor Biol 556:111248 36150537 10.1016/j.jtbi.2022.111248

[CR15] Chauviere A, Preziosi L, Byrne H (2010) A model of cell migration within the extracellular matrix based on a phenotypic switching mechanism. Math Med and Biol A J of the IMA 27(3):255–281 10.1093/imammb/dqp021 19942606

[CR16] Chow A, Perica K, Klebanoff CA et al (2022) Clinical implications of T cell exhaustion for cancer immunotherapy. Nat Rev Clin Oncol 19(12):775–790 36216928 10.1038/s41571-022-00689-z PMC10984554

[CR17] Cornell SJ, Suprunenko YF, Finkelshtein D et al (2019) A unified framework for analysis of individual-based models in ecology and beyond. Nat Commun 10(1):4716 31624268 10.1038/s41467-019-12172-y PMC6797757

[CR18] Crossley RM, Maini PK, Lorenzi T et al (2023) Traveling waves in a coarse-grained model of volume-filling cell invasion: simulations and comparisons. Stud Appl Math 151(4):1471–1497

[CR19] Crossley RM, Johnson S, Tsingos E et al (2024a) Modeling the extracellular matrix in cell migration and morphogenesis: a guide for the curious biologist. Front in Cell and Dev Biol 12:1354132 38495620 10.3389/fcell.2024.1354132 PMC10940354

[CR20] Crossley RM, Painter KJ, Lorenzi T, et al (2024b) Phenotypic switching mechanisms determine the structure of cell migration into extracellular matrix under the ‘go-or-grow’ hypothesis. Math Biosci pp 109240 10.1016/j.mbs.2024.109240 38906525

[CR21] Excoffier L, Foll M, Petit RJ (2009) Genetic consequences of range expansions. Annu Rev Ecol Evol Syst 40(1):481–501

[CR22] Falcó C, Crossley RM, Baker RE (2024) Travelling waves in a minimal go-or-grow model of cell invasion. Appl Math Lett 158:109209

[CR23] Hatzikirou H, Basanta D, Simon M et al (2012) ‘Go or grow’: the key to the emergence of invasion in tumour progression? Math Med and Biol A J of the IMA 29(1):49–65 10.1093/imammb/dqq011 20610469

[CR24] Jabłońska-Trypuć A, Matejczyk M, Rosochacki S (2016) Matrix metalloproteinases (MMPs), the main extracellular matrix (ECM) enzymes in collagen degradation, as a target for anticancer drugs. J Enzyme Inhib Med Chem 31(sup1):177–183 27028474 10.3109/14756366.2016.1161620

[CR25] Jiang Y, Li Y, Zhu B (2015) T-cell exhaustion in the tumor microenvironment. Cell Death & Dis 6(6):e1792–e1792 10.1038/cddis.2015.162 PMC4669840 26086965

[CR26] Klopfstein S, Currat M, Excoffier L (2006) The fate of mutations surfing on the wave of a range expansion. Mol Biol Evol 23(3):482–490 16280540 10.1093/molbev/msj057

[CR27] Koren B (1993) A robust upwind discretization method for advection, diffusion and source terms, vol 45. Centrum Wiskunde & Informatica, Amsterdam

[CR28] Lorenzi T, Painter KJ (2022) Trade-offs between chemotaxis and proliferation shape the phenotypic structuring of invading waves. Int J Non-Linear Mech 139:103885

[CR29] Lorenzi T, Macfarlane F, Villa C (2020) Discrete and continuum models for the evolutionary and spatial dynamics of cancer: a very short introduction through two case studies. Trends in Biomath: Model Cells, Flows, Epidemics, and the Environ pp 359–380

[CR30] Lorenzi T, Perthame B, Ruan X (2022) Invasion fronts and adaptive dynamics in a model for the growth of cell populations with heterogeneous mobility. Eur J Appl Math 33(4):766–783

[CR31] Macfarlane FR, Lorenzi T, Painter KJ (2022) The impact of phenotypic heterogeneity on chemotactic self-organisation. Bull Math Biol 84(12):143 36319913 10.1007/s11538-022-01099-z PMC9626439

[CR32] Macfarlane FR, Ruan X, Lorenzi T (2022) Individual-based and continuum models of phenotypically heterogeneous growing cell populations. AIMS Bioeng 9(1):68–92

[CR33] Marculis NG, Lewis MA (2020) Inside dynamics of integrodifference equations with mutations. Bull Math Biol 82(1):7 31932985 10.1007/s11538-019-00683-0

[CR34] Marculis NG, Evenden ML, Lewis MA (2020) Modeling the dispersal-reproduction trade-off in an expanding population. Theor Popul Biol 134:147–159 10.1016/j.tpb.2020.03.003 32209326

[CR35] Marculis NG, Garnier J, Lui R et al (2020) Inside dynamics for stage-structured integrodifference equations. J Math Biol 80:157–187 31076846 10.1007/s00285-019-01378-9

[CR36] Merino-Casallo F, Gomez-Benito MJ, Hervas-Raluy S et al (2022) Unravelling cell migration: defining movement from the cell surface. Cell Adhesion & Migr 16(1):25–64 10.1080/19336918.2022.2055520 PMC9067518 35499121

[CR37] Miller BC, Sen DR, Al Abosy R et al (2019) Subsets of exhausted CD8+ T cells differentially mediate tumor control and respond to checkpoint blockade. Nat Immunol 20(3):326–336 30778252 10.1038/s41590-019-0312-6 PMC6673650

[CR38] Murray PJ, Edwards CM, Tindall MJ et al (2009) From a discrete to a continuum model of cell dynamics in one dimension. Phy Rev E-Stat Nonlinear and Soft Matter Phy 80(3):031912 10.1103/PhysRevE.80.031912 19905151

[CR39] Murray PJ, Walter A, Fletcher AG et al (2011) Comparing a discrete and continuum model of the intestinal crypt. Phys Biol 8(2):026011 21411869 10.1088/1478-3975/8/2/026011 PMC3164594

[CR40] Nagase H, Woessner JF (1999) Matrix metalloproteinases. J Biol Chem 274(31):21491–21494 10419448 10.1074/jbc.274.31.21491

[CR41] Nagase H, Visse R, Murphy G (2006) Structure and function of matrix metalloproteinases and TIMPs. Cardiovasc Res 69(3):562–573 16405877 10.1016/j.cardiores.2005.12.002

[CR42] Reina-Campos M, Goldrath AW (2019) Antitumour T cells stand the test of time. Nature 576(7787):392–394 31844255 10.1038/d41586-019-03731-w

[CR43] Roques L, Garnier J, Hamel F et al (2012) Allee effect promotes diversity in traveling waves of colonization. Proc Natl Acad Sci 109(23):8828–8833 22611189 10.1073/pnas.1201695109 PMC3384151

[CR44] Schaller G, Meyer-Hermann M (2006) Continuum versus discrete model: a comparison for multicellular tumour spheroids. Philos Trans of the Royal Soc A Math Phys and Eng Sci 364(1843):1443–1464 10.1098/rsta.2006.1780 16766354

[CR45] Schreiber RD, Old LJ, Smyth MJ (2011) Cancer immunoediting: integrating immunity’s roles in cancer suppression and promotion. Sci 331(6024):1565–1570 10.1126/science.1203486 21436444

[CR46] Sprent J, Surh CD (2011) Normal T cell homeostasis: the conversion of naive cells into memory-phenotype cells. Nat Immunol 12(6):478–484 21739670 10.1038/ni.2018 PMC3434123

[CR47] Stepien TL, Rutter EM, Kuang Y (2018) Traveling waves of a go-or-grow model of glioma growth. SIAM J Appl Math 78(3):1778–1801

[CR48] Strobl MA, Krause AL, Damaghi M et al (2020) Mix and match: phenotypic coexistence as a key facilitator of cancer invasion. Bull Math Biol 82:1–26 10.1007/s11538-019-00675-0 PMC6968991 31953602

[CR49] Suresh S (2007) Biomechanics and biophysics of cancer cells. Acta Biomater 3(4):413–438 17540628 10.1016/j.actbio.2007.04.002 PMC2917191

[CR50] Trepat X, Chen Z, Jacobson K (2012) Cell migration. Compr Physiol 2(4):2369 23720251 10.1002/cphy.c110012 PMC4457291

[CR51] Van Liedekerke P, Palm M, Jagiella N et al (2015) Simulating tissue mechanics with agent-based models: concepts, perspectives and some novel results. Comput Part Mech 2:401–444

[CR52] Villa C, Maini PK, Browning AP, et al (2024) Reducing phenotype-structured pde models of cancer evolution to systems of odes: a generalised moment dynamics approach. arXiv preprint arXiv:2406.01505 10.1007/s00285-025-02246-5 PMC12304065 40719870

[CR53] Visse R, Nagase H (2003) Matrix metalloproteinases and tissue inhibitors of metalloproteinases: structure, function, and biochemistry. Circ Res 92(8):827–839 12730128 10.1161/01.RES.0000070112.80711.3D

[CR54] Wang Z, Butner JD, Kerketta R, et al (2015) Simulating cancer growth with multiscale agent-based modeling. In: Seminars in Cancer Biology, Elsevier, pp 70–78 10.1016/j.semcancer.2014.04.001 PMC4216775 24793698

[CR55] Weninger W, Crowley MA, Manjunath N et al (2001) Migratory properties of naive, effector, and memory CD8+ T cells. J Exp Med 194(7):953–966 11581317 10.1084/jem.194.7.953 PMC2193483

[CR56] West J, Robertson-Tessi M, Anderson AR (2023) Agent-based methods facilitate integrative science in cancer. Trends Cell Biol 33(4):300–311 36404257 10.1016/j.tcb.2022.10.006 PMC10918696

[CR57] Wherry EJ, Ahmed R (2004) Memory CD8 T-cell differentiation during viral infection. J Virol 78(11):5535–5545 15140950 10.1128/JVI.78.11.5535-5545.2004 PMC415833

[CR58] Winkler J, Abisoye-Ogunniyan A, Metcalf KJ et al (2020) Concepts of extracellular matrix remodelling in tumour progression and metastasis. Nat Commun 11(1):5120 33037194 10.1038/s41467-020-18794-x PMC7547708

[CR59] Worbs T, Förster R (2009) T cell migration dynamics within lymph nodes during steady state: an overview of extracellular and intracellular factors influencing the basal intranodal T cell motility. Vis Immun 71–105 10.1007/978-3-540-93864-4_4 19521682

[CR60] Yi JS, Cox MA, Zajac AJ (2010) T-cell exhaustion: characteristics, causes and conversion. Immunol 129(4):474–481 10.1111/j.1365-2567.2010.03255.x PMC2842494 20201977

