# Supplementary material for: Modelling the Impact of Phenotypic Heterogeneity on Cell Migration: A Continuum Framework Derived from Individual-Based Principles
Source: Bull Math Biol. 2025 Aug 8;87(9):123. doi: 10.1007/s11538-025-01502-5 (PMC12334544; doi:10.1007/s11538-025-01502-5)
Supplement: Supplementary file 1 — (pdf 761 KB) [file 11538_2025_1502_MOESM1_ESM.pdf]

Supplementary information for

Modelling the impact of phenotypic heterogeneity on cell migration: a continuum framework derived from individual-based principles

Rebecca M. Crossley*[1], Philip K. Maini†[1], and Ruth E. Baker‡[1]

[1]Mathematical Institute, University of Oxford, Oxford, United Kingdom

*Email: crossley@maths.ox.ac.uk
†Email: maini@maths.ox.ac.uk
‡Email: baker@maths.ox.ac.uk

S1 Formal derivation of the continuum Eqs. (5) and (12)

In this supplementary information, we present the full formal derivation of the continuum model (5) and (12) from the individual-based model described in Sec. 2.

When the dynamics of the cells and the local environment are described by the rules outlined in Sec. 2, then the master Eq. (1) is given by

$$\begin{aligned}
&\Delta_t \frac{\partial}{\partial t} p(\mathbf{n}, \mathbf{e}, t_h) + O(\Delta_t^2) \\
&= \sum_{i=1}^{N_x+1} \sum_{j=1}^{N_y} \mu_-(j+1, N_i, e_i) \left\{ (n_i^{j+1} + 1) p(U_{i,j}^{\mathrm{p}} \mathbf{n}, \mathbf{e}, t_h) - n_i^{j+1} p(\mathbf{n}, \mathbf{e}, t_h) \right\} \\
&+ \sum_{i=1}^{N_x+1} \sum_{j=2}^{N_y+1} \mu_+(j-1, N_i, e_i) \left\{ (n_i^{j-1} + 1) p(D_{i,j}^{\mathrm{p}} \mathbf{n}, \mathbf{e}, t_h) - n_i^{j-1} p(\mathbf{n}, \mathbf{e}, t_h) \right\} \\
&+ \sum_{i=1}^{N_x} \sum_{j=1}^{N_y+1} \beta_-(j, N_i, e_i) \left\{ (n_{i+1}^{j} + 1) p(R_{i,j}^{\mathrm{m}} \mathbf{n}, \mathbf{e}, t_h) - n_{i+1}^{j} p(\mathbf{n}, \mathbf{e}, t_h) \right\} \\
&+ \sum_{i=2}^{N_x+1} \sum_{j=1}^{N_y+1} \beta_+(j, N_i, e_i) \left\{ (n_{i-1}^{j} + 1) p(L_{i,j}^{\mathrm{m}} \mathbf{n}, \mathbf{e}, t_h) - n_{i-1}^{j} p(\mathbf{n}, \mathbf{e}, t_h) \right\} \\
&+ \sum_{i=1}^{N_x+1} \sum_{j=1}^{N_y+1} \left\{ \gamma(j, N_i - 1, e_i)(n_i^j - 1) p(G_{i,j} \mathbf{n}, \mathbf{e}, t_h) - \gamma(j, N_i, e_i) n_i^j p(\mathbf{n}, \mathbf{e}, t_h) \right\} \\
&+ \sum_{i=1}^{N_x+1} \sum_{j=1}^{N_y+1} \lambda(j, n_i^j) \left\{ (e_i + 1) p(\mathbf{n}, H_i \mathbf{e}, t_h) - e_i p(\mathbf{n}, \mathbf{e}, t_h) \right\}, \qquad \text{(S1)}
\end{aligned}$$

S1.1 Equation for cell density

As is standard in the literature, we define the ensemble average for the function, f, of the number of cells at position i in state j and the number of elements of the local environment in lattice site i in the following way:

$$\langle f(n_i^j, e_i) \rangle = \sum_{\mathbf{n}} \sum_{\mathbf{e}} f(n_i^j, e_i) p(\mathbf{n}, \mathbf{e}, t_h). \qquad \text{(S2)}$$

Now, returning to Eq. (S1), we first take moments and multiply by n_s^q, where $s = 1, \ldots, N_x+1$ and $q = 1, \ldots, N_y + 1$ and take the sum over the vectors $\mathbf{n}$ and $\mathbf{e}$. The final term in Eq. (S1) corresponds to the evolution of the number of elements of the local environment, and does not contribute to the cell dynamics. Then, to begin with, we consider only the first remaining term on the right-hand side, which we denote by I, in the following way:

$$I = \sum_{\mathbf{n}} \sum_{\mathbf{e}} n_s^q \sum_{i=1}^{N_x+1} \sum_{j=1}^{N_y} \mu_-(j+1, N_i, e_i) \left\{ (n_i^{j+1} + 1) p(U_j^{\mathrm{p}} \mathbf{n}, \mathbf{e}, t_h) - n_i^{j+1} p(\mathbf{n}, \mathbf{e}, t_h) \right\}.$$

To continue, we must now consider the following cases in turn:

- I_1: $i \neq s$, $j \neq q, q-1$;
- I_2: $i \neq s$ and $j = q$;
- I_3: $i \neq s$ and $j = q-1$;
- I_4: $i = s$ and $j \neq q, q-1$;
- I_5: $i = s$ and $j = q$;
- I_6: $i = s$ and $j = q-1$.

We begin with the case I_1 where $i \neq s$ and $j \neq q, q-1$. In this scenario, we find

$$\begin{aligned}
I_1 &= \sum_{\mathbf{n}} \sum_{\mathbf{e}} n_s^q \sum_{\substack{i=1,\\ i\neq s}}^{N_x+1} \sum_{\substack{j=1,\\ j\neq q,q-1}}^{N_y} \mu_-(j+1, N_i, e_i) \left\{ (n_i^{j+1}+1) p(U_j^{\mathrm{p}}\mathbf{n}, \mathbf{e}, t_h) - n_i^{j+1} p(\mathbf{n}, \mathbf{e}, t_h) \right\} \\
&= \sum_{\mathbf{n}} \sum_{\mathbf{e}} n_s^q \sum_{\substack{i=1,\\ i\neq s}}^{N_x+1} \sum_{\substack{j=1,\\ j\neq q,q-1}}^{N_y} \mu_-(j+1, N_i, e_i) \times \\
&\qquad \left\{ (n_i^{j+1}+1) p([n_i^1, \ldots, n_i^j - 1, n_i^{j+1}+1, \ldots, n_i^{N_y+1}], \mathbf{e}, t_h) - n_i^{j+1} p(\mathbf{n}, \mathbf{e}, t_h) \right\}.
\end{aligned}$$

Under the change of variables $\bar{n}_i^j = n_i^j - 1$ and $\bar{n}_i^{j+1} = n_i^{j+1} + 1$ in the first term, we have that

$$\begin{aligned}
I_1 &= \sum_{\mathbf{n}} \sum_{\mathbf{e}} n_s^q \sum_{\substack{i=1,\\ i\neq s}}^{N_x+1} \sum_{\substack{j=1,\\ j\neq q,q-1}}^{N_y} \mu_-(j+1, N_i, e_i) \times \\
&\qquad \left\{ \bar{n}_i^{j+1} p([n_i^1, \ldots, \bar{n}_i^j, \bar{n}_i^{j+1}, \ldots, n_i^{N_y+1}], \mathbf{e}, t_h) - n_i^{j+1} p(\mathbf{n}, \mathbf{e}, t_h) \right\} \\
&= \sum_{\mathbf{n}} \sum_{\mathbf{e}} n_s^q \sum_{\substack{i=1,\\ i\neq s}}^{N_x+1} \sum_{\substack{j=1,\\ j\neq q,q-1}}^{N_y} \mu_-(j+1, N_i, e_i) \left\{ n_i^{j+1} p(\mathbf{n}, \mathbf{e}, t_h) - n_i^{j+1} p(\mathbf{n}, \mathbf{e}, t_h) \right\} \\
&= 0,
\end{aligned}$$

where we can arbitrarily drop the bar after the change of variables.

Next, we consider the case when $i \neq s$ and $j = q$, and apply the same change of variables

argument (with $\bar{n}_i^q = n_i^q - 1$ and $\bar{n}_i^{q+1} = n_i^{q+1} + 1$) so that

$$\begin{aligned}
I_2 &= \sum_{\mathbf{n}} \sum_{\mathbf{e}} n_s^q \sum_{\substack{i=1,\\ i\neq s}}^{N_x+1} \mu_-(q+1, N_i, e_i)\times \\
&\qquad \Big\{(n_i^{q+1}+1)p([n_i^1,\dots,n_i^q-1,n_i^{q+1}+1,\dots,n_i^{N_y+1}],\mathbf{e},t_h) - n_i^{q+1}p(\mathbf{n},\mathbf{e},t_h)\Big\} \\
&= \sum_{\mathbf{n}} \sum_{\mathbf{e}} n_s^q \sum_{\substack{i=1,\\ i\neq s}}^{N_x+1} \mu_-(q+1, N_i, e_i)\times \\
&\qquad \Big\{\bar{n}_i^{q+1}p([n_i^1,\dots,\bar{n}_i^q,\bar{n}_i^{q+1},\dots,n_i^{N_y+1}],\mathbf{e},t_h) - n_i^{q+1}p(\mathbf{n},\mathbf{e},t_h)\Big\} \\
&= \sum_{\mathbf{n}} \sum_{\mathbf{e}} n_s^q \sum_{\substack{i=1,\\ i\neq s}}^{N_x+1} \mu_-(q+1, N_i, e_i)\Big\{n_i^{q+1}p(\mathbf{n},\mathbf{e},t_h) - n_i^{q+1}p(\mathbf{n},\mathbf{e},t_h)\Big\} \\
&= 0.
\end{aligned}$$

Finally we consider the last case for $i \neq s$, namely, where $j = q-1$. Once again, by changing the variables using $\bar{n}_i^{q-1} = n_i^{q-1} - 1$ and $\bar{n}_i^q = n_i^q + 1$, we have

$$\begin{aligned}
I_3 &= \sum_{\mathbf{n}} \sum_{\mathbf{e}} n_s^q \sum_{\substack{i=1,\\ i\neq s}}^{N_x+1} \mu_-(q, N_i, e_i)\times \\
&\qquad \Big\{(n_i^{q}+1)p([n_i^1,\dots,n_i^{q-1}-1,n_i^{q}+1,\dots,n_i^{N_y+1}],\mathbf{e},t_h) - n_i^{q}p(\mathbf{n},\mathbf{e},t_h)\Big\} \\
&= \sum_{\mathbf{n}} \sum_{\mathbf{e}} n_s^q \sum_{\substack{i=1,\\ i\neq s}}^{N_x+1} \mu_-(q, N_i, e_i)\times \\
&\qquad \Big\{\bar{n}_i^{q}p([n_i^1,\dots,\bar{n}_i^{q-1},\bar{n}_i^{q},\dots,n_i^{N_y+1}],\mathbf{e},t_h) - n_i^{q}p(\mathbf{n},\mathbf{e},t_h)\Big\} \\
&= \sum_{\mathbf{n}} \sum_{\mathbf{e}} n_s^q \sum_{\substack{i=1,\\ i\neq s}}^{N_x+1} \mu_-(q, N_i, e_i)\{n_i^{q}p(\mathbf{n},\mathbf{e},t_h) - n_i^{q}p(\mathbf{n},\mathbf{e},t_h)\} \\
&= 0.
\end{aligned}$$

As such, we can see that there are no contributions arising from the terms for which $i \neq s$.

Now we can consider cases where $i = s$. First off, we consider $j \neq q, q-1$, and apply the

change of variables $\bar{n}_s^j = n_s^j - 1$ and $\bar{n}_s^{j+1} = n_s^{j+1} + 1$:

$$
\begin{aligned}
I_4 &= \sum_{\mathbf{n}} \sum_{\mathbf{e}} n_s^q \sum_{\substack{j=1, \\ j \neq q, q-1}}^{N_y} \mu_-(j+1, N_s, e_s) \times \\
&\qquad \left\{ (n_s^{j+1} + 1) p([n_s^1, \ldots, n_s^j - 1, n_s^{j+1} + 1, \ldots, n_s^{N_y+1}], \mathbf{e}, t_h) - n_s^{j+1} p(\mathbf{n}, \mathbf{e}, t_h) \right\} \\
&= \sum_{\mathbf{n}} \sum_{\mathbf{e}} n_s^q \sum_{\substack{j=1, \\ j \neq q, q-1}}^{N_y} \mu_-(j+1, N_s, e_s) \times \\
&\qquad \left\{ \bar{n}_s^{j+1} p([n_s^1, \ldots, \bar{n}_s^j, \bar{n}_s^{j+1}, \ldots, n_s^{N_y+1}], \mathbf{e}, t_h) - n_s^{j+1} p(\mathbf{n}, \mathbf{e}, t_h) \right\} \\
&= \sum_{\mathbf{n}} \sum_{\mathbf{e}} n_s^q \sum_{\substack{j=1, \\ j \neq q, q-1}}^{N_y} \mu_-(j+1, N_s, e_s) \left\{ n_s^{j+1} p(\mathbf{n}, \mathbf{e}, t_h) - n_s^{j+1} p(\mathbf{n}, \mathbf{e}, t_h) \right\} \\
&= 0.
\end{aligned}
$$

Then, considering $i = s$ and $j = q$, we see that

$$
\begin{aligned}
I_5 = \sum_{\mathbf{n}} \sum_{\mathbf{e}} & n_s^q \mu_-(q+1, N_s, e_s) \times \\
&\left\{ (n_s^{q+1} + 1) p([n_s^1, \ldots, n_s^q - 1, n_s^{q+1} + 1, \ldots, n_s^{N_y+1}], \mathbf{e}, t_h) - n_s^{q+1} p(\mathbf{n}, \mathbf{e}, t_h) \right\}.
\end{aligned}
$$

Now we want to separate the terms and apply the change of variables $\bar{n}_s^q = n_s^q - 1$ and $\bar{n}_s^{q+1} = n_s^{q+1} + 1$ in the first term to see

$$
\begin{aligned}
I_5 &= \sum_{\mathbf{n}} \sum_{\mathbf{e}} \mu_-(q+1, N_s, e_s) \times \\
&\qquad \left\{ n_s^q (n_s^{q+1} + 1) p([n_s^1, \ldots, n_s^q - 1, n_s^{q+1} + 1, \ldots, n_s^{N_y+1}], \mathbf{e}, t_h) - n_s^q n_s^{q+1} p(\mathbf{n}, \mathbf{e}, t_h) \right\} \\
&= \sum_{\mathbf{n}} \sum_{\mathbf{e}} \mu_-(q+1, N_s, e_s) \times \\
&\qquad \left\{ (\bar{n}_s^q + 1) \bar{n}_s^{q+1} p([n_s^1, \ldots, \bar{n}_s^q, \bar{n}_s^{q+1}, \ldots, n_s^{N_y+1}], \mathbf{e}, t_h) - n_s^q n_s^{q+1} p(\mathbf{n}, \mathbf{e}, t_h) \right\} \\
&= \sum_{\mathbf{n}} \sum_{\mathbf{e}} \mu_-(q+1, N_s, e_s) \left\{ n_s^{q+1} (n_s^q + 1) p(\mathbf{n}, \mathbf{e}, t_h) - n_s^q n_s^{q+1} p(\mathbf{n}, \mathbf{e}, t_h) \right\} \\
&= \sum_{\mathbf{n}} \sum_{\mathbf{e}} \mu_-(q+1, N_s, e_s) n_s^{q+1} p(\mathbf{n}, \mathbf{e}, t_h) \\
&= \langle \mu_-(q+1, N_s, e_s) n_s^{q+1} \rangle.
\end{aligned}
$$

Finally, considering the case when $i = s$ and $j = q - 1$ we have

$$I_6 = \sum_{\mathbf{n}} \sum_{\mathbf{e}} n_s^q \mu_-(q, N_s, e_s) \times \left\{ (n_s^q + 1) p([n_s^1, \ldots, n_s^{q-1} - 1, n_s^q + 1, \ldots, n_s^{N_y+1}], \mathbf{e}, t_h) - n_s^q p(\mathbf{n}, \mathbf{e}, t_h) \right\}.$$

Separating out the two terms and applying the change of variables $\bar{n}_s^q = n_s^q + 1$ and $\bar{n}_s^{q-1} = n_s^{q-1} - 1$ and then dropping the bars in the first term, we see

$$\begin{aligned} I_6 &= \sum_{\mathbf{n}} \sum_{\mathbf{e}} \mu_-(q, N_s, e_s) \times \left\{ n_s^q (n_s^q + 1) p([n_s^1, \ldots, n_s^{q-1} - 1, n_s^q + 1, \ldots, n_s^{N_y+1}], \mathbf{e}, t_h) - n_s^q n_s^q p(\mathbf{n}, \mathbf{e}, t_h) \right\} \\ &= \sum_{\mathbf{n}} \sum_{\mathbf{e}} \mu_-(q, N_s, e_s) \times \left\{ (\bar{n}_s^q - 1) \bar{n}_s^q p([n_s^1, \ldots, \bar{n}_s^{q-1}, \bar{n}_s^q, \ldots, n_s^{N_y+1}], \mathbf{e}, t_h) - n_s^q n_s^q p(\mathbf{n}, \mathbf{e}, t_h) \right\} \\ &= \sum_{\mathbf{n}} \sum_{\mathbf{e}} \mu_-(q, N_s, e_s) \left\{ n_s^q (n_s^q - 1) p(\mathbf{n}, \mathbf{e}, t_h) - (n_s^q)^2 p(\mathbf{n}, \mathbf{e}, t_h) \right\} \\ &= -\sum_{\mathbf{n}} \sum_{\mathbf{e}} \mu_-(q, N_s, e_s) n_s^q p(\mathbf{n}, \mathbf{e}, t_h) \\ &= -\langle \mu_-(q, N_s, e_s) n_s^q \rangle. \end{aligned}$$

Putting these all back together, we find that

$$\begin{aligned} I &= I_1 + I_2 + I_3 + I_4 + I_5 + I_6 \\ &= \langle \mu_-(q+1, N_s, e_s) n_s^{q+1} \rangle - \langle \mu_-(q, N_s, e_s) n_s^q \rangle. \end{aligned}$$

Now we repeat this process for the second term in Eq. (S1), which we call J:

$$\begin{aligned} J &= \sum_{\mathbf{n}} \sum_{\mathbf{e}} n_s^q \sum_{i=1}^{N_x+1} \sum_{j=2}^{N_y+1} \mu_+(j-1, N_i, e_i) \left\{ (n_i^{j-1} + 1) p(D_{i,j}^{\mathrm{p}} \mathbf{n}, \mathbf{e}, t_h) - n_i^{j-1} p(\mathbf{n}, \mathbf{e}, t_h) \right\}, \\ &= J_1 + J_2 + J_3 + J_4 + J_5 + J_6 \end{aligned}$$

and consider the six cases: $J_1, \ldots, J_6$ (defined in the same way as before) where we have $i = s, i \neq s, j = q, j = q + 1$, and $j \neq q, q + 1$. Starting with $i \neq s$ and $j \neq q, q + 1$ we see, using the

change of variables $\bar{n}_i^j = n_i^j - 1$ and $\bar{n}_i^{j-1} = n_i^{j-1} + 1$, that we have

$$
\begin{aligned}
J_1 &= \sum_{\mathbf{n}} \sum_{\mathbf{e}} n_s^q \sum_{\substack{i=1,\\ i\neq s}}^{N_x+1} \sum_{\substack{j=2,\\ j\neq q,q-1}}^{N_y+1} \mu_+(j-1, N_i, e_i) \left\{ (n_i^{j-1}+1) p(D_{i,j}^{\mathrm{p}}\mathbf{n}, \mathbf{e}, t_h) - n_i^{j-1} p(\mathbf{n}, \mathbf{e}, t_h) \right\} \\
&= \sum_{\mathbf{n}} \sum_{\mathbf{e}} n_s^q \sum_{\substack{i=1,\\ i\neq s}}^{N_x+1} \sum_{\substack{j=2,\\ j\neq q,q-1}}^{N_y+1} \mu_+(j-1, N_i, e_i) \times \\
&\qquad \left\{ (n_i^{j-1}+1) p([n_i^1, \ldots, n_i^{j-1}+1, n_i^j - 1, \ldots, n_i^{N_y+1}], \mathbf{e}, t_h) - n_i^{j-1} p(\mathbf{n}, \mathbf{e}, t_h) \right\} \\
&= \sum_{\mathbf{n}} \sum_{\mathbf{e}} n_s^q \sum_{\substack{i=1,\\ i\neq s}}^{N_x+1} \sum_{\substack{j=2,\\ j\neq q,q-1}}^{N_y+1} \mu_+(j-1, N_i, e_i) \times \\
&\qquad \left\{ \bar{n}_i^{j-1} p([n_i^1, \ldots, \bar{n}_i^{j-1}, \bar{n}_i^j, \ldots, n_i^{N_y+1}], \mathbf{e}, t_h) - n_i^{j-1} p(\mathbf{n}, \mathbf{e}, t_h) \right\} \\
&= \sum_{\mathbf{n}} \sum_{\mathbf{e}} n_s^q \sum_{\substack{i=1,\\ i\neq s}}^{N_x+1} \sum_{\substack{j=2,\\ j\neq q,q-1}}^{N_y+1} \mu_+(j-1, N_i, e_i) \left\{ n_i^{j-1} p(\mathbf{n}, \mathbf{e}, t_h) - n_i^{j-1} p(\mathbf{n}, \mathbf{e}, t_h) \right\} \\
&= 0.
\end{aligned}
$$

Next, we consider the case when $i \neq s$ and $j = q$, and apply the same change of variables argument (with $\bar{n}_i^q = n_i^q - 1$ and $\bar{n}_i^{q-1} = n_i^{q-1} + 1$) to the first term:

$$
\begin{aligned}
J_2 &= \sum_{\mathbf{n}} \sum_{\mathbf{e}} n_s^q \sum_{\substack{i=1,\\ i\neq s}}^{N_x+1} \mu_+(q-1, N_i, e_i) \times \\
&\qquad \left\{ (n_i^{q-1}+1) p([n_i^1, \ldots, n_i^{q-1}+1, n_i^q - 1, \ldots, n_i^{N_y+1}], \mathbf{e}, t_h) - n_i^{q-1} p(\mathbf{n}, \mathbf{e}, t_h) \right\} \\
&= \sum_{\mathbf{n}} \sum_{\mathbf{e}} n_s^q \sum_{\substack{i=1,\\ i\neq s}}^{N_x+1} \mu_+(q-1, N_i, e_i) \times \\
&\qquad \left\{ \bar{n}_i^{q-1} p([n_i^1, \ldots, \bar{n}_i^{q-1}, \bar{n}_i^q, \ldots, n_i^{N_y+1}], \mathbf{e}, t_h) - n_i^{q-1} p(\mathbf{n}, \mathbf{e}, t_h) \right\} \\
&= \sum_{\mathbf{n}} \sum_{\mathbf{e}} n_s^q \sum_{\substack{i=1,\\ i\neq s}}^{N_x+1} \mu_+(q-1, N_i, e_i) \left\{ n_i^{q-1} p(\mathbf{n}, \mathbf{e}, t_h) - n_i^{q-1} p(\mathbf{n}, \mathbf{e}, t_h) \right\} \\
&= 0.
\end{aligned}
$$

Finally we consider the last remaining situation when $i \neq s$, where $j = q + 1$. Once again, by

changing the variables using $\bar{n}_i^{q+1} = n_i^{q+1} - 1$ and $\bar{n}_i^q = n_i^q + 1$, we have

$$
\begin{aligned}
J_3 &= \sum_{\mathbf{n}} \sum_{\mathbf{e}} n_s^q \sum_{\substack{i=1, \\ i \neq s}}^{N_x+1} \mu_+(q, N_i, e_i) \times \\
&\qquad \left\{ (n_i^q + 1) p([n_i^1, \ldots, n_i^q + 1, n_i^{q+1} - 1, \ldots, n_i^{N_y+1}], \mathbf{e}, t_h) - n_i^q p(\mathbf{n}, \mathbf{e}, t_h) \right\} \\
&= \sum_{\mathbf{n}} \sum_{\mathbf{e}} n_s^q \sum_{\substack{i=1, \\ i \neq s}}^{N_x+1} \mu_+(q, N_i, e_i) \left\{ \bar{n}_i^q p([n_i^1, \ldots, \bar{n}_i^q, \bar{n}_i^{q+1}, \ldots, n_i^{N_y+1}], \mathbf{e}, t_h) - n_i^q p(\mathbf{n}, \mathbf{e}, t_h) \right\} \\
&= \sum_{\mathbf{n}} \sum_{\mathbf{e}} n_s^q \sum_{\substack{i=1, \\ i \neq s}}^{N_x+1} \mu_+(q, N_i, e_i) \left\{ n_i^q p(\mathbf{n}, \mathbf{e}, t_h) - n_i^q p(\mathbf{n}, \mathbf{e}, t_h) \right\} \\
&= 0.
\end{aligned}
$$

As such, we can see that there are no contributions arising from the terms when $i \neq s$.

Now we can consider cases where $i = s$. First, we consider $j \neq q, q+1$, and apply the change of variables $\bar{n}_s^j = n_s^j - 1$ and $\bar{n}_s^{j-1} = n_s^{j-1} + 1$:

$$
\begin{aligned}
J_4 &= \sum_{\mathbf{n}} \sum_{\mathbf{e}} n_s^q \sum_{\substack{j=2, \\ j \neq q, q-1}}^{N_y+1} \mu_+(j-1, N_s, e_s) \times \\
&\qquad \left\{ (n_s^{j-1} + 1) p([n_s^1, \ldots, n_s^{j-1} + 1, n_s^j - 1, \ldots, n_s^{N_y+1}], \mathbf{e}, t_h) - n_s^{j-1} p(\mathbf{n}, \mathbf{e}, t_h) \right\} \\
&= \sum_{\mathbf{n}} \sum_{\mathbf{e}} n_s^q \sum_{\substack{j=2, \\ j \neq q, q-1}}^{N_y+1} \mu_+(j-1, N_s, e_s) \times \\
&\qquad \left\{ \bar{n}_s^{j-1} p([n_s^1, \ldots, \bar{n}_s^{j-1}, \bar{n}_s^j, \ldots, n_s^{N_y+1}], \mathbf{e}, t_h) - n_s^{j-1} p(\mathbf{n}, \mathbf{e}, t_h) \right\} \\
&= \sum_{\mathbf{n}} \sum_{\mathbf{e}} n_s^q \sum_{\substack{j=2, \\ j \neq q, q-1}}^{N_y+1} \mu_+(j-1, N_s, e_s) \left\{ n_s^{j-1} p(\mathbf{n}, \mathbf{e}, t_h) - n_s^{j-1} p(\mathbf{n}, \mathbf{e}, t_h) \right\} \\
&= 0.
\end{aligned}
$$

Then, considering the case when $i = s$ and $j = q$, we see that applying the change of variables

$\bar{n}_s^q = n_s^q - 1$ and $\bar{n}_s^{q-1} = n_s^{q-1} + 1$ in the first term, we have

$$
\begin{aligned}
J_5 = &\sum_{\mathbf{n}} \sum_{\mathbf{e}} n_s^q \mu_+(q-1, N_s, e_s) \times \\
&\qquad \Big\{ (n_s^{q-1}+1) p([n_s^1, \dots, n_s^{q-1}+1, n_s^q - 1, \dots, n_s^{N_y+1}], \mathbf{e}, t_h) - n_s^{q-1} p(\mathbf{n}, \mathbf{e}, t_h) \Big\} \\
= &\sum_{\mathbf{n}} \sum_{\mathbf{e}} \mu_+(q-1, N_s, e_s) \times \\
&\qquad \Big\{ n_s^q (n_s^{q-1}+1) p([n_s^1, \dots, n_s^{q-1}+1, n_s^q - 1, \dots, n_s^{N_y+1}], \mathbf{e}, t_h) - n_s^q n_s^{q-1} p(\mathbf{n}, \mathbf{e}, t_h) \Big\} \\
= &\sum_{\mathbf{n}} \sum_{\mathbf{e}} \mu_+(q-1, N_s, e_s) \times \\
&\qquad \Big\{ (\bar{n}_s^q + 1) \bar{n}_s^{q-1} p([n_s^1, \dots, \bar{n}_s^{q-1}, \bar{n}_s^q, \dots, n_s^{N_y+1}], \mathbf{e}, t_h) - n_s^q n_s^{q-1} p(\mathbf{n}, \mathbf{e}, t_h) \Big\} \\
= &\sum_{\mathbf{n}} \sum_{\mathbf{e}} \mu_+(q-1, N_s, e_s) \left\{ n_s^{q-1} (n_s^q + 1) p(\mathbf{n}, \mathbf{e}, t_h) - n_s^q n_s^{q-1} p(\mathbf{n}, \mathbf{e}, t_h) \right\} \\
= &\sum_{\mathbf{n}} \sum_{\mathbf{e}} \mu_+(q-1, N_s, e_s) n_s^{q-1} p(\mathbf{n}, \mathbf{e}, t_h) \\
= &\langle \mu_+(q-1, N_s, e_s) n_s^{q-1} \rangle.
\end{aligned}
$$

Finally, considering the case when $i = s$ and $j = q + 1$ we consider term J_6. Using the change of variables $\bar{n}_s^q = n_s^q + 1$ and $\bar{n}_s^{q+1} = n_s^{q+1} - 1$ and then dropping the bars in the first term, we see that

$$
\begin{aligned}
J_6 = &\sum_{\mathbf{n}} \sum_{\mathbf{e}} n_s^q \mu_+(q, N_s, e_s) \times \\
&\qquad \Big\{ (n_s^q + 1) p([n_s^1, \dots, n_s^q + 1, n_s^{q+1} - 1, \dots, n_s^{N_y+1}], \mathbf{e}, t_h) - n_s^q p(\mathbf{n}, \mathbf{e}, t_h) \Big\} \\
= &\sum_{\mathbf{n}} \sum_{\mathbf{e}} \mu_+(q, N_s, e_s) \times \\
&\qquad \Big\{ n_s^q (n_s^q + 1) p([n_s^1, \dots, n_s^q + 1, n_s^{q+1} - 1, \dots, n_s^{N_y+1}], \mathbf{e}, t_h) - n_s^q n_s^q p(\mathbf{n}, \mathbf{e}, t_h) \Big\} \\
= &\sum_{\mathbf{n}} \sum_{\mathbf{e}} \mu_+(q, N_s, e_s) \times \\
&\qquad \Big\{ (\bar{n}_s^q - 1) \bar{n}_s^q p([n_s^1, \dots, \bar{n}_s^q, \bar{n}_s^{q+1}, \dots, n_s^{N_y+1}], \mathbf{e}, t_h) - n_s^q n_s^q p(\mathbf{n}, \mathbf{e}, t_h) \Big\} \\
= &\sum_{\mathbf{n}} \sum_{\mathbf{e}} \mu_+(q, N_s, e_s) \left\{ n_s^q (n_s^q - 1) p(\mathbf{n}, \mathbf{e}, t_h) - (n_s^q)^2 p(\mathbf{n}, \mathbf{e}, t_h) \right\} \\
= &-\sum_{\mathbf{n}} \sum_{\mathbf{e}} \mu_+(q, N_s, e_s) n_s^q p(\mathbf{n}, \mathbf{e}, t_h) \\
= &-\langle \mu_+(q, N_s, e_s) n_s^q \rangle.
\end{aligned}
$$

Putting these all back together, it is clear that

$$\begin{aligned}J &= J_1 + J_2 + J_3 + J_4 + J_5 + J_6 \\ &= \langle \mu_+(q-1, N_s, e_s) n_s^{q-1} \rangle - \langle \mu_+(q, N_s, e_s) n_s^q \rangle.\end{aligned}$$

Next, we consider the terms modelling movement in physical space in time step Δ_t. Returning to Eq. (S1), we look first at the third term on the right-hand side, which we call K. For this term, we once again consider the cases $j = q, j \neq q, i = s, i = s-1$ and $i \neq s, s-1$ in turn, so

$$\begin{aligned}K &= \sum_{\mathbf{n}} \sum_{\mathbf{e}} n_s^q \sum_{i=1}^{N_x} \sum_{j=1}^{N_y+1} \beta_-(j, N_i, e_i) \left\{ (n_{i+1}^j + 1) p(R_{i,j}^{\mathrm{m}} \mathbf{n}, \mathbf{e}, t_h) - n_{i+1}^j p(\mathbf{n}, \mathbf{e}, t_h) \right\} \\ &= K_1 + K_2 + K_3 + K_4 + K_5 + K_6.\end{aligned}$$

Considering first the case where $j \neq q$ and $i \neq s, s-1$, and applying the change of variables $\bar{n}_i^j = n_i^j - 1$ and $\bar{n}_{i+1}^j = n_{i+1}^j + 1$ and dropping the bars, we have

$$\begin{aligned}K_1 &= \sum_{\mathbf{n}} \sum_{\mathbf{e}} n_s^q \sum_{\substack{i=1,\\ i \neq s, s-1}}^{N_x} \sum_{\substack{j=1,\\ j \neq q}}^{N_y+1} \beta_-(j, N_i, e_i) \times \\ &\qquad \left\{ (n_{i+1}^j + 1) p([n_1^j, \ldots, n_i^j - 1, n_{i+1}^j + 1, \ldots, n_{N_x+1}^j], \mathbf{e}, t_h) - n_{i+1}^j p(\mathbf{n}, \mathbf{e}, t_h) \right\} \\ &= \sum_{\mathbf{n}} \sum_{\mathbf{e}} n_s^q \sum_{\substack{i=1,\\ i \neq s, s-1}}^{N_x} \sum_{\substack{j=1,\\ j \neq q}}^{N_y+1} \beta_-(j, N_i, e_i) \times \\ &\qquad \left\{ \bar{n}_{i+1}^j p([n_1^j, \ldots, \bar{n}_i^j, \bar{n}_{i+1}^j, \ldots, n_{N_x+1}^j], \mathbf{e}, t_h) - n_{i+1}^j p(\mathbf{n}, \mathbf{e}, t_h) \right\} \\ &= \sum_{\mathbf{n}} \sum_{\mathbf{e}} n_s^q \sum_{\substack{i=1,\\ i \neq s, s-1}}^{N_x} \sum_{\substack{j=1,\\ j \neq q}}^{N_y+1} \beta_-(j, N_i, e_i) \left\{ n_{i+1}^j p(\mathbf{n}, \mathbf{e}, t_h) - n_{i+1}^j p(\mathbf{n}, \mathbf{e}, t_h) \right\} \\ &= 0.\end{aligned}$$

Now consider $j \neq q$ with $i = s$. Applying the change of variables $\bar{n}_s^j = n_s^j - 1$ and $\bar{n}_{s+1}^j = n_{s+1}^j + 1$

and dropping the bars, we have

$$
\begin{aligned}
K_2 &= \sum_{\mathbf{n}} \sum_{\mathbf{e}} n_s^q \sum_{\substack{j=1,\\ j\neq q}}^{N_y+1} \beta_-(j, N_s, e_s) \times \\
&\qquad \Big\{ (n_{s+1}^j + 1) p([n_1^j, \ldots, n_s^j - 1, n_{s+1}^j + 1, \ldots, n_{N_x+1}^j], \mathbf{e}, t_h) - n_{s+1}^j p(\mathbf{n}, \mathbf{e}, t_h) \Big\} \\
&= \sum_{\mathbf{n}} \sum_{\mathbf{e}} n_s^q \sum_{\substack{j=1,\\ j\neq q}}^{N_y+1} \beta_-(j, N_s, e_s) \times \\
&\qquad \Big\{ \bar{n}_{s+1}^j p([n_1^j, \ldots, \bar{n}_s^j, \bar{n}_{s+1}^j, \ldots, n_{N_x+1}^j], \mathbf{e}, t_h) - n_{s+1}^j p(\mathbf{n}, \mathbf{e}, t_h) \Big\} \\
&= \sum_{\mathbf{n}} \sum_{\mathbf{e}} n_s^q \sum_{\substack{j=1,\\ j\neq q}}^{N_y+1} \beta_-(j, N_s, e_s) \Big\{ n_{s+1}^j p(\mathbf{n}, \mathbf{e}, t_h) - n_{s+1}^j p(\mathbf{n}, \mathbf{e}, t_h) \Big\} \\
&= 0.
\end{aligned}
$$

Next we look at $j \neq q$ with $i = s - 1$. If we apply the change of variables in the first term and drop the bars ($\bar{n}_s^j = n_s^j + 1$ and $\bar{n}_{s-1}^j = n_{s-1}^j - 1$), we have

$$
\begin{aligned}
K_3 &= \sum_{\mathbf{n}} \sum_{\mathbf{e}} n_s^q \sum_{\substack{j=1,\\ j\neq q}}^{N_y+1} \beta_-(j, N_{s-1}, e_{s-1}) \times \\
&\qquad \Big\{ (n_s^j + 1) p([n_1^j, \ldots, n_{s-1}^j - 1, n_s^j + 1, \ldots, n_{N_x+1}^j], \mathbf{e}, t_h) - n_s^j p(\mathbf{n}, \mathbf{e}, t_h) \Big\} \\
&= \sum_{\mathbf{n}} \sum_{\mathbf{e}} n_s^q \sum_{\substack{j=1,\\ j\neq q}}^{N_y+1} \beta_-(j, N_{s-1}, e_{s-1}) \times \\
&\qquad \Big\{ \bar{n}_s^j p([n_1^j, \ldots, \bar{n}_{s-1}^j, \bar{n}_s^j, \ldots, n_{N_x+1}^j], \mathbf{e}, t_h) - n_s^j p(\mathbf{n}, \mathbf{e}, t_h) \Big\} \\
&= \sum_{\mathbf{n}} \sum_{\mathbf{e}} n_s^q \sum_{\substack{j=1,\\ j\neq q}}^{N_y+1} \beta_-(j, N_{s-1}, e_{s-1}) \left\{ n_s^j p(\mathbf{n}, \mathbf{e}, t_h) - n_s^j p(\mathbf{n}, \mathbf{e}, t_h) \right\} \\
&= 0.
\end{aligned}
$$

Now we consider the three cases when $j = q$. First, when $i \neq s, s-1$, we apply the change of

variables $\bar{n}_i^q = n_i^q - 1$ and $\bar{n}_{i+1}^q = n_{i+1}^q + 1$ and dropping the bars, we have

$$
\begin{aligned}
K_4 &= \sum_{\mathbf{n}} \sum_{\mathbf{e}} n_s^q \sum_{\substack{i=1,\\ i\neq s,s-1}}^{N_x} \beta_-(q, N_i, e_i) \times \\
&\qquad \left\{ (n_{i+1}^q + 1) p([n_1^q, \ldots, n_i^q - 1, n_{i+1}^q + 1, \ldots, n_{N_x+1}^q], \mathbf{e}, t_h) - n_{i+1}^q p(\mathbf{n}, \mathbf{e}, t_h) \right\} \\
&= \sum_{\mathbf{n}} \sum_{\mathbf{e}} n_s^q \sum_{\substack{i=1,\\ i\neq s,s-1}}^{N_x} \beta_-(q, N_i, e_i) \times \\
&\qquad \left\{ \bar{n}_{i+1}^q p([n_1^q, \ldots, \bar{n}_i^q, \bar{n}_{i+1}^q, \ldots, n_{N_x+1}^q], \mathbf{e}, t_h) - n_{i+1}^q p(\mathbf{n}, \mathbf{e}, t_h) \right\} \\
&= \sum_{\mathbf{n}} \sum_{\mathbf{e}} n_s^q \sum_{\substack{i=1,\\ i\neq s,s-1}}^{N_x} \beta_-(q, N_i, e_i) \left\{ n_{i+1}^q p(\mathbf{n}, \mathbf{e}, t_h) - n_{i+1}^q p(\mathbf{n}, \mathbf{e}, t_h) \right\} \\
&= 0.
\end{aligned}
$$

Now consider $i = s$ and the change of variables $\bar{n}_s^q = n_s^q - 1$ and $\bar{n}_{s+1}^q = n_{s+1}^q + 1$ to give

$$
\begin{aligned}
K_5 &= \sum_{\mathbf{n}} \sum_{\mathbf{e}} n_s^q \beta_-(q, N_s, e_s) \times \\
&\qquad \left\{ (n_{s+1}^q + 1) p([n_1^q, \ldots, n_s^q - 1, n_{s+1}^q + 1, \ldots, n_{N_x+1}^q], \mathbf{e}, t_h) - n_{s+1}^q p(\mathbf{n}, \mathbf{e}, t_h) \right\} \\
&= \sum_{\mathbf{n}} \sum_{\mathbf{e}} \beta_-(q, N_s, e_s) \times \\
&\qquad \left\{ (\bar{n}_s^q + 1) \bar{n}_{s+1}^q p([n_1^q, \ldots, \bar{n}_s^q, \bar{n}_{s+1}^q, \ldots, n_{N_x+1}^q], \mathbf{e}, t_h) - n_s^q n_{s+1}^q p(\mathbf{n}, \mathbf{e}, t_h) \right\} \\
&= \sum_{\mathbf{n}} \sum_{\mathbf{e}} \beta_-(q, N_s, e_s) \left\{ (n_s^q + 1) n_{s+1}^q p(\mathbf{n}, \mathbf{e}, t_h) - n_s^q n_{s+1}^q p(\mathbf{n}, \mathbf{e}, t_h) \right\} \\
&= \sum_{\mathbf{n}} \sum_{\mathbf{e}} \beta_-(q, N_s, e_s) n_{s+1}^q p(\mathbf{n}, \mathbf{e}, t_h) \\
&= \langle \beta_-(q, N_s, e_s) n_{s+1}^q \rangle.
\end{aligned}
$$

Finally, consider $i = s - 1$ with the change of variables $\bar{n}^q_{s-1} = n^q_{s-1} - 1$ and $\bar{n}^q_s = n^q_s + 1$ to give

$$\begin{aligned}
K_6 = &\sum_{\mathbf{n}} \sum_{\mathbf{e}} n^q_s \beta_-(q, N_{s-1}, e_{s-1}) \times \\
&\qquad \left\{ (n^q_s + 1) p([n^q_1, \ldots, n^q_{s-1} - 1, n^q_s + 1, \ldots, n^q_{N_x+1}], \mathbf{e}, t_h) - n^q_s p(\mathbf{n}, \mathbf{e}, t_h) \right\} \\
&= \sum_{\mathbf{n}} \sum_{\mathbf{e}} \beta_-(q, N_{s-1}, e_{s-1}) \times \\
&\qquad \left\{ (\bar{n}^q_s - 1) \bar{n}^q_s p([n^q_1, \ldots, \bar{n}^q_{s-1}, \bar{n}^q_s, \ldots, n^q_{N_x+1}], \mathbf{e}, t_h) - n^q_s n^q_s p(\mathbf{n}, \mathbf{e}, t_h) \right\} \\
&= \sum_{\mathbf{n}} \sum_{\mathbf{e}} \beta_-(q, N_{s-1}, e_{s-1}) \left\{ (n^q_s - 1) n^q_s p(\mathbf{n}, \mathbf{e}, t_h) - n^q_s n^q_s p(\mathbf{n}, \mathbf{e}, t_h) \right\} \\
&= -\sum_{\mathbf{n}} \sum_{\mathbf{e}} \beta_-(q, N_{s-1}, e_{s-1}) n^q_s p(\mathbf{n}, \mathbf{e}, t_h) \\
&= -\langle \beta_-(q, N_{s-1}, e_{s-1}) n^q_s \rangle.
\end{aligned}$$

Bringing back together these terms, we have

$$\begin{aligned}
K &= K_1 + K_2 + K_3 + K_4 + K_5 + K_6 \\
&= \langle \beta_-(q, N_s, e_s) n^q_{s+1} \rangle - \langle \beta_-(q, N_{s-1}, e_{s-1}) n^q_s \rangle.
\end{aligned}$$

For the second movement term, we repeat this process, by breaking it down into six cases, defined by $j = q, j \neq q, i = s, i = s + 1$ and $i \neq s, s + 1$:

$$\begin{aligned}
\mathcal{L} &= \sum_{\mathbf{n}} \sum_{\mathbf{e}} n^q_s \sum_{i=2}^{N_x+1} \sum_{j=1}^{N_y+1} \beta_+(j, N_i, e_i) \left\{ (n^j_{i-1} + 1) p(L^{\mathrm{m}}_{i,j} \mathbf{n}, \mathbf{e}, t_h) - n^j_{i-1} p(\mathbf{n}, \mathbf{e}, t_h) \right\} \\
&= \mathcal{L}_1 + \mathcal{L}_2 + \mathcal{L}_3 + \mathcal{L}_4 + \mathcal{L}_5 + \mathcal{L}_6.
\end{aligned}$$

Then we start by considering $j \neq q$ and $i \neq s, s + 1$. Employing the change of variables

$\bar{n}_i^j = n_i^j - 1$ and $\bar{n}_{i-1}^j = n_{i-1}^j + 1$ and dropping the bars, we have

$$\mathcal{L}_1 = \sum_{\mathbf{n}} \sum_{\mathbf{e}} n_s^q \sum_{\substack{i=1, \\ i \neq s, s+1}}^{N_x} \sum_{\substack{j=1, \\ j \neq q}}^{N_y+1} \beta_+(j, N_i, e_i) \times$$

$$\left\{ (n_{i-1}^j + 1) p([n_1^j, \ldots, n_{i-1}^j + 1, n_i^j - 1, \ldots, n_{N_x+1}^j], \mathbf{e}, t_h) - n_{i-1}^j p(\mathbf{n}, \mathbf{e}, t_h) \right\}$$

$$= \sum_{\mathbf{n}} \sum_{\mathbf{e}} n_s^q \sum_{\substack{i=1, \\ i \neq s, s+1}}^{N_x} \sum_{\substack{j=1, \\ j \neq q}}^{N_y+1} \beta_+(j, N_i, e_i) \times$$

$$\left\{ \bar{n}_{i-1}^j p([n_1^j, \ldots, \bar{n}_{i-1}^j, \bar{n}_i^j, \ldots, n_{N_x+1}^j], \mathbf{e}, t_h) - n_{i-1}^j p(\mathbf{n}, \mathbf{e}, t_h) \right\}$$

$$= \sum_{\mathbf{n}} \sum_{\mathbf{e}} n_s^q \sum_{\substack{i=1, \\ i \neq s, s+1}}^{N_x} \sum_{\substack{j=1, \\ j \neq q}}^{N_y+1} \beta_+(j, N_i, e_i) \left\{ n_{i-1}^j p(\mathbf{n}, \mathbf{e}, t_h) - n_{i-1}^j p(\mathbf{n}, \mathbf{e}, t_h) \right\}$$

$$= 0.$$

Now consider $j \neq q$ with $i = s$. Then, applying the change of variables $\bar{n}_s^j = n_s^j - 1$ and $\bar{n}_{s-1}^j = n_{s-1}^j + 1$ and dropping the bars, we have

$$\mathcal{L}_2 = \sum_{\mathbf{n}} \sum_{\mathbf{e}} n_s^q \sum_{\substack{j=1, \\ j \neq q}}^{N_y+1} \beta_+(j, N_s, e_s) \times$$

$$\left\{ (n_{s-1}^j + 1) p([n_1^j, \ldots, n_{s-1}^j + 1, n_s^j - 1, \ldots, n_{N_x+1}^j], \mathbf{e}, t_h) - n_{s+1}^j p(\mathbf{n}, \mathbf{e}, t_h) \right\}$$

$$= \sum_{\mathbf{n}} \sum_{\mathbf{e}} n_s^q \sum_{\substack{j=1, \\ j \neq q}}^{N_y+1} \beta_+(j, N_s, e_s) \times$$

$$\left\{ \bar{n}_{s-1}^j p([n_1^j, \ldots, \bar{n}_{s-1}^j, \bar{n}_s^j, \ldots, n_{N_x+1}^j], \mathbf{e}, t_h) - n_{s-1}^j p(\mathbf{n}, \mathbf{e}, t_h) \right\}$$

$$= \sum_{\mathbf{n}} \sum_{\mathbf{e}} n_s^q \sum_{\substack{j=1, \\ j \neq q}}^{N_y+1} \beta_+(j, N_s, e_s) \left\{ n_{s+1}^j p(\mathbf{n}, \mathbf{e}, t_h) - n_{s-1}^j p(\mathbf{n}, \mathbf{e}, t_h) \right\}$$

$$= 0.$$

Next we look at $j \neq q$ with $i = s + 1$. If we apply the change of variables in the first term and

drop the bars ($\bar{n}_s^j = n_s^j + 1$ and $\bar{n}_{s+1}^j = n_{s+1}^j - 1$), we have

$$
\begin{aligned}
\mathcal{L}_3 &= \sum_{\mathbf{n}} \sum_{\mathbf{e}} n_s^q \sum_{\substack{j=1,\\ j\neq q}}^{N_y+1} \beta_+(j, N_{s-1}, e_{s-1}) \times \\
&\qquad \Big\{ (n_s^j + 1) p([n_1^j, \ldots, n_s^j + 1, n_{s+1}^j - 1, \ldots, n_{N_x+1}^j], \mathbf{e}, t_h) - n_s^j p(\mathbf{n}, \mathbf{e}, t_h) \Big\} \\
&= \sum_{\mathbf{n}} \sum_{\mathbf{e}} n_s^q \sum_{\substack{j=1,\\ j\neq q}}^{N_y+1} \beta_+(j, N_{s-1}, e_{s-1}) \times \\
&\qquad \Big\{ \bar{n}_s^j p([n_1^j, \ldots, \bar{n}_s^j, \bar{n}_{s+1}^j, \ldots, n_{N_x+1}^j], \mathbf{e}, t_h) - n_s^j p(\mathbf{n}, \mathbf{e}, t_h) \Big\} \\
&= \sum_{\mathbf{n}} \sum_{\mathbf{e}} n_s^q \sum_{\substack{j=1,\\ j\neq q}}^{N_y+1} \beta_+(j, N_{s-1}, e_{s-1}) \left\{ n_s^j p(\mathbf{n}, \mathbf{e}, t_h) - n_s^j p(\mathbf{n}, \mathbf{e}, t_h) \right\} \\
&= 0.
\end{aligned}
$$

Now we consider the three cases when $j = q$. First, when $i \neq s, s+1$, applying the change of variables $\bar{n}_i^q = n_i^q - 1$ and $\bar{n}_{i-1}^q = n_{i-1}^q + 1$ and dropping the bars, we have

$$
\begin{aligned}
\mathcal{L}_4 &= \sum_{\mathbf{n}} \sum_{\mathbf{e}} n_s^q \sum_{\substack{i=1,\\ i\neq s, s-1}}^{N_x} \beta_+(q, N_i, e_i) \times \\
&\qquad \left\{ (n_{i-1}^q + 1) p([n_1^q, \ldots, n_{i-1}^q + 1, n_i^q - 1, \ldots, n_{N_x+1}^q], \mathbf{e}, t_h) - n_{i-1}^q p(\mathbf{n}, \mathbf{e}, t_h) \right\} \\
&= \sum_{\mathbf{n}} \sum_{\mathbf{e}} n_s^q \sum_{\substack{i=1,\\ i\neq s, s-1}}^{N_x} \beta_+(q, N_i, e_i) \times \\
&\qquad \left\{ \bar{n}_{i-1}^q p([n_1^q, \ldots, \bar{n}_{i-1}^q, \bar{n}_i^q, \ldots, n_{N_x+1}^q], \mathbf{e}, t_h) - n_{i-1}^q p(\mathbf{n}, \mathbf{e}, t_h) \right\} \\
&= \sum_{\mathbf{n}} \sum_{\mathbf{e}} n_s^q \sum_{\substack{i=1,\\ i\neq s, s-1}}^{N_x} \beta_+(q, N_i, e_i) \left\{ n_{i-1}^q p(\mathbf{n}, \mathbf{e}, t_h) - n_{i-1}^q p(\mathbf{n}, \mathbf{e}, t_h) \right\} \\
&= 0.
\end{aligned}
$$

Now for $i = s$ and the change of variables $\bar{n}_s^q = n_s^q - 1$ and $\bar{n}_{s-1}^q = n_{s-1}^q + 1$, we have

$$
\begin{aligned}
\mathcal{L}_5 = &\sum_{\mathbf{n}} \sum_{\mathbf{e}} n_s^q \beta_+(q, N_s, e_s) \times \\
&\qquad \left\{ (n_{s-1}^q + 1) p([n_1^q, \ldots, n_{s-1}^q + 1, n_s^q - 1, \ldots, n_{N_x+1}^q], \mathbf{e}, t_h) - n_{s-1}^q p(\mathbf{n}, \mathbf{e}, t_h) \right\} \\
= &\sum_{\mathbf{n}} \sum_{\mathbf{e}} \beta_+(q, N_s, e_s) \times \\
&\qquad \left\{ (\bar{n}_s^q + 1) \bar{n}_{s-1}^q p([n_1^q, \ldots, \bar{n}_{s-1}^q, \bar{n}_s^q, \ldots, n_{N_x+1}^q], \mathbf{e}, t_h) - n_s^q n_s^q p(\mathbf{n}, \mathbf{e}, t_h) \right\} \\
= &\sum_{\mathbf{n}} \sum_{\mathbf{e}} \beta_+(q, N_s, e_s) \left\{ (n_s^q + 1) n_{s-1}^q p(\mathbf{n}, \mathbf{e}, t_h) - n_s^q n_{s-1}^q p(\mathbf{n}, \mathbf{e}, t_h) \right\} \\
= &\sum_{\mathbf{n}} \sum_{\mathbf{e}} \beta_+(q, N_s, e_s) n_{s-1}^q p(\mathbf{n}, \mathbf{e}, t_h) \\
= &\langle \beta_+(q, N_s, e_s) n_{s-1}^q \rangle.
\end{aligned}
$$

Finally, for $i = s + 1$ with the change of variables $\bar{n}_{s+1}^q = n_{s+1}^q - 1$ and $\bar{n}_s^q = n_s^q + 1$, we have

$$
\begin{aligned}
\mathcal{L}_6 = &\sum_{\mathbf{n}} \sum_{\mathbf{e}} n_s^q \beta_+(q, N_{s+1}, e_{s+1}) \times \\
&\qquad \left\{ (n_s^q + 1) p([n_1^q, \ldots, n_s^q + 1, n_{s+1}^q - 1, \ldots, n_{N_x+1}^q], \mathbf{e}, t_h) - n_s^q p(\mathbf{n}, \mathbf{e}, t_h) \right\} \\
= &\sum_{\mathbf{n}} \sum_{\mathbf{e}} \beta_+(q, N_{s+1}, e_{s+1}) \times \\
&\qquad \left\{ (\bar{n}_s^q - 1) \bar{n}_s^q p([n_1^q, \ldots, \bar{n}_s^q, \bar{n}_{s+1}^q, \ldots, n_{N_x+1}^q], \mathbf{e}, t_h) - n_s^q n_s^q p(\mathbf{n}, \mathbf{e}, t_h) \right\} \\
= &\sum_{\mathbf{n}} \sum_{\mathbf{e}} \beta_+(q, N_{s+1}, e_{s+1}) \left\{ (n_s^q - 1) n_s^q p(\mathbf{n}, \mathbf{e}, t_h) - n_s^q n_s^q p(\mathbf{n}, \mathbf{e}, t_h) \right\} \\
= &-\sum_{\mathbf{n}} \sum_{\mathbf{e}} \beta_+(q, N_{s+1}, e_{s+1}) n_s^q p(\mathbf{n}, \mathbf{e}, t_h) \\
= &-\langle \beta_+(q, N_{s+1}, e_{s+1}) n_s^q \rangle.
\end{aligned}
$$

Bringing back together these terms, we have

$$
\begin{aligned}
\mathcal{L} &= \mathcal{L}_1 + \mathcal{L}_2 + \mathcal{L}_3 + \mathcal{L}_4 + \mathcal{L}_5 + \mathcal{L}_6 \\
&= \langle \beta_+(q, N_s, e_s) n_{s-1}^q \rangle - \langle \beta_+(q, N_{s+1}, e_{s+1}) n_s^q \rangle.
\end{aligned}
$$

Finally, we must consider the last term in Eq. (S1)

$$
\begin{aligned}
M &= \sum_{\mathbf{n}}\sum_{\mathbf{e}} n_s^q \sum_{i=1}^{N_x+1}\sum_{j=1}^{N_y+1} \Big\{\gamma(j, N_i-1, e_i)(n_i^j-1)p(G_{i,j}\mathbf{n}, \mathbf{e}, t_h) \\
&\qquad\qquad - \gamma(j, N_i, e_i)n_i^j p(\mathbf{n}, \mathbf{e}, t_h)\Big\} \\
&= \sum_{\mathbf{n}}\sum_{\mathbf{e}} n_s^q \sum_{i=1}^{N_x+1}\sum_{j=1}^{N_y+1} \Big\{\gamma(j, N_i-1, e_i)(n_i^j-1)p([n_1^j, \ldots, n_i^j-1, \ldots, n_{N_x+1}^j], \mathbf{e}, t_h) \\
&\qquad\qquad - \gamma(j, N_i, e_i)n_i^j p(\mathbf{n}, \mathbf{e}, t_h)\Big\}, \\
&= M_1 + M_2 + M_3 + M_4 + M_5 + M_6.
\end{aligned}
$$

Again we consider the cases $i = s, i \neq s, j = q, j \neq q$. First, begin with $i \neq s$ and $j \neq q$, and use the change of variable $\bar{n}_i^j = n_i^j - 1$, noticing that $N_i - 1$ becomes $\bar{N}_i$ when we change the variable. Dropping the bars gives

$$
\begin{aligned}
M_1 &= \sum_{\mathbf{n}}\sum_{\mathbf{e}} n_s^q \sum_{i=1}^{N_x+1}\sum_{j=1}^{N_y+1} \Big\{\gamma(j, N_i-1, e_i)(n_i^j-1)p([n_1^j, \ldots, n_i^j-1, \ldots, n_{N_x+1}^j], \mathbf{e}, t_h) \\
&\qquad\qquad - \gamma(j, N_i, e_i)n_i^j p(\mathbf{n}, \mathbf{e}, t_h)\Big\} \\
&= \sum_{\mathbf{n}}\sum_{\mathbf{e}} n_s^q \sum_{\substack{i=1,\\ i\neq s}}^{N_x+1}\sum_{\substack{j=1,\\ j\neq q}}^{N_y+1} \Big\{\gamma(j, \bar{N}_i, e_i)\bar{n_i}^j p([n_1^j, \ldots, \bar{n}_i^j, \ldots, n_{N_x+1}^j], \mathbf{e}, t_h) \\
&\qquad\qquad - \gamma(j, N_i, e_i)n_i^j p(\mathbf{n}, \mathbf{e}, t_h)\Big\} \\
&= \sum_{\mathbf{n}}\sum_{\mathbf{e}} n_s^q \sum_{\substack{i=1,\\ i\neq s}}^{N_x+1}\sum_{\substack{j=1,\\ j\neq q}}^{N_y+1} \gamma(j, N_i, e_i)\Big\{n_i^j p(\mathbf{n}, \mathbf{e}, t_h) - n_i^j p(\mathbf{n}, \mathbf{e}, t_h)\Big\} \\
&= 0.
\end{aligned}
$$

Now consider $i \neq s$ and $j = q$. Using the change of variable $\bar{n}_i^q = n_i^q - 1$, along with the newly

defined $\bar{N}_i$, before dropping the bars we have

$$\begin{aligned}
M_2 &= \sum_{\mathbf{n}}\sum_{\mathbf{e}} n_s^q \sum_{\substack{i=1,\\ i\neq s}}^{N_x+1} \Big\{\gamma(q, N_i-1, e_i)(n_i^q-1)p([n_1^q,\ldots,n_i^q-1,\ldots,n_{N_x+1}^q],\mathbf{e},t_h) \\
&\qquad\qquad\qquad\qquad\qquad - \gamma(q,N_i,e_i)n_i^q p(\mathbf{n},\mathbf{e},t_h)\Big\} \\
&= \sum_{\mathbf{n}}\sum_{\mathbf{e}} n_s^q \sum_{\substack{i=1,\\ i\neq s}}^{N_x+1} \Big\{\gamma(q, \bar{N}_i, e_i)\bar{n}_i^q p([n_1^q,\ldots,\bar{n}_i^q,\ldots,n_{N_x+1}^q],\mathbf{e},t_h) \\
&\qquad\qquad\qquad\qquad\qquad - \gamma(q,N_i,e_i)n_i^q p(\mathbf{n},\mathbf{e},t_h)\Big\} \\
&= \sum_{\mathbf{n}}\sum_{\mathbf{e}} n_s^q \sum_{\substack{i=1,\\ i\neq s}}^{N_x+1} \gamma(q,N_i,e_i)\left\{n_i^q p(\mathbf{n},\mathbf{e},t_h) - n_i^q p(\mathbf{n},\mathbf{e},t_h)\right\} \\
&= 0.
\end{aligned}$$

Now take $i = s$ and consider $j \neq q$. Using the following change of variables, $\bar{n}_s^j = n_s^j - 1$ and $\bar{N}_s = N_s - 1$, we see that

$$\begin{aligned}
M_3 &= \sum_{\mathbf{n}}\sum_{\mathbf{e}} n_s^q \sum_{\substack{j=1,\\ j\neq q}}^{N_y+1} \Big\{\gamma(j, N_s-1, e_s)(n_s^j-1)p([n_1^j,\ldots,n_s^j-1,\ldots,n_{N_x+1}^j],\mathbf{e},t_h) \\
&\qquad\qquad\qquad\qquad\qquad - \gamma(j,N_s,e_s)n_s^j p(\mathbf{n},\mathbf{e},t_h)\Big\} \\
&= \sum_{\mathbf{n}}\sum_{\mathbf{e}} n_s^q \sum_{\substack{j=1,\\ j\neq q}}^{N_y+1} \Big\{\gamma(j, \bar{N}_s, e_s)\bar{n}_s^j p([n_1^j,\ldots,\bar{n}_s^j,\ldots,n_{N_x+1}^j],\mathbf{e},t_h) \\
&\qquad\qquad\qquad\qquad\qquad - \gamma(j,N_s,e_s)n_s^j p(\mathbf{n},\mathbf{e},t_h)\Big\} \\
&= \sum_{\mathbf{n}}\sum_{\mathbf{e}} n_s^q \sum_{\substack{j=1,\\ j\neq q}}^{N_y+1} \gamma(j,N_s,e_s)\left\{n_s^j p(\mathbf{n},\mathbf{e},t_h) - n_s^j p(\mathbf{n},\mathbf{e},t_h)\right\} \\
&= 0.
\end{aligned}$$

Finally, consider $i = s$ and $j = q$ with the change of variable $\bar{n}_s^q = n_s^q - 1$ along with $\bar{N}_s$ in the

second term, and then drop the bar to give

$$
\begin{aligned}
M_4 &= \sum_{\mathbf{n}} \sum_{\mathbf{e}} n_s^q \Big\{ \gamma(q, N_s - 1, e_s)(n_s^q - 1) p([n_1^q, \ldots, n_s^q - 1, \ldots, n_{N_x+1}^q], \mathbf{e}, t_h) \\
&\qquad\qquad\qquad\qquad\qquad\qquad\qquad\qquad - \gamma(q, N_s, e_s) n_s^q p(\mathbf{n}, \mathbf{e}, t_h) \Big\} \\
&= \sum_{\mathbf{n}} \sum_{\mathbf{e}} \Big\{ \gamma(q, \bar{N}_s, e_s) \bar{n}_s^q (\bar{n}_s^q + 1) p([n_1^q, \ldots, \bar{n}_s^q, \ldots, n_{N_x+1}^q], \mathbf{e}, t_h) \\
&\qquad\qquad\qquad\qquad - \gamma(q, N_s, e_s)(n_s^q)^2 p(\mathbf{n}, \mathbf{e}, t_h) \Big\} \\
&= \sum_{\mathbf{n}} \sum_{\mathbf{e}} n_s^q \gamma(q, N_s, e_s) p(\mathbf{n}, \mathbf{e}, t_h) \\
&= \langle \gamma(q, N_s, e_s) n_s^q \rangle.
\end{aligned}
$$

Combining all of these calculations, we can rewrite the master equation, Eq. (S1), as

$$
\begin{aligned}
\frac{\partial}{\partial t} \langle n_s^q \rangle = & \frac{1}{\Delta_t} \langle \beta_+(q, N_s, e_s) n_{s-1}^q \rangle + \frac{1}{\Delta_t} \langle \beta_-(q, N_s, e_s) n_{s+1}^q \rangle \\
& - \frac{1}{\Delta_t} \langle \beta_-(q, N_{s-1}, e_{s-1}) n_s^q \rangle - \frac{1}{\Delta_t} \langle \beta_+(q, N_{s+1}, e_{s+1}) n_s^q \rangle \\
& + \frac{1}{\Delta_t} \langle \mu_+(q-1, N_s, e_s) n_s^{q-1} \rangle + \frac{1}{\Delta_t} \langle \mu_-(q+1, N_s, e_s) n_s^{q+1} \rangle \\
& - \frac{1}{\Delta_t} \langle \mu_+(q, N_s, e_s) n_s^q \rangle - \frac{1}{\Delta_t} \langle \mu_-(q, N_s, e_s) n_s^q \rangle \\
& + \frac{1}{\Delta_t} \langle \gamma(q, N_s, e_s) n_s^q \rangle. \qquad \text{(S3)}
\end{aligned}
$$

This mean equation is related to a partial differential equation model in the appropriate limits as $\Delta_x \to 0$, $\Delta_y \to 0$ and $\Delta_t \to 0$ simultaneously, and the discrete values of $\langle n_i^j(t_h) \rangle$ and $\langle e_i(t_h) \rangle$ are written in terms of the continuous variables $n(x, y, t)$ and $e(x, t)$, respectively. Eq. (S3) can

be rewritten as follows, using mean-field approximations,

$$
\begin{aligned}
\frac{\partial n(x,y,t)}{\partial t} &= \frac{1}{\Delta_t}\beta_+(y,\rho(x,t),e(x,t))n(x-\Delta_x,y,t) \\
&+ \frac{1}{\Delta_t}\beta_-(y,\rho(x,t),e(x,t))n(x+\Delta_x,y,t) \\
&- \frac{1}{\Delta_t}\beta_-(y,\rho(x-\Delta_x,t),e(x-\Delta_x,t))n(x,y,t) \\
&- \frac{1}{\Delta_t}\beta_+(y,\rho(x+\Delta_x,t),e(x+\Delta_x,t))n(x,y,t) \\
&+ \frac{1}{\Delta_t}\mu_+(y-\Delta_y,\rho(x,t),e(x,t))n(x,y-\Delta_y,t) \\
&+ \frac{1}{\Delta_t}\mu_-(y+\Delta_y,\rho(x,t),e(x,t))n(x,y+\Delta_y,t) \\
&- \frac{1}{\Delta_t}\mu_+(y,\rho(x,t),e(x,t))n(x,y,t) \\
&- \frac{1}{\Delta_t}\mu_-(y,\rho(x,t),e(x,t))n(x,y,t) \\
&+ \frac{1}{\Delta_t}\gamma(y,\rho(x,t),e(x,t))n(x,y,t). \qquad \text{(S4)}
\end{aligned}
$$

Then, we can employ Taylor series expansions in Eq. (S4) and take limits to give

$$
\begin{aligned}
\frac{\partial n(x,y,t)}{\partial t} &= \frac{1}{\Delta_t}\beta_+(y,\rho(x,t),e(x,t))\left[n(x,y,t) - \Delta_x\frac{\partial}{\partial x}n(x,y,t) + \frac{\Delta_x^2}{2}\frac{\partial^2}{\partial x^2}n(x,y,t)\right] \\
&+ \frac{1}{\Delta_t}\beta_-(y,\rho(x,t),e(x,t))\left[n(x,y,t) + \Delta_x\frac{\partial}{\partial x}n(x,y,t) + \frac{\Delta_x^2}{2}\frac{\partial^2}{\partial x^2}n(x,y,t)\right] \\
&- \frac{1}{\Delta_t}\left[\beta_-(y,\rho,e) - \Delta_x\frac{\partial}{\partial x}\beta_-(y,\rho,e) + \frac{\Delta_x^2}{2}\frac{\partial^2}{\partial x^2}\beta_-(y,\rho,e)\right]n(x,y,t) \\
&- \frac{1}{\Delta_t}\left[\beta_+(y,\rho,e) + \Delta_x\frac{\partial}{\partial x}\beta_+(y,\rho,e) + \frac{\Delta_x^2}{2}\frac{\partial^2}{\partial x^2}\beta_+(y,\rho,e)\right]n(x,y,t) \\
&+ \frac{1}{\Delta_t}\left[\mu_+(y,\rho,e) - \Delta_y\frac{\partial}{\partial y}\mu_+(y,\rho,e) + \frac{\Delta_y^2}{2}\frac{\partial^2}{\partial y^2}\mu_+(y,\rho,e)\right]\times \\
&\qquad \left[n(x,y,t) - \Delta_y\frac{\partial}{\partial y}n(x,y,t) + \frac{\Delta_y^2}{2}\frac{\partial^2}{\partial y^2}n(x,y,t)\right] \\
&+ \frac{1}{\Delta_t}\left[\mu_-(y,\rho,e) + \Delta_y\frac{\partial}{\partial y}\mu_-(y,\rho,e) + \frac{\Delta_y^2}{2}\frac{\partial^2}{\partial y^2}\mu_-(y,\rho,e)\right]\times \\
&\qquad \left[n(x,y,t) + \Delta_y\frac{\partial}{\partial y}n(x,y,t) + \frac{\Delta_y^2}{2}\frac{\partial^2}{\partial y^2}n(x,y,t)\right] \\
&- \frac{1}{\Delta_t}\mu_+(y,\rho(x,t),e(x,t))n(x,y,t) - \frac{1}{\Delta_t}\mu_-(y,\rho(x,t),e(x,t))n(x,y,t) \\
&+ \frac{1}{\Delta_t}\gamma(y,\rho(x,t),e(x,t))n(x,y,t) + \mathrm{O}(\Delta_x^3) + \mathrm{O}(\Delta_y^3) + \mathrm{O}(\Delta_t^2).
\end{aligned}
$$

Rearranging and collecting terms, we obtain

$$
\begin{aligned}
\frac{\partial}{\partial t}n(x,y,t) = {} & \frac{\Delta_x}{\Delta_t}\frac{\partial}{\partial x}\Big(\left(\beta_-(y,\rho(x,t),e(x,t)) - \beta_+(y,\rho(x,t),e(x,t))\right) n\Big) \\
& + \frac{\Delta_x^2}{2\Delta_t}\frac{\partial}{\partial x}\Bigg(\Big(\beta_-\left(y,\rho(x,t),e(x,t)\right) + \beta_+\left(y,\rho(x,t),e(x,t)\right)\Big)\frac{\partial}{\partial x}n(x,y,t) \\
& \qquad - n(x,y,t)\frac{\partial}{\partial x}\Big(\beta_-\left(y,\rho(x,t),e(x,t)\right) + \beta_+\left(y,\rho(x,t),e(x,t)\right)\Big)\Bigg) \\
& + \frac{\Delta_y}{\Delta_t}\frac{\partial}{\partial y}\left(\Big(\mu_-\left(y,\rho(x,t),e(x,t)\right) - \mu_+\left(y,\rho(x,t),e(x,t)\right)\Big)n(x,y,t)\right) \\
& + \frac{\Delta_y^2}{2\Delta_t}\frac{\partial^2}{\partial y^2}\left(\Big(\mu_-\left(y,\rho(x,t),e(x,t)\right) + \mu_+\left(y,\rho(x,t),e(x,t)\right)\Big)n(x,y,t)\right) \\
& + \frac{1}{\Delta_t}\gamma\left(y,\rho(x,t),e(x,t)\right)n(x,y,t).
\end{aligned}
$$

We take the parabolic limit as Δ_x, Δ_y, $\Delta_t \to 0$ simultaneously (assuming $n(x,y,t) \sim O(1)$), and define

$$
\begin{aligned}
\lim_{\Delta_x,\Delta_t\to 0}\frac{\Delta_x}{\Delta_t}\Big(\beta_-(y,\rho(x,t),e(x,t)) - \beta_+(y,\rho(x,t),e(x,t))\Big) &= v^m(y,\rho(x,t),e(x,t)), \\
\lim_{\Delta_x,\Delta_t\to 0}\frac{\Delta_x^2}{2\Delta_t}\Big(\beta_-(y,\rho(x,t),e(x,t)) + \beta_+(y,\rho(x,t),e(x,t))\Big) &= D^m(y,\rho(x,t),e(x,t)), \\
\lim_{\Delta_y,\Delta_t\to 0}\frac{\Delta_y}{\Delta_t}\Big(\mu_-\left(y,\rho(x,t),e(x,t)\right) - \mu_+\left(y,\rho(x,t),e(x,t)\right)\Big) &= v^p(y,\rho(x,t),e(x,t)), \\
\lim_{\Delta_y,\Delta_t\to 0}\frac{\Delta_y^2}{2\Delta_t}\Big(\mu_-\left(y,\rho(x,t),e(x,t)\right) + \mu_+\left(y,\rho(x,t),e(x,t)\right)\Big) &= D^p(y,\rho(x,t),e(x,t)), \\
\lim_{\Delta_t\to 0}\frac{1}{\Delta_t}\gamma\left(y,\rho(x,t),e(x,t)\right) &= r(y,\rho(x,t),e(x,t)).
\end{aligned}
$$

The final equation for the evolution of the cell density is therefore given by

$$
\begin{aligned}
\frac{\partial}{\partial t}n(x,y,t) = {} & \frac{\partial}{\partial x}\Big(v^m(y,\rho(x,t),e(x,t))n\Big) \\
& + \frac{\partial}{\partial x}\Bigg(D^m\left(y,\rho(x,t),e(x,t)\right)\frac{\partial}{\partial x}n(x,y,t) \\
& \qquad - n(x,y,t)\frac{\partial}{\partial x}D^m\left(y,\rho(x,t),e(x,t)\right)\Bigg) \\
& + \frac{\partial}{\partial y}\left(v^p\left(y,\rho(x,t),e(x,t)\right)n(x,y,t)\right) \\
& + \frac{\partial^2}{\partial y^2}\left(D^p\left(y,\rho(x,t),e(x,t)\right)n(x,y,t)\right) \\
& + r\left(y,\rho(x,t),e(x,t)\right)n(x,y,t).
\end{aligned}
$$

S1.1.1 Model equations on the boundaries

We can repeat the above analysis for the boundaries of physical and phenotype space in order to retrieve the boundary equations. We note here that when looking at the boundaries for the cell equation, all terms involving change in the number of elements of the local environment provide no contribution and can be ignored hereon in.

Boundary condition at $x = X_{\mathbf{min}}$. Revisiting Eq. (S1) to derive the equation on the left-most lattice site, we multiply by n_1^q and sum over all possible states $\mathbf{n}$ and $\mathbf{e}$:

$$\begin{aligned}
&\Delta_t \sum_{\mathbf{n}} \sum_{\mathbf{e}} n_1^q \frac{\partial}{\partial t} p(\mathbf{n}, \mathbf{e}, t_h) + O(\Delta_t^2) \\
&= \sum_{\mathbf{n}} \sum_{\mathbf{e}} n_1^q \sum_{i=1}^{N_x+1} \sum_{j=1}^{N_y} \mu_-(j+1, N_i, e_i) \left\{ (n_i^{j+1} + 1) p(U_{i,j}^{\mathrm{p}} \mathbf{n}, \mathbf{e}, t_h) - n_i^{j+1} p(\mathbf{n}, \mathbf{e}, t_h) \right\} \\
&+ \sum_{\mathbf{n}} \sum_{\mathbf{e}} n_1^q \sum_{i=1}^{N_x+1} \sum_{j=2}^{N_y+1} \mu_+(j-1, N_i, e_i) \left\{ (n_i^{j-1} + 1) p(D_{i,j}^{\mathrm{p}} \mathbf{n}, \mathbf{e}, t_h) - n_i^{j-1} p(\mathbf{n}, \mathbf{e}, t_h) \right\} \\
&+ \sum_{\mathbf{n}} \sum_{\mathbf{e}} n_1^q \sum_{i=1}^{N_x} \sum_{j=1}^{N_y+1} \beta_-(j, N_i, e_i) \left\{ (n_{i+1}^{j} + 1) p(R_{i,j}^{\mathrm{m}} \mathbf{n}, \mathbf{e}, t_h) - n_{i+1}^{j} p(\mathbf{n}, \mathbf{e}, t_h) \right\} \\
&+ \sum_{\mathbf{n}} \sum_{\mathbf{e}} n_1^q \sum_{i=2}^{N_x+1} \sum_{j=1}^{N_y+1} \beta_+(j, N_i, e_i) \left\{ (n_{i-1}^{j} + 1) p(L_{i,j}^{\mathrm{m}} \mathbf{n}, \mathbf{e}, t_h) - n_{i-1}^{j} p(\mathbf{n}, \mathbf{e}, t_h) \right\} \\
&+ \sum_{\mathbf{n}} \sum_{\mathbf{e}} n_1^q \sum_{i=1}^{N_x+1} \sum_{j=1}^{N_y+1} \left\{ \gamma(j, N_i - 1, e_i)(n_i^j - 1) p(G_{i,j} \mathbf{n}, \mathbf{e}, t_h) - \gamma(j, N_i, e_i) n_i^j p(\mathbf{n}, \mathbf{e}, t_h) \right\}.
\end{aligned} \tag{S5}$$

From earlier working, we know that all terms describing a movement in physical space give non-zero contributions when $j \neq q$, and hence can be ignored. Now, starting by considering the first term on the right-hand side of Eq. (S5), we consider the non-zero contributions from the cases $j = q$ and $j = q - 1$. Using $j = q$:

$$\begin{aligned}
&\sum_{\mathbf{n}} \sum_{\mathbf{e}} n_1^q \sum_{i=1}^{N_x+1} \mu_-(q+1, N_i, e_i) \left\{ (n_i^{q+1} + 1) p(U_{i,q}^{\mathrm{p}} \mathbf{n}, \mathbf{e}, t_h) - n_i^{q+1} p(\mathbf{n}, \mathbf{e}, t_h) \right\} \\
&\quad = \sum_{\mathbf{n}} \sum_{\mathbf{e}} n_1^q \sum_{i=1}^{N_x+1} \mu_-(q+1, N_i, e_i) \times \\
&\qquad \left\{ (n_i^{q+1} + 1) p([n_i^1, \dots, n_i^q - 1, n_i^{q+1} + 1, \dots, n_i^{N_y+1}], \mathbf{e}, t_h) - n_i^{q+1} p(\mathbf{n}, \mathbf{e}, t_h) \right\}.
\end{aligned} \tag{S6}$$

As per previous calculations, the only contributions here will come from terms where $i = 1$. Looking at these, we see that if we employ the change of variables $\bar{n}_1^{q+1} = n_1^{q+1} + 1$ and $\bar{n}_1^q = n_1^q - 1$

in the first term, then we can rewrite the right-hand side of Eq. (S6) as

$$
\begin{aligned}
&\sum_{\mathbf{n}}\sum_{\mathbf{e}} \mu_-(q+1, N_1, e_1)\times \\
&\qquad \left\{\bar{n}_1^{q+1}(\bar{n}_1^q+1)p([n_1^1,\ldots,\bar{n}_1^q,\bar{n}_1^{q+1},\ldots,n_1^{N_y+1}],\mathbf{e},t_h) - n_1^q n_1^{q+1} p(\mathbf{n},\mathbf{e},t_h)\right\} \\
&= \sum_{\mathbf{n}}\sum_{\mathbf{e}} \mu_-(q+1, N_1, e_1)\left\{n_1^{q+1}(n_1^q+1)p(\mathbf{n},\mathbf{e},t_h) - n_1^q n_1^{q+1} p(\mathbf{n},\mathbf{e},t_h)\right\} \\
&= \sum_{\mathbf{n}}\sum_{\mathbf{e}} \mu_-(q+1, N_1, e_1) n_1^{q+1} p(\mathbf{n},\mathbf{e},t_h) \\
&= \langle \mu_-(q+1, N_1, e_1) n_1^{q+1}\rangle.
\end{aligned}
$$

Using $j = q-1$ and $i = 1$, with the change of variables $\bar{n}_1^{q-1} = n_1^{q-1} - 1$ and $\bar{n}_1^q = n_1^q + 1$ in the first term of Eq. (S5), then we have

$$
\begin{aligned}
&\sum_{\mathbf{n}}\sum_{\mathbf{e}} n_1^q \mu_-(q, N_1, e_1)\times \\
&\qquad \left\{(n_1^q+1)p([n_1^1,\ldots,n_1^{q-1}-1,n_1^q+1,\ldots,n_1^{N_y+1}],\mathbf{e},t_h) - n_1^q p(\mathbf{n},\mathbf{e},t_h)\right\} \\
&= \sum_{\mathbf{n}}\sum_{\mathbf{e}} \mu_-(q, N_1, e_1)\times \\
&\qquad \left\{\bar{n}_1^q(\bar{n}_1^q-1)p([n_1^1,\ldots,\bar{n}_1^{q-1},\bar{n}_1^q,\ldots,n_1^{N_y+1}],\mathbf{e},t_h) - n_1^q n_1^q p(\mathbf{n},\mathbf{e},t_h)\right\} \\
&= \sum_{\mathbf{n}}\sum_{\mathbf{e}} \mu_-(q, N_1, e_1)\left\{n_1^q(n_1^q-1)p(\mathbf{n},\mathbf{e},t_h) - n_1^q n_1^q p(\mathbf{n},\mathbf{e},t_h)\right\} \\
&= -\sum_{\mathbf{n}}\sum_{\mathbf{e}} \mu_-(q, N_1, e_1) n_1^q p(\mathbf{n},\mathbf{e},t_h) \\
&= -\langle \mu_-(q, N_1, e_1) n_1^q\rangle.
\end{aligned}
$$

Considering the second term of Eq. (S5), we need to consider the cases$j = q$ and $j = q+1$, for $i = 1$. For $i = 1$ and $j = q$, using the change of variables $\bar{n}_1^{q-1} = n_1^{q-1} + 1$ and $\bar{n}_1^q = n_1^q - 1$ we

have

$$
\begin{aligned}
&\sum_{\mathbf{n}}\sum_{\mathbf{e}} n_1^q \mu_+(q-1, N_1, e_1)\left\{(n_1^{q-1}+1)p(D^{\mathrm{p}}_{1,q}\mathbf{n}, \mathbf{e}, t_h) - n_1^{q-1}p(\mathbf{n}, \mathbf{e}, t_h)\right\} \\
&\quad = \sum_{\mathbf{n}}\sum_{\mathbf{e}} n_1^q \mu_+(q-1, N_1, e_1)\times \\
&\qquad \left\{(n_1^{q-1}+1)p([n_1^1, \ldots, n_1^{q-1}+1, n_1^q-1, \ldots, n_1^{N_y+1}], \mathbf{e}, t_h) - n_1^{q-1}p(\mathbf{n}, \mathbf{e}, t_h)\right\} \\
&\quad = \sum_{\mathbf{n}}\sum_{\mathbf{e}} \mu_+(q-1, N_1, e_1)\times \\
&\qquad \left\{\bar{n}_1^{q-1}(\bar{n}_1^q+1)p([n_1^1, \ldots, \bar{n}_1^{q-1}, \bar{n}_1^q, \ldots, n_1^{N_y+1}], \mathbf{e}, t_h) - n_1^q n_1^{q-1}p(\mathbf{n}, \mathbf{e}, t_h)\right\} \\
&\quad = \sum_{\mathbf{n}}\sum_{\mathbf{e}} \mu_+(q-1, N_1, e_1)\left\{n_1^{q-1}(n_1^q+1)p(\mathbf{n}, \mathbf{e}, t_h) - n_1^q n_1^{q-1}p(\mathbf{n}, \mathbf{e}, t_h)\right\} \\
&\quad = \sum_{\mathbf{n}}\sum_{\mathbf{e}} \mu_+(q-1, N_1, e_1)n_1^{q-1}p(\mathbf{n}, \mathbf{e}, t_h) \\
&\quad = \langle \mu_+(q-1, N_1, e_1)n_1^{q-1}\rangle.
\end{aligned}
$$

For the case $j = q+1$ and $i = 1$, the change of variables $\bar{n}_1^{q+1} = n_1^{q+1} - 1$ and $\bar{n}_1^q = n_1^q + 1$ gives

$$
\begin{aligned}
&\sum_{\mathbf{n}}\sum_{\mathbf{e}} n_1^q \mu_+(q, N_1, e_1)\left\{(n_1^q+1)p(D^{\mathrm{p}}_{1,q}\mathbf{n}, \mathbf{e}, t_h) - n_1^q p(\mathbf{n}, \mathbf{e}, t_h)\right\} \\
&\quad = \sum_{\mathbf{n}}\sum_{\mathbf{e}} n_1^q \mu_+(q, N_1, e_1)\times \\
&\qquad \left\{(n_1^q+1)p([n_1^1, \ldots, n_1^q+1, n_1^{q+1}-1, \ldots, n_1^{N_y+1}], \mathbf{e}, t_h) - n_1^q p(\mathbf{n}, \mathbf{e}, t_h)\right\} \\
&\quad = \sum_{\mathbf{n}}\sum_{\mathbf{e}} \mu_+(q, N_1, e_1)\times \\
&\qquad \left\{\bar{n}_1^q(\bar{n}_1^q-1)p([n_1^1, \ldots, \bar{n}_1^q, \bar{n}_1^{q+1}, \ldots, n_1^{N_y+1}], \mathbf{e}, t_h) - n_1^q n_1^q p(\mathbf{n}, \mathbf{e}, t_h)\right\} \\
&\quad = \sum_{\mathbf{n}}\sum_{\mathbf{e}} \mu_+(q, N_1, e_1)\left\{n_1^q(n_1^q-1)p(\mathbf{n}, \mathbf{e}, t_h) - n_1^q n_1^q p(\mathbf{n}, \mathbf{e}, t_h)\right\} \\
&\quad = -\sum_{\mathbf{n}}\sum_{\mathbf{e}} \mu_+(q, N_1, e_1)n_1^q p(\mathbf{n}, \mathbf{e}, t_h) \\
&\quad = -\langle \mu_+(q, N_1, e_1)n_1^q\rangle.
\end{aligned}
$$

Now, looking at the third term of Eq. (S5), which governs movement in physical space, for $j = q$ we have

$$\sum_{\mathbf{n}}\sum_{\mathbf{e}} n_1^q \sum_{i=1}^{N_x} \beta_-(q, N_i, e_i) \left\{(n_{i+1}^q + 1)p(R_{i,q}^{\mathrm{m}}\mathbf{n}, \mathbf{e}, t_h) - n_{i+1}^q p(\mathbf{n}, \mathbf{e}, t_h)\right\}$$

$$= \sum_{\mathbf{n}}\sum_{\mathbf{e}} n_1^q \sum_{i=1}^{N_x} \beta_-(q, N_i, e_i)\times$$

$$\left\{(n_{i+1}^q + 1)p([n_1^q, \ldots, n_i^q - 1, n_{i+1}^q + 1, \ldots, n_{N_x+1}^q], \mathbf{e}, t_h) - n_{i+1}^q p(\mathbf{n}, \mathbf{e}, t_h)\right\},$$

which only produces non-zero contributions when $i = 1$, namely,

$$\sum_{\mathbf{n}}\sum_{\mathbf{e}} n_1^q \beta_-(q, N_1, e_1) \left\{(n_2^q + 1)p([n_1^q - 1, n_2^q + 1, \ldots, n_{N_x+1}^q], \mathbf{e}, t_h) - n_2^q p(\mathbf{n}, \mathbf{e}, t_h)\right\}$$

$$= \sum_{\mathbf{n}}\sum_{\mathbf{e}} \beta_-(q, N_1, e_1) \left\{(\bar{n}_1^q + 1)\bar{n}_2^q p([\bar{n}_1^q, \bar{n}_2^q, \ldots, n_{N_x+1}^q], \mathbf{e}, t_h) - n_1^q n_2^q p(\mathbf{n}, \mathbf{e}, t_h)\right\}$$

$$= \sum_{\mathbf{n}}\sum_{\mathbf{e}} \beta_-(q, N_1, e_1) \{(n_1^q + 1)n_2^q p(\mathbf{n}, \mathbf{e}, t_h) - n_1^q n_2^q p(\mathbf{n}, \mathbf{e}, t_h)\}$$

$$= \sum_{\mathbf{n}}\sum_{\mathbf{e}} \beta_-(q, N_1, e_1) n_2^q p(\mathbf{n}, \mathbf{e}, t_h)$$

$$= \langle \beta_-(q, N_1, e_1) n_2^q \rangle,$$

where we used the change of variables $\bar{n}_1^q = n_1^q - 1$ and $\bar{n}_2^q = n_2^q + 1$, and then dropped the bar. Next, this argument can be repeated for $j = q$ and $i = 2$ in the fourth term of Eq. (S5) (chosen such that $i - 1 = 1$, and recalling that terms with $i > 2$ will give non-zero contributions), using the change of variable $\bar{n}_1^q = n_1^q + 1$ and $\bar{n}_2^q = n_2^q - 1$:

$$\sum_{\mathbf{n}}\sum_{\mathbf{e}} n_1^q \beta_+(q, N_2, e_2) \left\{(n_1^q + 1)p(L_{2,q}^{\mathrm{m}}\mathbf{n}, \mathbf{e}, t_h) - n_1^q p(\mathbf{n}, \mathbf{e}, t_h)\right\}$$

$$= \sum_{\mathbf{n}}\sum_{\mathbf{e}} n_1^q \beta_+(q, N_2, e_2)\times$$

$$\left\{(n_1^q + 1)p([n_1^q + 1, n_2^q - 1, \ldots, n_{N_x+1}^q], \mathbf{e}, t_h) - n_1^q p(\mathbf{n}, \mathbf{e}, t_h)\right\}$$

$$= \sum_{\mathbf{n}}\sum_{\mathbf{e}} \beta_+(q, N_2, e_2) \left\{\bar{n}_1^q(\bar{n}_1^q - 1)p([\bar{n}_1^q, \bar{n}_2^q, \ldots, n_{N_x+1}^q], \mathbf{e}, t_h) - n_1^q n_1^q p(\mathbf{n}, \mathbf{e}, t_h)\right\}$$

$$= \sum_{\mathbf{n}}\sum_{\mathbf{e}} \beta_+(q, N_2, e_2) \{n_1^q(n_1^q - 1)p(\mathbf{n}, \mathbf{e}, t_h) - n_1^q n_1^q p(\mathbf{n}, \mathbf{e}, t_h)\}$$

$$= -\sum_{\mathbf{n}}\sum_{\mathbf{e}} \beta_+(q, N_2, e_2) n_1^q p(\mathbf{n}, \mathbf{e}, t_h)$$

$$= -\langle \beta_+(q, N_2, e_2) n_1^q \rangle.$$

Now, finally, we look at the last term on the right-hand side of Eq. (S5) which has only non-zero contributions when $j = q$ and $i = 1$:

$$\sum_{\mathbf{n}} \sum_{\mathbf{e}} n_1^q \left\{ \gamma(q, N_1 - 1, e_1)(n_1^q - 1) p(G_{1,q}\mathbf{n}, \mathbf{e}, t_h) - n_1^q \gamma(q, N_1, e_1) p(\mathbf{n}, \mathbf{e}, t_h) \right\}$$
$$= \sum_{\mathbf{n}} \sum_{\mathbf{e}} n_1^q \left\{ \gamma(q, N_1 - 1, e_1)(n_1^q - 1) p([n_1^q - 1, \ldots, n_{N_x+1}^q], \mathbf{e}, t_h) - n_1^q \gamma(q, N_1, e_1) p(\mathbf{n}, \mathbf{e}, t_h) \right\}. \tag{S7}$$

Using the change of variable $\bar{n}_1^q = n_1^q - 1$ in the second term of Eq. (S7) we get

$$\begin{aligned}
&\sum_{\mathbf{n}} \sum_{\mathbf{e}} \left\{ \bar{n}_1^q (\bar{n}_1^q + 1) \gamma(q, \bar{N}_1, e_1) \bar{n}_1^q p([\bar{n}_1^q, \ldots, n_{N_x+1}^q], \mathbf{e}, t_h) - (n_1^q)^2 \gamma(q, N_1, e_1) p(\mathbf{n}, \mathbf{e}, t_h) \right\} \\
&= \sum_{\mathbf{n}} \sum_{\mathbf{e}} \left\{ n_1^q (n_1^q + 1) \gamma(q, N_1, e_1) n_1^q p(\mathbf{n}, \mathbf{e}, t_h) - (n_1^q)^2 \gamma(q, N_1, e_1) p(\mathbf{n}, \mathbf{e}, t_h) \right\} \\
&= \sum_{\mathbf{n}} \sum_{\mathbf{e}} \gamma(q, N_1, e_1) n_1^q p(\mathbf{n}, \mathbf{e}, t_h) \\
&= \langle \gamma(q, N_1, e_1) n_1^q \rangle.
\end{aligned}$$

Recompiling these simplified terms, the equation for cell evolution on the left-hand boundary in physical space becomes:

$$\begin{aligned}
\frac{\partial}{\partial t} \langle n_1^q \rangle = &\frac{1}{\Delta_t} \langle \mu_-(q+1, N_1, e_1) n_1^{q+1} \rangle + \frac{1}{\Delta_t} \langle \mu_+(q-1, N_1, e_1) n_1^{q-1} \rangle \\
&- \frac{1}{\Delta_t} \langle \mu_-(q, N_1, e_1) n_1^q \rangle - \frac{1}{\Delta_t} \langle \mu_+(q, N_1, e_1) n_1^q \rangle \\
&+ \frac{1}{\Delta_t} \langle \beta_-(q, N_1, e_1) n_2^q \rangle - \frac{1}{\Delta_t} \langle \beta_+(q, N_2, e_2) n_1^q \rangle + \frac{1}{\Delta_t} \langle \gamma(q, N_1, e_1) n_1^q \rangle.
\end{aligned} \tag{S8}$$

Now we wish to find the continuum equivalent of this equation. Recalling the continuum equivalents of the dependent variables, and employing Taylor series expansions around $x = X_{\min}$, we can rewrite Eq. (S8) as (dropping the dependent variables for simplicity)

$$\begin{aligned}
\Delta_t \frac{\partial n}{\partial t} = &\left(\mu_- + \Delta_y \frac{\partial \mu_-}{\partial y} + \frac{\Delta_y^2}{2} \frac{\partial^2 \mu_-}{\partial y^2} + \ldots \right) \left(n + \Delta_y \frac{\partial n}{\partial y} + \frac{\Delta_y^2}{2} \frac{\partial^2 n}{\partial y^2} + \ldots \right) \\
&+ \left(\mu_+ - \Delta_y \frac{\partial \mu_+}{\partial y} + \frac{\Delta_y^2}{2} \frac{\partial^2 \mu_+}{\partial y^2} + \ldots \right) \left(n - \Delta_y \frac{\partial n}{\partial y} + \frac{\Delta_y^2}{2} \frac{\partial^2 n}{\partial y^2} + \ldots \right) \\
&- \mu_+ n - \mu_- n + \gamma n + \beta_- \left(n + \Delta_x \frac{\partial n}{\partial x} + \frac{\Delta_x^2}{2} \frac{\partial^2 n}{\partial x^2} + \ldots \right) \\
&- n \left(\beta_+ + \Delta_x \frac{\partial \beta_+}{\partial x} + \frac{\Delta_x^2}{2} \frac{\partial^2 \beta_+}{\partial x^2} + \ldots \right),
\end{aligned}$$

at $x = X_{\min}$, so that we have

$$\frac{\partial n}{\partial t} = \frac{\Delta_y}{\Delta_t}\frac{\partial}{\partial y}\left((\mu_- - \mu_+)\, n\right) + \frac{\Delta_y^2}{2\Delta_t}\frac{\partial^2}{\partial y^2}\left((\mu_- + \mu_+)\, n\right) + \frac{1}{\Delta_t} n\left(\beta_- - \beta_+\right)$$
$$+ \frac{\Delta_x}{\Delta_t}\left(\beta_- \frac{\partial n}{\partial x} - n\frac{\partial \beta_+}{\partial x}\right) + \frac{\Delta_x^2}{2\Delta_t}\left(\beta_- \frac{\partial^2 n}{\partial x^2} - n\frac{\partial^2 \beta_+}{\partial x^2}\right) + \frac{1}{\Delta_t}\gamma n. \tag{S9}$$

Recalling that

$$\lim_{\Delta_x, \Delta_t \to 0} \frac{\Delta_x}{\Delta_t}\Big(\beta_-(y,\rho(x,t),e(x,t)) - \beta_+(y,\rho(x,t),e(x,t))\Big) = v^m(y,\rho(x,t),e(x,t)),$$
$$\lim_{\Delta_x, \Delta_t \to 0} \frac{\Delta_x^2}{2\Delta_t}\Big(\beta_-(y,\rho(x,t),e(x,t)) + \beta_+(y,\rho(x,t),e(x,t))\Big) = D^m(y,\rho(x,t),e(x,t)),$$
$$\lim_{\Delta_y, \Delta_t \to 0} \frac{\Delta_y}{\Delta_t}\Big(\mu_-\left(y,\rho(x,t),e(x,t)\right) - \mu_+\left(y,\rho(x,t),e(x,t)\right)\Big) = v^p(y,\rho(x,t),e(x,t)),$$
$$\lim_{\Delta_y, \Delta_t \to 0} \frac{\Delta_y^2}{2\Delta_t}\Big(\mu_-\left(y,\rho(x,t),e(x,t)\right) + \mu_+\left(y,\rho(x,t),e(x,t)\right)\Big) = D^p(y,\rho(x,t),e(x,t)),$$
$$\lim_{\Delta_t \to 0} \frac{1}{\Delta_t}\gamma\left(y,\rho(x,t),e(x,t)\right) = r(y,\rho(x,t),e(x,t)),$$

such that

$$\beta_\pm = \frac{D^m \Delta_t}{\Delta_x^2} \mp \frac{v^m \Delta_t}{2\Delta_x},$$

then Eq. (S9) can be rewritten as

$$\frac{\partial n}{\partial t} = \frac{\partial}{\partial y}\left(v^p n\right) + \frac{\partial^2}{\partial y^2}\left(D^p n\right) + \frac{1}{\Delta_x} v^m n + \frac{1}{2} v^m \frac{\partial n}{\partial x} + \frac{1}{\Delta_x} D^m \frac{\partial n}{\partial x}$$
$$- \frac{1}{\Delta_x} n \frac{\partial}{\partial x} D^m - \frac{1}{2} n \frac{\partial}{\partial x} v^m + \frac{1}{2} D^m \frac{\partial^2 n}{\partial x^2} - \frac{1}{2} n \frac{\partial^2}{\partial x^2} D^m + rn.$$

In order to prevent blow-up of terms in the limit $\Delta_x \to 0$, we require

$$v^m n + D^m \frac{\partial n}{\partial x} - n \frac{\partial}{\partial x} D^m = 0 \qquad \text{at} \quad x = X_{\min}.$$

As such, we have no flux of cells out of the physical space boundary at $x = X_{\min}$.

Boundary condition at $x = X_{\max}$. Revisiting Eq. (S1) to find the equation for the right-hand lattice site in physical space we multiply by $n^q_{N_x+1}$ and sum over all possible states $\mathbf{n}$ and

e:

$$
\begin{aligned}
\Delta_t \sum_{\mathbf{n}} \sum_{\mathbf{e}} n^q_{N_x+1} \frac{\partial}{\partial t} p(\mathbf{n}, \mathbf{e}, t_h) = \\
\sum_{\mathbf{n}} \sum_{\mathbf{e}} n^q_{N_x+1} \sum_{i=1}^{N_x+1} \sum_{j=1}^{N_y} \mu_-(j+1, N_i, e_i) \left\{ (n_i^{j+1}+1) p(U^{\mathrm{p}}_{i,j}\mathbf{n}, \mathbf{e}, t_h) - n_i^{j+1} p(\mathbf{n}, \mathbf{e}, t_h) \right\} \\
+ \sum_{\mathbf{n}} \sum_{\mathbf{e}} n^q_{N_x+1} \sum_{i=1}^{N_x+1} \sum_{j=2}^{N_y+1} \mu_+(j-1, N_i, e_i) \left\{ (n_i^{j-1}+1) p(D^{\mathrm{p}}_{i,j}\mathbf{n}, \mathbf{e}, t_h) - n_i^{j-1} p(\mathbf{n}, \mathbf{e}, t_h) \right\} \\
+ \sum_{\mathbf{n}} \sum_{\mathbf{e}} n^q_{N_x+1} \sum_{i=1}^{N_x} \sum_{j=1}^{N_y+1} \beta_-(j, N_i, e_i) \left\{ (n_{i+1}^{j}+1) p(R^{\mathrm{m}}_{i,j}\mathbf{n}, \mathbf{e}, t_h) - n_{i+1}^{j} p(\mathbf{n}, \mathbf{e}, t_h) \right\} \\
+ \sum_{\mathbf{n}} \sum_{\mathbf{e}} n^q_{N_x+1} \sum_{i=2}^{N_x+1} \sum_{j=1}^{N_y+1} \beta_+(j, N_i, e_i) \left\{ (n_{i-1}^{j}+1) p(L^{\mathrm{m}}_{i,j}\mathbf{n}, \mathbf{e}, t_h) - n_{i-1}^{j} p(\mathbf{n}, \mathbf{e}, t_h) \right\} \\
+ \sum_{\mathbf{n}} \sum_{\mathbf{e}} n^q_{N_x+1} \sum_{i=1}^{N_x+1} \sum_{j=1}^{N_y+1} \left\{ \gamma(j, N_i-1, e_i)(n_i^j - 1) p(G_{i,j}\mathbf{n}, \mathbf{e}, t_h) - \gamma(j, N_i, e_i) n_i^j p(\mathbf{n}, \mathbf{e}, t_h) \right\}.
\end{aligned}
\tag{S10}
$$

Now we can repeat the analysis from the previous section, where we considered the action on the boundary $x = X_{\min}$, for $x = X_{\max}$. In the first two terms on the right-hand side of Eq. (S10), we are interested in the $i = N_x$ terms only. In the first term, we need to consider the cases $j = q$ and $j = q-1$. First, looking at $j = q$ and using the change of variables $\bar{n}^{q+1}_{N_x+1} = n^{q+1}_{N_x+1} + 1$ and $\bar{n}^{q}_{N_x+1} = n^{q}_{N_x+1} - 1$:

$$
\begin{aligned}
&\sum_{\mathbf{n}} \sum_{\mathbf{e}} n^q_{N_x+1} \mu_-(q+1, N_{N_x+1}, e_{N_x+1}) \left\{ (n^{q+1}_{N_x+1}+1) p(U^{\mathrm{p}}_{N_x+1,q}\mathbf{n}, \mathbf{e}, t_h) - n^{q+1}_{N_x+1} p(\mathbf{n}, \mathbf{e}, t_h) \right\} \\
&\quad = \sum_{\mathbf{n}} \sum_{\mathbf{e}} n^q_{N_x+1} \mu_-(q+1, N_{N_x+1}, e_{N_x+1}) \times \\
&\qquad\qquad \left\{ (n^{q+1}_{N_x+1}+1) p([n^1_{N_x+1}, \ldots, n^q_{N_x+1} - 1, n^{q+1}_{N_x+1} + 1, \ldots, n^{N_y+1}_{N_x+1}], \mathbf{e}, t_h) - n^{q+1}_{N_x+1} p(\mathbf{n}, \mathbf{e}, t_h) \right\} \\
&\quad = \sum_{\mathbf{n}} \sum_{\mathbf{e}} \mu_-(q+1, N_{N_x+1}, e_{N_x+1}) \times \\
&\qquad\qquad \left\{ \bar{n}^{q+1}_{N_x+1} (\bar{n}^q_{N_x+1}+1) p([n^1_{N_x+1}, \ldots, \bar{n}^q_{N_x+1}, \bar{n}^{q+1}_{N_x+1}, \ldots, n^{N_y+1}_{N_x+1}], \mathbf{e}, t_h) - n^q_{N_x+1} n^{q+1}_{N_x+1} p(\mathbf{n}, \mathbf{e}, t_h) \right\} \\
&\quad = \sum_{\mathbf{n}} \sum_{\mathbf{e}} \mu_-(q+1, N_{N_x+1}, e_{N_x+1}) \left\{ n^{q+1}_{N_x+1} (n^q_{N_x+1}+1) p(\mathbf{n}, \mathbf{e}, t_h) - n^q_{N_x+1} n^{q+1}_{N_x+1} p(\mathbf{n}, \mathbf{e}, t_h) \right\} \\
&\quad = \sum_{\mathbf{n}} \sum_{\mathbf{e}} \mu_-(q+1, N_{N_x+1}, e_{N_x+1}) n^{q+1}_{N_x+1} p(\mathbf{n}, \mathbf{e}, t_h) \\
&\quad = \langle \mu_-(q+1, N_{N_x+1}, e_{N_x+1}) n^{q+1}_{N_x+1} \rangle.
\end{aligned}
$$

Using $j = q$ and $i = N_x + 1$, with the change of variables $\bar{n}^{q-1}_{N_x+1} = n^{q-1}_{N_x+1} - 1$ and $\bar{n}^{q}_{N_x+1} = n^{q}_{N_x+1} + 1$ in the first term, then we have

$$\begin{aligned}
&\sum_{\mathbf{n}}\sum_{\mathbf{e}} n^q_{N_x+1}\mu_-(q, N_{N_x+1}, e_{N_x+1})\times \\
&\qquad\Big\{(n^q_{N_x+1}+1)p([n^1_{N_x+1},\ldots,n^{q-1}_{N_x+1}-1, n^q_{N_x+1}+1,\ldots,n^{N_y+1}_{N_x+1}],\mathbf{e},t_h) - n^q_{N_x+1}p(\mathbf{n},\mathbf{e},t_h)\Big\} \\
&\quad= \sum_{\mathbf{n}}\sum_{\mathbf{e}} \mu_-(q, N_{N_x+1}, e_{N_x+1})\times \\
&\qquad\Big\{\bar{n}^q_{N_x+1}(\bar{n}^q_{N_x+1}-1)p([n^1_{N_x+1},\ldots,\bar{n}^{q-1}_{N_x+1}, \bar{n}^q_{N_x+1},\ldots,n^{N_y+1}_{N_x+1}],\mathbf{e},t_h) - n^q_{N_x+1}n^q_{N_x+1}p(\mathbf{n},\mathbf{e},t_h)\Big\} \\
&\quad= \sum_{\mathbf{n}}\sum_{\mathbf{e}} \mu_-(q, N_{N_x+1}, e_{N_x+1})\left\{n^q_{N_x+1}(n^q_{N_x+1}-1)p(\mathbf{n},\mathbf{e},t_h) - n^q_{N_x+1}n^q_{N_x+1}p(\mathbf{n},\mathbf{e},t_h)\right\} \\
&\quad= -\sum_{\mathbf{n}}\sum_{\mathbf{e}} \mu_-(q, N_{N_x+1}, e_{N_x+1})n^q_{N_x+1}p(\mathbf{n},\mathbf{e},t_h) \\
&\quad= -\langle \mu_-(q, N_{N_x+1}, e_{N_x+1})n^q_{N_x+1}\rangle.
\end{aligned}$$

For the second term, we need to consider $j = q$ and $j = q + 1$ whilst maintaining $i = N_x + 1$. For $i = N_x + 1$ and $j = q$, whilst using the change of variables $\bar{n}^{q-1}_{N_x+1} = n^{q-1}_{N_x+1} + 1$ and $\bar{n}^{q}_{N_x+1} = n^{q}_{N_x+1} - 1$ we have

$$\begin{aligned}
&\sum_{\mathbf{n}}\sum_{\mathbf{e}} n^q_{N_x+1}\mu_+(q-1, N_{N_x+1}, e_{N_x+1})\Big\{(n^{q-1}_{N_x+1}+1)p(D^{\mathrm{p}}_{N_x+1,q}\mathbf{n},\mathbf{e},t_h) - n^{q-1}_{N_x+1}p(\mathbf{n},\mathbf{e},t_h)\Big\} \\
&\quad= \sum_{\mathbf{n}}\sum_{\mathbf{e}} n^q_{N_x+1}\mu_+(q-1, N_{N_x+1}, e_{N_x+1})\times \\
&\qquad\Big\{(n^{q-1}_{N_x+1}+1)p([n^1_{N_x+1},\ldots,n^{q-1}_{N_x+1}+1, n^q_{N_x+1}-1,\ldots,n^{N_y+1}_{N_x+1}],\mathbf{e},t_h) - n^{q-1}_{N_x+1}p(\mathbf{n},\mathbf{e},t_h)\Big\} \\
&\quad= \sum_{\mathbf{n}}\sum_{\mathbf{e}} \mu_+(q-1, N_{N_x+1}, e_{N_x+1})\times \\
&\qquad\Big\{\bar{n}^{q-1}_{N_x+1}(\bar{n}^q_{N_x+1}+1)p([n^1_{N_x+1},\ldots,\bar{n}^{q-1}_{N_x+1}, \bar{n}^q_{N_x+1},\ldots,n^{N_y+1}_{N_x+1}],\mathbf{e},t_h) - n^q_{N_x+1}n^{q-1}_{N_x+1}p(\mathbf{n},\mathbf{e},t_h)\Big\} \\
&\quad= \sum_{\mathbf{n}}\sum_{\mathbf{e}} \mu_+(q-1, N_{N_x+1}, e_{N_x+1})\Big\{n^{q-1}_{N_x+1}(n^q_{N_x+1}+1)p(\mathbf{n},\mathbf{e},t_h) - n^q_{N_x+1}n^{q-1}_{N_x+1}p(\mathbf{n},\mathbf{e},t_h)\Big\} \\
&\quad= \sum_{\mathbf{n}}\sum_{\mathbf{e}} \mu_+(q-1, N_{N_x+1}, e_{N_x+1})n^{q-1}_{N_x+1}p(\mathbf{n},\mathbf{e},t_h) \\
&\quad= \langle \mu_+(q-1, N_{N_x+1}, e_{N_x+1})n^{q-1}_{N_x+1}\rangle.
\end{aligned}$$

Now looking at $j = q + 1$ and $i = N_x$, the change of variables $\bar{n}^{q+1}_{N_x+1} = n^{q+1}_{N_x+1} - 1$ and $\bar{n}^{q}_{N_x+1} = n^{q}_{N_x+1} + 1$ gives

$$
\begin{aligned}
&\sum_{\mathbf{n}}\sum_{\mathbf{e}} n^q_{N_x+1}\mu_+(q, N_{N_x+1}, e_{N_x+1}) \Big\{(n^q_{N_x+1}+1)p(D^{\mathrm{p}}_{N_x+1,q}\mathbf{n}, \mathbf{e}, t_h) - n^q_{N_x+1}p(\mathbf{n}, \mathbf{e}, t_h)\Big\} \\
&\quad = \sum_{\mathbf{n}}\sum_{\mathbf{e}} n^q_{N_x+1}\mu_+(q, N_{N_x+1}, e_{N_x+1})\times \\
&\qquad\qquad \Big\{(n^q_{N_x+1}+1)p([n^1_{N_x+1}, \dots, n^q_{N_x+1}+1, n^{q+1}_{N_x+1}-1, \dots, n^{N_y+1}_{N_x+1}], \mathbf{e}, t_h) - n^q_{N_x+1}p(\mathbf{n}, \mathbf{e}, t_h)\Big\} \\
&\quad = \sum_{\mathbf{n}}\sum_{\mathbf{e}} \mu_+(q, N_{N_x+1}, e_{N_x+1})\times \\
&\qquad\qquad \Big\{\bar{n}^q_{N_x+1}(\bar{n}^q_{N_x+1}-1)p([n^1_{N_x+1}, \dots, \bar{n}^q_{N_x+1}, \bar{n}^{q+1}_{N_x+1}, \dots, n^{N_y+1}_{N_x+1}], \mathbf{e}, t_h) - n^q_{N_x+1}n^q_{N_x+1}p(\mathbf{n}, \mathbf{e}, t_h)\Big\} \\
&\quad = \sum_{\mathbf{n}}\sum_{\mathbf{e}} \mu_+(q, N_{N_x+1}, e_{N_x+1}) \left\{n^q_{N_x+1}(n^q_{N_x+1}-1)p(\mathbf{n}, \mathbf{e}, t_h) - n^q_{N_x+1}n^q_{N_x+1}p(\mathbf{n}, \mathbf{e}, t_h)\right\} \\
&\quad = -\sum_{\mathbf{n}}\sum_{\mathbf{e}} \mu_+(q, N_{N_x+1}, e_{N_x+1})n^q_{N_x+1}p(\mathbf{n}, \mathbf{e}, t_h) \\
&\quad = -\langle \mu_+(q, N_{N_x+1}, e_{N_x+1})n^q_{N_x+1}\rangle.
\end{aligned}
$$

Next, we want to consider the movement terms in physical space. In these cases, we will only have non-zero contributions when $j = q$. Looking at the first term, we will have contributions only when $i = N_x$. Then, using the change of variables $\bar{n}^q_{N_x+1} = n^q_{N_x+1} + 1$ and $\bar{n}^q_{N_x} = n^q_{N_x} - 1$

and dropping the bars, we get

$$
\begin{aligned}
&\sum_{\mathbf{n}}\sum_{\mathbf{e}} n^q_{N_x+1}\beta_-(q,N_{N_x},e_{N_x})\left\{(n^q_{N_x+1}+1)p(R^{\mathrm{m}}_{N_x,q}\mathbf{n},\mathbf{e},t_h)-n^q_{N_x+1}p(\mathbf{n},\mathbf{e},t_h)\right\}\\
&\quad=\sum_{\mathbf{n}}\sum_{\mathbf{e}} n^q_{N_x+1}\beta_-(q,N_{N_x},e_{N_x})\times\\
&\qquad\qquad\left\{(n^q_{N_x+1}+1)p([n^q_1,\ldots,n^q_{N_x}-1,n^q_{N_x+1}+1],\mathbf{e},t_h)-n^q_{N_x+1}p(\mathbf{n},\mathbf{e},t_h)\right\}\\
&\quad=\sum_{\mathbf{n}}\sum_{\mathbf{e}} n^q_{N_x}\beta_-(q,N_{N_x},e_{N_x})\times\\
&\qquad\qquad\left\{(n^q_{N_x+1}+1)p([n^q_1,\ldots,n^q_{N_x}-1,n^q_{N_x+1}+1],\mathbf{e},t_h)-n^q_{N_x+1}p(\mathbf{n},\mathbf{e},t_h)\right\},\\
&\quad=\sum_{\mathbf{n}}\sum_{\mathbf{e}} \beta_-(q,N_{N_x},e_{N_x})\times\\
&\qquad\qquad\left\{(\bar{n}^q_{N_x}-1)\bar{n}^q_{N_x+1}p([n^q_1,\ldots,\bar{n}^q_{N_x},\bar{n}^q_{N_x+1}],\mathbf{e},t_h)-n^q_{N_x}n^q_{N_x+1}p(\mathbf{n},\mathbf{e},t_h)\right\}\\
&\quad=\sum_{\mathbf{n}}\sum_{\mathbf{e}} \beta_-(q,N_{N_x},e_{N_x})\times\\
&\qquad\qquad\left\{(n^q_{N_x}-1)n^q_{N_x+1}p(\mathbf{n},\mathbf{e},t_h)-n^q_{N_x}n^q_{N_x+1}p(\mathbf{n},\mathbf{e},t_h)\right\}\\
&\quad=-\sum_{\mathbf{n}}\sum_{\mathbf{e}} \beta_-(q,N_{N_x},e_{N_x})n^q_{N_x+1}p(\mathbf{n},\mathbf{e},t_h)\\
&\quad=-\langle\beta_-(q,N_{N_x},e_{N_x})n^q_{N_x+1}\rangle,
\end{aligned}
$$

Now we can repeat this for $j=q$ and $i=N_x+1$ in the fourth term of Eq. (S10), using the change of variable $\bar{n}^q_{N_x+1}=n^q_{N_x+1}-1$ and $\bar{n}^q_{N_x}=n^q_{N_x}+1$:

$$
\begin{aligned}
&\sum_{\mathbf{n}}\sum_{\mathbf{e}} n^q_{N_x+1}\beta_+(q,N_{N_x+1},e_{N_x+1})\left\{(n^q_{N_x}+1)p(L^{\mathrm{m}}_{N_x,q}\mathbf{n},\mathbf{e},t_h)-n^q_{N_x}p(\mathbf{n},\mathbf{e},t_h)\right\},\\
&\quad=\sum_{\mathbf{n}}\sum_{\mathbf{e}} n^q_{N_x+1}\beta_+(q,N_{N_x+1},e_{N_x+1})\times\\
&\qquad\qquad\left\{(n^q_{N_x}+1)p([n^q_1,\ldots,n^q_{N_x}+1,n^q_{N_x+1}-1],\mathbf{e},t_h)-n^q_{N_x}p(\mathbf{n},\mathbf{e},t_h)\right\}\\
&\quad=\sum_{\mathbf{n}}\sum_{\mathbf{e}} \beta_+(q,N_{N_x+1},e_{N_x+1})\times\\
&\qquad\qquad\left\{\bar{n}^q_{N_x}(\bar{n}^q_{N_x+1}+1)p([n^q_1,\ldots,\bar{n}^q_{N_x},\bar{n}^q_{N_x+1}],\mathbf{e},t_h)-n^q_{N_x+1}n^q_{N_x}p(\mathbf{n},\mathbf{e},t_h)\right\}\\
&\quad=\sum_{\mathbf{n}}\sum_{\mathbf{e}} \beta_+(q,N_{N_x+1},e_{N_x+1})\left\{n^q_{N_x}(n^q_{N_x+1}+1)p(\mathbf{n},\mathbf{e},t_h)-n^q_{N_x}n^q_{N_x+1}p(\mathbf{n},\mathbf{e},t_h)\right\}\\
&\quad=\sum_{\mathbf{n}}\sum_{\mathbf{e}} \beta_+(q,N_{N_x+1},e_{N_x+1})n^q_{N_x}p(\mathbf{n},\mathbf{e},t_h)\\
&\quad=\langle\beta_+(q,N_{N_x+1},e_{N_x+1})n^q_{N_x}\rangle.
\end{aligned}
$$

The final term on right-hand side of Eq. (S10) has non-zero contributions when $j=q$ and $i=N_x+1$ only. We employ the change of variable $\bar{n}^q_{N_x+1}=n^q_{N_x+1}-1$ in the second term to

get

$$
\begin{aligned}
&\sum_{\mathbf{n}}\sum_{\mathbf{e}} n^q_{N_x+1}\left\{\gamma(q, N_{N_x+1}-1, e_{N_x+1})(n^q_{N_x+1}-1)p(G_{N_x+1,q}\mathbf{n}, \mathbf{e}, t_h) - n^q_{N_x+1}\gamma(q, N_{N_x+1}, e_{N_x+1})p(\mathbf{n}, \mathbf{e}, t_h)\right\} \\
&\quad = \sum_{\mathbf{n}}\sum_{\mathbf{e}} n^q_{N_x+1}\big\{\gamma(q, N_{N_x+1}-1, e_{N_x+1})(n^q_{N_x+1}-1)p([n^q_1, \dots, n^q_{N_x+1}-1], \mathbf{e}, t_h) \\
&\qquad\qquad\qquad - n^q_{N_x+1}\gamma(q, N_{N_x+1}, e_{N_x+1})p(\mathbf{n}, \mathbf{e}, t_h)\big\} \\
&\quad = \sum_{\mathbf{n}}\sum_{\mathbf{e}} \big\{\gamma(q, \bar{N}_{N_x+1}, e_{N_x+1})(\bar{n}^q_{N_x+1}-1)\bar{n}^q_{N_x+1}p([n^q_1, \dots, \bar{n}^q_{N_x+1}], \mathbf{e}, t_h) \\
&\qquad\qquad\qquad - \gamma(q, N_{N_x+1}, e_{N_x+1})(n^q_{N_x+1})^2 p(\mathbf{n}, \mathbf{e}, t_h)\big\} \\
&\quad = \sum_{\mathbf{n}}\sum_{\mathbf{e}} \gamma(q, N_{N_x+1}, e_{N_x+1})\left\{(n^q_{N_x+1}-1)n^q_{N_x+1}p(\mathbf{n}, \mathbf{e}, t_h) - (n^q_{N_x+1})^2 p(\mathbf{n}, \mathbf{e}, t_h)\right\} \\
&\quad = \sum_{\mathbf{n}}\sum_{\mathbf{e}} \gamma(q, N_{N_x+1}, e_{N_x+1})n^q_{N_x+1}p(\mathbf{n}, \mathbf{e}, t_h) \\
&\quad = \langle \gamma(q, N_{N_x+1}, e_{N_x+1})n^q_{N_x+1}\rangle.
\end{aligned}
$$

Putting these together, the equation for cell evolution on the right-hand boundary in physical space becomes:

$$
\begin{aligned}
\frac{\partial}{\partial t}\langle n^q_{N_x+1}\rangle &= \frac{1}{\Delta_t}\langle \mu_-(q+1, N_{N_x+1}, e_{N_x+1})n^{q+1}_{N_x+1}\rangle + \frac{1}{\Delta_t}\langle \mu_+(q-1, N_{N_x+1}, e_{N_x+1})n^{q-1}_{N_x+1}\rangle \\
&- \frac{1}{\Delta_t}\langle \mu_-(q, N_{N_x+1}, e_{N_x+1})n^q_{N_x+1}\rangle - \frac{1}{\Delta_t}\langle \mu_+(q, N_{N_x+1}, e_{N_x+1})n^q_{N_x+1}\rangle \\
&+ \frac{1}{\Delta_t}\langle \beta_+(q, N_{N_x+1}, e_{N_x+1})n^q_{N_x}\rangle - \frac{1}{\Delta_t}\langle \beta_-(q, N_{N_x}, e_{N_x})n^q_{N_x+1}\rangle \\
&+ \frac{1}{\Delta_t}\langle \gamma(q, N_{N_x+1}, e_{N_x+1})n^q_{N_x+1}\rangle. \qquad \text{(S11)}
\end{aligned}
$$

Now we want to find the continuum equivalent of this equation. Recalling the continuum equivalents of the dependent variables whilst employing Taylor series expansions around $x = X_{\max}$, we can rewrite Eq. (S11) as (dropping the dependent variables for simplicity)

$$
\begin{aligned}
\Delta_t\frac{\partial n}{\partial t} &= \left(\mu_- + \Delta_y\frac{\partial \mu_-}{\partial y} + \frac{\Delta_y^2}{2}\frac{\partial^2 \mu_-}{\partial y^2} + \dots\right)\left(n + \Delta_y\frac{\partial n}{\partial y} + \frac{\Delta_y^2}{2}\frac{\partial^2 n}{\partial y^2} + \dots\right) \\
&+ \left(\mu_+ - \Delta_y\frac{\partial \mu_+}{\partial y} + \frac{\Delta_y^2}{2}\frac{\partial^2 \mu_+}{\partial y^2} + \dots\right)\left(n - \Delta_y\frac{\partial n}{\partial y} + \frac{\Delta_y^2}{2}\frac{\partial^2 n}{\partial y^2} + \dots\right) \\
&- \mu_+ n - \mu_- n + \gamma n + \beta_+\left(n - \Delta_x\frac{\partial n}{\partial x} + \frac{\Delta_x^2}{2}\frac{\partial^2 n}{\partial x^2} + \dots\right) \\
&- n\left(\beta_- - \Delta_x\frac{\partial \beta_-}{\partial x} + \frac{\Delta_x^2}{2}\frac{\partial^2 \beta_-}{\partial x^2} + \dots\right),
\end{aligned}
$$

at $x = X_{\max}$ so that

$$\frac{\partial n}{\partial t} = \frac{\Delta_y}{\Delta_t}\frac{\partial}{\partial y}\left(\left(\mu_- - \mu_+\right) n\right) + \frac{\Delta_y^2}{2\Delta_t}\frac{\partial^2}{\partial y^2}\left(\left(\mu_- + \mu_+\right) n\right) - \frac{1}{\Delta_t} n\left(\beta_- - \beta_+\right)$$
$$- \frac{\Delta_x}{\Delta_t}\left(\beta_+\frac{\partial n}{\partial x} - n\frac{\partial \beta_-}{\partial x}\right) + \frac{\Delta_x^2}{2\Delta_t}\left(\beta_+\frac{\partial^2 n}{\partial x^2} - n\frac{\partial^2 \beta_-}{\partial x^2}\right) + \frac{1}{\Delta_t}\gamma n.$$

Recalling that

$$\lim_{\Delta_x, \Delta_t \to 0} \frac{\Delta_x}{\Delta_t}\Big(\beta_-(y, \rho(x,t), e(x,t)) - \beta_+(y, \rho(x,t), e(x,t))\Big) = v^m(y, \rho(x,t), e(x,t)),$$
$$\lim_{\Delta_x, \Delta_t \to 0} \frac{\Delta_x^2}{2\Delta_t}\Big(\beta_-(y, \rho(x,t), e(x,t)) + \beta_+(y, \rho(x,t), e(x,t))\Big) = D^m(y, \rho(x,t), e(x,t)),$$
$$\lim_{\Delta_y, \Delta_t \to 0} \frac{\Delta_y}{\Delta_t}\Big(\mu_-\left(y, \rho(x,t), e(x,t)\right) - \mu_+\left(y, \rho(x,t), e(x,t)\right)\Big) = v^p(y, \rho(x,t), e(x,t)),$$
$$\lim_{\Delta_y, \Delta_t \to 0} \frac{\Delta_y^2}{2\Delta_t}\Big(\mu_-\left(y, \rho(x,t), e(x,t)\right) + \mu_+\left(y, \rho(x,t), e(x,t)\right)\Big) = D^p(y, \rho(x,t), e(x,t)),$$
$$\lim_{\Delta_t \to 0} \frac{1}{\Delta_t}\gamma\left(y, \rho(x,t), e(x,t)\right) = r(y, \rho(x,t), e(x,t)),$$

such that

$$\beta_\pm = \frac{D^m \Delta_t}{\Delta_x^2} \mp \frac{v^m \Delta_t}{2\Delta_x},$$

then the equation can be rewritten as

$$\frac{\partial n}{\partial t} = \frac{\partial}{\partial y}\left(v^p n\right) + \frac{\partial^2}{\partial y^2}\left(D^p n\right) - \frac{1}{\Delta_x} v^m n + \frac{1}{2} v^m \frac{\partial n}{\partial x} - \frac{1}{\Delta_x} D^m \frac{\partial n}{\partial x}$$
$$+ \frac{1}{\Delta_x} n \frac{\partial}{\partial x} D^m - \frac{1}{2} n \frac{\partial}{\partial x} v^m + \frac{1}{2} D^m \frac{\partial^2 n}{\partial x^2} - \frac{1}{2} n \frac{\partial^2}{\partial x^2} D^m + rn.$$

In order to prevent blow-up of terms in the limit $\Delta_x \to 0$, we require

$$-v^m n - D^m \frac{\partial n}{\partial x} + n \frac{\partial}{\partial x} D^m = 0 \qquad \text{at} \quad x = X_{\max}.$$

As such, we have no flux of cells out of the physical space boundary at $x = X_{\max}$.

Boundary condition at $y = Y_{\min}$. Returning to Eq. (S1), we seek an equation for the evolution of the cell number on the left most lattice site in phenotype space, *i.e.*, at y_1. To find

this, we multiply Eq. (S1) by n_s^1 and sum over all possible states $\mathbf{n}$ and $\mathbf{e}$:

$$\begin{aligned}
&\Delta_t \sum_{\mathbf{n}} \sum_{\mathbf{e}} n_s^1 \frac{\partial}{\partial t} p(\mathbf{n}, \mathbf{e}, t_h) + O(\Delta_t^2) \\
&= \sum_{\mathbf{n}} \sum_{\mathbf{e}} n_s^1 \sum_{i=1}^{N_x+1} \sum_{j=1}^{N_y} \mu_-(j+1, N_i, e_i) \times \\
&\qquad\qquad \Big\{ (n_i^{j+1}+1) p(U_{i,j}^{\mathrm{p}} \mathbf{n}, \mathbf{e}, t_h) - n_i^{j+1} p(\mathbf{n}, \mathbf{e}, t_h) \Big\} \\
&+ \sum_{\mathbf{n}} \sum_{\mathbf{e}} n_s^1 \sum_{i=1}^{N_x+1} \sum_{j=2}^{N_y+1} \mu_+(j-1, N_i, e_i) \times \\
&\qquad\qquad \Big\{ (n_i^{j-1}+1) p(D_{i,j}^{\mathrm{p}} \mathbf{n}, \mathbf{e}, t_h) - n_i^{j-1} p(\mathbf{n}, \mathbf{e}, t_h) \Big\} \\
&+ \sum_{\mathbf{n}} \sum_{\mathbf{e}} n_s^1 \sum_{i=1}^{N_x} \sum_{j=1}^{N_y+1} \beta_-(j, N_i, e_i) \Big\{ (n_{i+1}^j+1) p(R_{i,j}^{\mathrm{m}} \mathbf{n}, \mathbf{e}, t_h) - n_{i+1}^j p(\mathbf{n}, \mathbf{e}, t_h) \Big\} \\
&+ \sum_{\mathbf{n}} \sum_{\mathbf{e}} n_s^1 \sum_{i=2}^{N_x+1} \sum_{j=1}^{N_y+1} \beta_+(j, N_i, e_i) \Big\{ (n_{i-1}^j+1) p(L_{i,j}^{\mathrm{m}} \mathbf{n}, \mathbf{e}, t_h) - n_{i-1}^j p(\mathbf{n}, \mathbf{e}, t_h) \Big\} \\
&+ \sum_{\mathbf{n}} \sum_{\mathbf{e}} n_s^1 \sum_{i=1}^{N_x+1} \sum_{j=1}^{N_y+1} \Big\{ \gamma(j, N_i-1, e_i)(n_i^j-1) p(G_{i,j} \mathbf{n}, \mathbf{e}, t_h) - \gamma(j, N_i, e_i) n_i^j p(\mathbf{n}, \mathbf{e}, t_h) \Big\}.
\end{aligned} \tag{S12}$$

Using the same methods as on the boundaries in physical space, we can change variables in each term to find an equation for evolution of cell number. Consider the first term on the right-hand side. The non-zero contributions come from when $j = 1$ and $i = s$. In the second term, the non-zero terms are for $j = 2$ and $i = s$. The third term gives non-zero contributions when $j = 1$ and $i = s$ or $i = s - 1$. In the fourth term, there are non-zero contributions when $j = 1$ and $i = s$ or $i = s + 1$. The final term produces non-zero contributions only when $i = s$ and $j = 1$. Employing this knowledge, Eq. (S12) becomes

$$\begin{aligned}
&\Delta_t \sum_{\mathbf{n}} \sum_{\mathbf{e}} n_s^1 \frac{\partial}{\partial t} p(\mathbf{n}, \mathbf{e}, t_h) + O(\Delta_t^2) \\
&= \sum_{\mathbf{n}} \sum_{\mathbf{e}} n_s^1 \mu_-(2, N_s, e_s) \Big\{ (n_s^2+1) p(U_{s,1}^{\mathrm{p}} \mathbf{n}, \mathbf{e}, t_h) - n_s^2 p(\mathbf{n}, \mathbf{e}, t_h) \Big\} \\
&\quad + \sum_{\mathbf{n}} \sum_{\mathbf{e}} n_s^1 \mu_+(1, N_s, e_s) \Big\{ (n_s^1+1) p(D_{s,2}^{\mathrm{p}} \mathbf{n}, \mathbf{e}, t_h) - n_s^1 p(\mathbf{n}, \mathbf{e}, t_h) \Big\} \\
&\quad + \sum_{\mathbf{n}} \sum_{\mathbf{e}} n_s^1 \sum_{i=s,s-1} \beta_-(1, N_i, e_i) \left\{ (n_{i+1}^1+1) p(R_{i,1}^{\mathrm{m}} \mathbf{n}, \mathbf{e}, t_h) - n_{i+1}^1 p(\mathbf{n}, \mathbf{e}, t_h) \right\} \\
&\quad + \sum_{\mathbf{n}} \sum_{\mathbf{e}} n_s^1 \sum_{i=s,s+1} \beta_+(1, N_i, e_i) \left\{ (n_{i-1}^1+1) p(L_{i,1}^{\mathrm{m}} \mathbf{n}, \mathbf{e}, t_h) - n_{i-1}^1 p(\mathbf{n}, \mathbf{e}, t_h) \right\} \\
&\quad + \sum_{\mathbf{n}} \sum_{\mathbf{e}} n_s^1 \gamma(1, N_s, e_s) \left\{ n_s^1 p(\mathbf{n}, \mathbf{e}, t_h) - (n_s^1+1) p(G_{s,1} \mathbf{n}, \mathbf{e}, t_h) \right\}.
\end{aligned} \tag{S13}$$

The first term can be rewritten using the change of variables $\bar{n}_s^2 = n_s^2 + 1$ and $\bar{n}_s^1 = n_s^1 - 1$

$$\begin{aligned}
&\sum_{\mathbf{n}}\sum_{\mathbf{e}} n_s^1 \mu_-(2, N_s, e_s) \Big\{(n_s^2 + 1)p(U_{s,1}^{\mathrm{p}}\mathbf{n}, \mathbf{e}, t_h) - n_s^{N_y+1} p(\mathbf{n}, \mathbf{e}, t_h)\Big\} \\
&\quad = \sum_{\mathbf{n}}\sum_{\mathbf{e}} n_s^1 \mu_-(2, N_s, e_s) \times \\
&\qquad\qquad \Big\{(n_s^2 + 1)p([n_s^1 - 1, n_s^2 + 1, \dots n_s^{N_y+1}], \mathbf{e}, t_h) - n_s^2 p(\mathbf{n}, \mathbf{e}, t_h)\Big\} \\
&\quad = \sum_{\mathbf{n}}\sum_{\mathbf{e}} \mu_-(2, N_s, e_s) \times \\
&\qquad\qquad \Big\{\bar{n}_s^2(\bar{n}_s^1 + 1)p([\bar{n}_s^1, \bar{n}_s^2, \dots, \bar{n}_s^{N_y+1}], \mathbf{e}, t_h) - n_s^2 n_s^1 p(\mathbf{n}, \mathbf{e}, t_h)\Big\} \\
&\quad = \sum_{\mathbf{n}}\sum_{\mathbf{e}} \mu_-(2, N_s, e_s) \left\{n_s^2(n_s^1 + 1)p(\mathbf{n}, \mathbf{e}, t_h) - n_s^1 n_s^2 p(\mathbf{n}, \mathbf{e}, t_h)\right\} \\
&\quad = \sum_{\mathbf{n}}\sum_{\mathbf{e}} \mu_-(2, N_s, e_s) n_s^2 p(\mathbf{n}, \mathbf{e}, t_h) \\
&\quad = \langle \mu_-(2, N_s, e_s) n_s^2 \rangle.
\end{aligned}$$

The second term in the right-hand side of Eq. (S13) can then be rewritten as the following (using $\bar{n}_s^2 = n_s^2 - 1$ and $\bar{n}_s^1 = n_s^1 + 1$):

$$\begin{aligned}
&\sum_{\mathbf{n}}\sum_{\mathbf{e}} n_s^1 \mu_+(1, N_s, e_s) \Big\{(n_s^1 + 1)p(D_{s,1}^{\mathrm{p}}\mathbf{n}, \mathbf{e}, t_h) - n_s^1 p(\mathbf{n}, \mathbf{e}, t_h)\Big\} \\
&\quad = \sum_{\mathbf{n}}\sum_{\mathbf{e}} n_s^1 \mu_+(1, N_s, e_s) \times \\
&\qquad\qquad \Big\{(n_s^1 + 1)p([n_s^1 + 1, n_s^2 - 1, \dots, n_s^{N_y+1}], \mathbf{e}, t_h) - n_s^1 p(\mathbf{n}, \mathbf{e}, t_h)\Big\} \\
&\quad = \sum_{\mathbf{n}}\sum_{\mathbf{e}} \mu_+(1, N_s, e_s) \times \\
&\qquad\qquad \Big\{\bar{n}_s^1(\bar{n}_s^1 - 1)p([\bar{n}_s^1, \bar{n}_s^2, \dots, n_s^{N_y}], \mathbf{e}, t_h) - n_s^1 n_s^1 p(\mathbf{n}, \mathbf{e}, t_h)\Big\} \\
&\quad = \sum_{\mathbf{n}}\sum_{\mathbf{e}} \mu_+(1, N_s, e_s) \left\{n_s^1(n_s^2 + 1)p(\mathbf{n}, \mathbf{e}, t_h) - n_s^1 n_s^1 p(\mathbf{n}, \mathbf{e}, t_h)\right\} \\
&\quad = -\sum_{\mathbf{n}}\sum_{\mathbf{e}} \mu_+(1, N_s, e_s) n_s^1 p(\mathbf{n}, \mathbf{e}, t_h) \\
&\quad = -\langle \mu_+(1, N_s, e_s) n_s^1 \rangle.
\end{aligned}$$

The contributions from the terms describing movement in physical space are the same as in Eq. (S3) with $j = 1$ and the growth terms are also the same as those in Eq. (S3) with $i = s$ and

$j = 1$. Putting these together, we get

$$\begin{aligned}\frac{\partial}{\partial t}\langle n_s^1\rangle &= \frac{1}{\Delta_t}\langle \mu_-(2, N_s, e_s)n_s^2\rangle - \frac{1}{\Delta_t}\langle \mu_+(1, N_s, e_s)n_s^1\rangle \\ &+ \frac{1}{\Delta_t}\langle \beta_+(1, N_s, e_s)n_{s-1}^1\rangle + \frac{1}{\Delta_t}\langle \beta_-(1, N_s, e_s)n_{s+1}^1\rangle \\ &- \frac{1}{\Delta_t}\langle \beta_-(1, N_{s-1}, e_{s-1})n_s^1\rangle - \frac{1}{\Delta_t}\langle \beta_+(1, N_{s+1}, e_{s+1})n_s^1\rangle \\ &+ \frac{1}{\Delta_t}\langle \gamma(1, N_s, e_s)n_s^1\rangle. \end{aligned} \tag{S14}$$

Then, in the limit $\Delta_x, \Delta_y, \Delta_t \to 0$, the continuum equivalents of the dependent variables, Eq. (S14) can be rewritten at $y = Y_{\min}$ as follows:

$$\begin{aligned}\frac{\partial}{\partial t}n(x, y, t) &= \frac{1}{\Delta_t}\beta_+(y, \rho(x, t), e(x, t))n(x - \Delta_x, y, t) \\ &+ \frac{1}{\Delta_t}\beta_-(y, \rho(x, t), e(x, t))n(x + \Delta_x, y, t) \\ &- \frac{1}{\Delta_t}\beta_-(y, \rho(x - \Delta_x, t), e(x - \Delta_x, t))n(x, y, t) \\ &- \frac{1}{\Delta_t}\beta_+(y, \rho(x + \Delta_x, t), e(x + \Delta_x, t))n(x, y, t) \\ &+ \frac{1}{\Delta_t}\mu_-(y + \Delta_y, \rho(x, t), e(x, t))n(x, y + \Delta_y, t) \\ &- \frac{1}{\Delta_t}\mu_+(y, \rho(x, t), e(x, t))n(x, y, t) \\ &+ \frac{1}{\Delta_t}\gamma(y, \rho(x, t), e(x, t))n(x, y, t). \end{aligned}$$

Then, employing the aforementioned Taylor expansions, we find

$$
\begin{aligned}
\frac{\partial}{\partial t} n(x,y,t) = {} & \frac{1}{\Delta_t}\beta_+(y,\rho(x,t),e(x,t)) \times \\
& \qquad \left[n(x,y,t) - \Delta_x \frac{\partial}{\partial x} n(x,y,t) + \frac{\Delta_x^2}{2}\frac{\partial^2}{\partial x^2} n(x,y,t) \right] \\
& + \frac{1}{\Delta_t}\beta_-(y,\rho(x,t),e(x,t)) \times \\
& \qquad \left[n(x,y,t) + \Delta_x \frac{\partial}{\partial x} n(x,y,t) + \frac{\Delta_x^2}{2}\frac{\partial^2}{\partial x^2} n(x,y,t) \right] \\
& - \frac{1}{\Delta_t} n(x,y,t) \times \\
& \qquad \left[\beta_-(y,\rho(x,t),e(x,t)) - \Delta_x \frac{\partial}{\partial x}\beta_-(y,\rho(x,t),e(x,t) \right. \\
& \qquad \qquad \left. + \frac{\Delta_x^2}{2}\frac{\partial^2}{\partial x^2}\beta_-(y,\rho(x,t),e(x,t) \right] \\
& - \frac{1}{\Delta_t} n(x,y,t) \times \\
& \qquad \left[\beta_+(y,\rho(x,t),e(x,t)) + \Delta_x \frac{\partial}{\partial x}\beta_+(y,\rho(x,t),e(x,t) \right. \\
& \qquad \qquad \left. + \frac{\Delta_x^2}{2}\frac{\partial^2}{\partial x^2}\beta_+(y,\rho(x,t),e(x,t) \right] \\
& + \frac{1}{\Delta_t} \left[n(x,y,t) + \Delta_y \frac{\partial}{\partial y} n(x,y,t) + \frac{\Delta_y^2}{2}\frac{\partial^2}{\partial y^2} n(x,y,t) \right] \times \\
& \qquad \left[\mu_-(y,\rho(x,t),e(x,t)) + \Delta_y \frac{\partial}{\partial y}\mu_-(y,\rho(x,t),e(x,t) \right. \\
& \qquad \qquad \left. + \frac{\Delta_y^2}{2}\frac{\partial^2}{\partial y^2}\mu_-(y,\rho(x,t),e(x,t) \right] \\
& - \frac{1}{\Delta_t}\mu_+(y,\rho(x,t),e(x,t)) n(x,y,t) \\
& + \frac{1}{\Delta_t}\gamma(y,\rho(x,t),e(x,t)) n(x,y,t).
\end{aligned}
$$

This can be rewritten as (dropping the dependent variables for simplicity)

$$
\begin{aligned}
\frac{\partial n}{\partial t} = {} & \frac{1}{\Delta_t}\left(\mu_- - \mu_+\right) n + \frac{\Delta_y}{\Delta_t}\frac{\partial}{\partial y}\left(\mu_- n\right) + \frac{\Delta_y^2}{2\Delta_t}\frac{\partial^2}{\partial y^2}\left(\mu_- n\right) + \frac{\Delta_x}{\Delta_t}\frac{\partial}{\partial x}\Big(\left(\beta_- - \beta_+\right) n\Big) \\
& + \frac{\Delta_x^2}{2\Delta_t}\frac{\partial}{\partial x}\left((\beta_- + \beta_+)\frac{\partial n}{\partial x} - n\frac{\partial}{\partial x}(\beta_- + \beta_+)\right) + \frac{1}{\Delta_t}\gamma n,
\end{aligned}
\tag{S15}
$$

at $y = Y_{\min}$. Recalling that

$$\begin{aligned}
\lim_{\Delta_x,\Delta_t\to 0} \frac{\Delta_x}{\Delta_t}\Big(\beta_-(y,\rho(x,t),e(x,t)) - \beta_+(y,\rho(x,t),e(x,t))\Big) &= v^m(y,\rho(x,t),e(x,t)),\\
\lim_{\Delta_x,\Delta_t\to 0} \frac{\Delta_x^2}{2\Delta_t}\Big(\beta_-(y,\rho(x,t),e(x,t)) + \beta_+(y,\rho(x,t),e(x,t))\Big) &= D^m(y,\rho(x,t),e(x,t)),\\
\lim_{\Delta_y,\Delta_t\to 0} \frac{\Delta_y}{\Delta_t}\Big(\mu_-\left(y,\rho(x,t),e(x,t)\right) - \mu_+\left(y,\rho(x,t),e(x,t)\right)\Big) &= v^p(y,\rho(x,t),e(x,t)),\\
\lim_{\Delta_y,\Delta_t\to 0} \frac{\Delta_y^2}{2\Delta_t}\Big(\mu_-\left(y,\rho(x,t),e(x,t)\right) + \mu_+\left(y,\rho(x,t),e(x,t)\right)\Big) &= D^p(y,\rho(x,t),e(x,t)),\\
\lim_{\Delta_t\to 0} \frac{1}{\Delta_t}\gamma\left(y,\rho(x,t),e(x,t)\right) &= r(y,\rho(x,t),e(x,t)),
\end{aligned}$$

such that

$$\mu_\pm = \frac{D^p\Delta_t}{\Delta_y^2} \mp \frac{v^p\Delta_t}{2\Delta_y},$$

then Eq. (S15) can be rewritten as

$$\begin{aligned}
\frac{\partial n}{\partial t} = \frac{1}{\Delta_y}v^p n &+ \frac{\partial}{\partial y}\left(\frac{1}{2}v^p n + \frac{1}{\Delta_y}D^p n\right) + \frac{1}{2}\frac{\partial^2}{\partial y^2}\left(D^p n\right) + \frac{\partial}{\partial x}(v^m n)\\
&+ \frac{\partial}{\partial x}\left(D^m\frac{\partial n}{\partial x} - n\frac{\partial}{\partial x}D^m\right) + rn.
\end{aligned}$$

Therefore, in order to prevent blow-up of terms at $y = Y_{\min}$, we require that

$$v^p n + \frac{\partial}{\partial y}(D^p n) = 0 \qquad \text{at} \quad y = Y_{\min}.$$

Boundary condition at $y = Y_{\max}$. Returning to the Eq. (S1), we seek an equation for the evolution of the cell number on the upper most lattice site in phenotype space, corresponding

to the site y_{N_y+1}. To find this, we multiply Eq. (S1) by $n_s^{N_y+1}$ and sum over possible states $\mathbf{n}$:

$$\begin{aligned}
&\Delta_t \sum_{\mathbf{n}} \sum_{\mathbf{e}} n_s^{N_y+1} \frac{\partial}{\partial t} p(\mathbf{n}, \mathbf{e}, t_h) = \\
&\quad \sum_{\mathbf{n}} \sum_{\mathbf{e}} n_s^{N_y+1} \sum_{i=1}^{N_x+1} \sum_{j=1}^{N_y} \mu_-(j+1, N_i, e_i) \left\{ (n_i^{j+1}+1) p(U^{\mathrm{p}}_{i,j}\mathbf{n}, \mathbf{e}, t_h) - n_i^{j+1} p(\mathbf{n}, \mathbf{e}, t_h) \right\} \\
&\quad + \sum_{\mathbf{n}} \sum_{\mathbf{e}} n_s^{N_y+1} \sum_{i=1}^{N_x+1} \sum_{j=2}^{N_y+1} \mu_+(j-1, N_i, e_i) \left\{ (n_i^{j-1}+1) p(D^{\mathrm{p}}_{i,j}\mathbf{n}, \mathbf{e}, t_h) - n_i^{j-1} p(\mathbf{n}, \mathbf{e}, t_h) \right\} \\
&\quad + \sum_{\mathbf{n}} \sum_{\mathbf{e}} n_s^{N_y+1} \sum_{i=1}^{N_x} \sum_{j=1}^{N_y+1} \beta_-(j, N_i, e_i) \left\{ (n_{i+1}^{j}+1) p(R^{\mathrm{m}}_{i,j}\mathbf{n}, \mathbf{e}, t_h) - n_{i+1}^{j} p(\mathbf{n}, \mathbf{e}, t_h) \right\} \\
&\quad + \sum_{\mathbf{n}} \sum_{\mathbf{e}} n_s^{N_y+1} \sum_{i=2}^{N_x+1} \sum_{j=1}^{N_y+1} \beta_+(j, N_i, e_i) \left\{ (n_{i-1}^{j}+1) p(L^{\mathrm{m}}_{i,j}\mathbf{n}, \mathbf{e}, t_h) - n_{i-1}^{j} p(\mathbf{n}, \mathbf{e}, t_h) \right\} \\
&\quad + \sum_{\mathbf{n}} \sum_{\mathbf{e}} n_s^{N_y+1} \sum_{i=1}^{N_x+1} \sum_{j=1}^{N_y+1} \gamma(j, N_i, e_i) \left\{ \gamma(j, N_i-1, e_i)(n_i^{j}-1) p(G_{i,j}\mathbf{n}, \mathbf{e}, t_h) - \gamma(j, N_i, e_i) n_i^{j} p(\mathbf{n}, \mathbf{e}, t_h) \right\}.
\end{aligned} \tag{S16}$$

Using the same methods as on the boundaries in physical space, we can change variables in each term to find an equation for evolution of cell number. Consider first the first term on the right-hand side. From previous analysis, we know that the only non-zero contributions come from when $j = N_y$ and $i = s$. In the second term, the non-zero terms are $j = N_y + 1$ and $i = s$. The third term gives contributions when $j = N_y + 1$ and $i = s$ or $i = s - 1$. In the fourth term, there are non-zero contributions when $j = N_y + 1$ and $i = s$ or $i = s + 1$. The final term produces non-zero contributions only when $i = s$ and $j = N_y + 1$. Thus, Eq. (S16) can be written as

$$\begin{aligned}
&\Delta_t \sum_{\mathbf{n}} \sum_{\mathbf{e}} n_s^{N_y+1} \frac{\partial}{\partial t} p(\mathbf{n}, \mathbf{e}, t_h) = \\
&\quad \sum_{\mathbf{n}} \sum_{\mathbf{e}} n_s^{N_y+1} \mu_-(N_y+1, N_s, e_s) \left\{ (n_s^{N_y+1}+1) p(U^{\mathrm{p}}_{s,N_y}\mathbf{n}, \mathbf{e}, t_h) - n_s^{N_y+1} p(\mathbf{n}, \mathbf{e}, t_h) \right\} \\
&\quad + \sum_{\mathbf{n}} \sum_{\mathbf{e}} n_s^{N_y+1} \mu_+(N_y, N_s, e_s) \left\{ (n_s^{N_y}+1) p(D^{\mathrm{p}}_{s,N_y+1}\mathbf{n}, \mathbf{e}, t_h) - n_s^{N_y} p(\mathbf{n}, \mathbf{e}, t_h) \right\} \\
&\quad + \sum_{\mathbf{n}} \sum_{\mathbf{e}} n_s^{N_y+1} \sum_{i=s,s-1} \beta_-(N_y+1, N_i, e_i) \left\{ (n_{i+1}^{N_y+1}+1) p(R^{\mathrm{m}}_{i,N_y+1}\mathbf{n}, \mathbf{e}, t_h) - n_{i+1}^{N_y+1} p(\mathbf{n}, \mathbf{e}, t_h) \right\} \\
&\quad + \sum_{\mathbf{n}} \sum_{\mathbf{e}} n_s^{N_y+1} \sum_{i=s,s+1} \beta_+(N_y+1, N_i, e_i) \left\{ (n_{i-1}^{N_y+1}+1) p(L^{\mathrm{m}}_{i,N_y+1}\mathbf{n}, \mathbf{e}, t_h) - n_{i-1}^{N_y+1} p(\mathbf{n}, \mathbf{e}, t_h) \right\} \\
&\quad + \sum_{\mathbf{n}} \sum_{\mathbf{e}} n_s^{N_y+1} \left\{ \gamma(N_y+1, N_s-1, e_s)(n_s^{N_y+1}-1) p(G_{s,N_y+1}\mathbf{n}, \mathbf{e}, t_h) - \gamma(N_y+1, N_s, e_s) n_s^{N_y+1} p(\mathbf{n}, \mathbf{e}, t_h) \right\}.
\end{aligned} \tag{S17}$$

The first term in Eq. (S17) can be rewritten using the change of variables ($\bar{n}_s^{N_y+1} = n_s^{N_y+1} + 1$ and $\bar{n}_s^{N_y} = n_s^{N_y} - 1$) in the following way:

$$\sum_{\mathbf{n}} \sum_{\mathbf{e}} n_s^{N_y+1} \mu_-(N_y + 1, N_s, e_s) \left\{ (n_s^{N_y+1} + 1) p(U^{\mathrm{p}}_{s,N_y} \mathbf{n}, \mathbf{e}, t_h) - n_s^{N_y+1} p(\mathbf{n}, \mathbf{e}, t_h) \right\}$$
$$= \sum_{\mathbf{n}} \sum_{\mathbf{e}} n_s^{N_y+1} \mu_-(N_y + 1, N_s, e_s) \times$$
$$\left\{ (n_s^{N_y+1} + 1) p([n_s^1, \ldots, n_s^{N_y} - 1, n_s^{N_y+1} - 1], \mathbf{e}, t_h) - n_s^{N_y+1} p(\mathbf{n}, \mathbf{e}, t_h) \right\}$$
$$= \sum_{\mathbf{n}} \sum_{\mathbf{e}} \mu_-(N_y + 1, N_s, e_s) \times$$
$$\left\{ \bar{n}_s^{N_y+1} (\bar{n}_s^{N_y+1} - 1) p([n_s^1, \ldots, \bar{n}_s^{N_y}, \bar{n}_s^{N_y+1}], \mathbf{e}, t_h) - n_s^{N_y+1} n_s^{N_y+1} p(\mathbf{n}, \mathbf{e}, t_h) \right\}$$
$$= \sum_{\mathbf{n}} \sum_{\mathbf{e}} \mu_-(N_y + 1, N_s, e_s) \left\{ n_s^{N_y+1} (n_s^{N_y+1} - 1) p(\mathbf{n}, \mathbf{e}, t_h) - n_s^{N_y+1} n_s^{N_y+1} p(\mathbf{n}, \mathbf{e}, t_h) \right\}$$
$$= -\sum_{\mathbf{n}} \sum_{\mathbf{e}} \mu_-(N_y + 1, N_s, e_s) n_s^{N_y+1} p(\mathbf{n}, \mathbf{e}, t_h)$$
$$= -\langle \mu_-(N_y + 1, N_s, e_s) n_s^{N_y+1} \rangle.$$

The second term in Eq. (S17) can then be rewritten as the following (using $\bar{n}_s^{N_y+1} = n_s^{N_y+1} - 1$ and $\bar{n}_s^{N_y} = n_s^{N_y} + 1$):

$$\sum_{\mathbf{n}} \sum_{\mathbf{e}} n_s^{N_y+1} \mu_+(N_y, N_s, e_s) \left\{ (n_s^{N_y} + 1) p(D^{\mathrm{p}}_{s,N_y+1} \mathbf{n}, \mathbf{e}, t_h) - n_s^{N_y} p(\mathbf{n}, \mathbf{e}, t_h) \right\}$$
$$= \sum_{\mathbf{n}} \sum_{\mathbf{e}} n_s^{N_y+1} \mu_+(N_y, N_s, e_s) \times$$
$$\left\{ (n_s^{N_y} + 1) p([n_s^1, \ldots, n_s^{N_y} + 1, n_s^{N_y+1} - 1], \mathbf{e}, t_h) - n_s^{N_y} p(\mathbf{n}, \mathbf{e}, t_h) \right\}$$
$$= \sum_{\mathbf{n}} \sum_{\mathbf{e}} \mu_+(N_y, N_s, e_s) \times$$
$$\left\{ \bar{n}_s^{N_y} (\bar{n}_s^{N_y+1} + 1) p([n_s^1, \ldots, \bar{n}_s^{N_y}, \bar{n}_s^{N_y+1}], \mathbf{e}, t_h) - n_s^{N_y} n_s^{N_y+1} p(\mathbf{n}, \mathbf{e}, t_h) \right\}$$
$$= \sum_{\mathbf{n}} \sum_{\mathbf{e}} \mu_+(N_y, N_s, e_s) \times$$
$$\left\{ n_s^{N_y} (n_s^{N_y+1} + 1) p(\mathbf{n}, \mathbf{e}, t_h) - n_s^{N_y} n_s^{N_y+1} p(\mathbf{n}, \mathbf{e}, t_h) \right\}$$
$$= \sum_{\mathbf{n}} \sum_{\mathbf{e}} \mu_+(N_y, N_s, e_s) n_s^{N_y} p(\mathbf{n}, \mathbf{e}, t_h)$$
$$= \langle \mu_+(N_y, N_s, e_s) n_s^{N_y} \rangle.$$

The contributions from the terms describing movement in physical space are the same as in the main body Eq. (S3) where $j = N_y$. This is also true for the growth terms with $i = s$ and $j = N_y$.

Putting these together, we get

$$\begin{aligned}\frac{\partial}{\partial t}\langle n_s^{N_y+1}\rangle &= \frac{1}{\Delta_t}\langle \mu_+(N_y, N_s, e_s)n_s^{N_y}\rangle - \frac{1}{\Delta_t}\langle \mu_-(N_y+1, N_s, e_s)n_s^{N_y+1}\rangle \\ &+ \frac{1}{\Delta_t}\langle \beta_+(N_y+1, N_s, e_s)n_{s-1}^{N_y+1}\rangle + \frac{1}{\Delta_t}\langle \beta_-(N_y+1, N_s, e_s)n_{s+1}^{N_y+1}\rangle \\ &- \frac{1}{\Delta_t}\langle \beta_-(N_y+1, N_{s-1}, e_{s-1})n_s^{N_y+1}\rangle - \frac{1}{\Delta_t}\langle \beta_+(N_y+1, N_{s+1}, e_{s+1})n_s^{N_y+1}\rangle \\ &+ \frac{1}{\Delta_t}\langle \gamma(N_y+1, N_s, e_s)n_s^{N_y+1}\rangle. \end{aligned} \tag{S18}$$

We can take the limit $\Delta_x, \Delta_y, \Delta_t \to 0$, such that Eq. (S18) can be rewritten as the following at $y = Y_{\max}$:

$$\begin{aligned}\frac{\partial}{\partial t}n(x,y,t) &= \frac{1}{\Delta_t}\beta_+(y, \rho(x,t), e(x,t))n(x-\Delta_x, y, t) \\ &+ \frac{1}{\Delta_t}\beta_-(y, \rho(x,t), e(x,t))n(x+\Delta_x, y, t) \\ &- \frac{1}{\Delta_t}\beta_-(y, \rho(x-\Delta_x,t), e(x-\Delta_x,t))n(x,y,t) \\ &- \frac{1}{\Delta_t}\beta_+(y, \rho(x+\Delta_x,t), e(x+\Delta_x,t))n(x,y,t) \\ &+ \frac{1}{\Delta_t}\mu_+(y-\Delta_y, \rho(x,t), e(x,t))n(x, y-\Delta_y, t) \\ &- \frac{1}{\Delta_t}\mu_-(y, \rho(x,t), e(x,t))n(x,y,t) \\ &+ \frac{1}{\Delta_t}\gamma(y, \rho(x,t), e(x,t))n(x,y,t). \end{aligned}$$

Using Taylor series expansions, we find

$$
\begin{aligned}
\frac{\partial}{\partial t}n(x,y,t) = &\frac{1}{\Delta_t}\beta_+(y,\rho(x,t),e(x,t))\times \\
&\qquad\left[n(x,y,t) - \Delta_x\frac{\partial}{\partial x}n(x,y,t) + \frac{\Delta_x^2}{2}\frac{\partial^2}{\partial x^2}n(x,y,t)\right] \\
&+\frac{1}{\Delta_t}\beta_-(y,\rho(x,t),e(x,t))\times \\
&\qquad\left[n(x,y,t) + \Delta_x\frac{\partial}{\partial x}n(x,y,t) + \frac{\Delta_x^2}{2}\frac{\partial^2}{\partial x^2}n(x,y,t)\right] \\
&-\frac{1}{\Delta_t}n(x,y,t)\times \\
&\qquad\left[\beta_-(y,\rho(x,t),e(x,t)) - \Delta_x\frac{\partial}{\partial x}\beta_-(y,\rho(x,t),e(x,t)\right. \\
&\qquad\qquad\left.+\frac{\Delta_x^2}{2}\frac{\partial^2}{\partial x^2}\beta_-(y,\rho(x,t),e(x,t)\right] \\
&-\frac{1}{\Delta_t}n(x,y,t)\times \\
&\qquad\left[\beta_+(y,\rho(x,t),e(x,t)) + \Delta_x\frac{\partial}{\partial x}\beta_+(y,\rho(x,t),e(x,t)\right. \\
&\qquad\qquad\left.+\frac{\Delta_x^2}{2}\frac{\partial^2}{\partial x^2}\beta_+(y,\rho(x,t),e(x,t)\right] \\
&+\frac{1}{\Delta_t}\left[n(x,y,t) - \Delta_y\frac{\partial}{\partial y}n(x,y,t) + \frac{\Delta_y^2}{2}\frac{\partial^2}{\partial y^2}n(x,y,t)\right]\times \\
&\qquad\left[\mu_+(y,\rho(x,t),e(x,t)) - \Delta_y\frac{\partial}{\partial y}\mu_+(y,\rho(x,t),e(x,t)\right. \\
&\qquad\qquad\left.+\frac{\Delta_y^2}{2}\frac{\partial^2}{\partial y^2}\mu_+(y,\rho(x,t),e(x,t)\right] \\
&-\frac{1}{\Delta_t}\mu_-(y,\rho(x,t),e(x,t))n(x,y,t) \\
&+\frac{1}{\Delta_t}\gamma(y,\rho(x,t),e(x,t))n(x,y,t).
\end{aligned}
$$

Now, dropping the dependent variables for simplicity, we find

$$
\begin{aligned}
\frac{\partial n}{\partial t} = &-\frac{1}{\Delta_t}(\mu_- - \mu_+)n - \frac{\Delta_y}{\Delta_t}\frac{\partial}{\partial y}(\mu_+ n) + \frac{\Delta_y^2}{2\Delta_t}\frac{\partial^2}{\partial y^2}(\mu_+ n) + \frac{\Delta_x}{\Delta_t}\frac{\partial}{\partial x}\Big((\beta_- - \beta_+)\,n\Big) \\
&+\frac{\Delta_x^2}{2\Delta_t}\frac{\partial}{\partial x}\left((\beta_- + \beta_+)\frac{\partial n}{\partial x} - n\frac{\partial}{\partial x}(\beta_- + \beta_+)\right) + \frac{1}{\Delta_t}\gamma n.
\end{aligned}
$$

at $y = Y_{\max}$. Recalling that

$$\begin{aligned}
\lim_{\Delta_x,\Delta_t\to 0} \frac{\Delta_x}{\Delta_t}\Big(\beta_-(y,\rho(x,t),e(x,t)) - \beta_+(y,\rho(x,t),e(x,t))\Big) &= v^m(y,\rho(x,t),e(x,t)),\\
\lim_{\Delta_x,\Delta_t\to 0} \frac{\Delta_x^2}{2\Delta_t}\Big(\beta_-(y,\rho(x,t),e(x,t)) + \beta_+(y,\rho(x,t),e(x,t))\Big) &= D^m(y,\rho(x,t),e(x,t)),\\
\lim_{\Delta_y,\Delta_t\to 0} \frac{\Delta_y}{\Delta_t}\Big(\mu_-\left(y,\rho(x,t),e(x,t)\right) - \mu_+\left(y,\rho(x,t),e(x,t)\right)\Big) &= v^p(y,\rho(x,t),e(x,t)),\\
\lim_{\Delta_y,\Delta_t\to 0} \frac{\Delta_y^2}{2\Delta_t}\Big(\mu_-\left(y,\rho(x,t),e(x,t)\right) + \mu_+\left(y,\rho(x,t),e(x,t)\right)\Big) &= D^p(y,\rho(x,t),e(x,t)),\\
\lim_{\Delta_t\to 0} \frac{1}{\Delta_t}\gamma\left(y,\rho(x,t),e(x,t)\right) &= r(y,\rho(x,t),e(x,t)),
\end{aligned}$$

such that

$$\mu_\pm = \frac{D^p\Delta_t}{\Delta_y^2} \mp \frac{v^p\Delta_t}{2\Delta_y},$$

then the equation can be rewritten as

$$\begin{aligned}
\frac{\partial n}{\partial t} = &-\frac{1}{\Delta_y}v^p n + \frac{\partial}{\partial y}\left(\frac{1}{2}v^p n - \frac{1}{\Delta_y}D^p n\right) + \frac{1}{2}\frac{\partial^2}{\partial y^2}\left(D^p n\right) + \frac{\partial}{\partial x}(v^m n)\\
&+ \frac{\partial}{\partial x}\left(D^m\frac{\partial n}{\partial x} - n\frac{\partial}{\partial x}D^m\right) + rn.
\end{aligned}$$

Therefore, in order to prevent blow-up of terms at $y = Y_{\max}$, we require that

$$-v^p n - \frac{\partial}{\partial y}(D^p n) = 0 \qquad\qquad \text{at} \quad y = Y_{\max}.$$

S1.2 Equation for the density of the local environment

Following the assumptions outlined in Sec. 2 and methodology above, we can review the master equation (S1) describing the evolution of the number of elements of the local environment at position x_i, denoted e_i, and multiply by e_s and sum over all possible states $\mathbf{e}$ and $\mathbf{n}$ to get

$$\begin{aligned}
&\sum_{\mathbf{e}}\sum_{\mathbf{n}} e_s\Delta_t\frac{\partial}{\partial t}p(\mathbf{n},\mathbf{e},t_h)\\
&= \sum_{\mathbf{e}}\sum_{\mathbf{n}} e_s\sum_{i=1}^{N_x+1}\sum_{j=1}^{N_y+1}\lambda(j,n_i^j)\left\{(e_i+1)p(\mathbf{n},H_i\mathbf{e},t_h) - e_i p(\mathbf{n},\mathbf{e},t_h)\right\}\\
&= \sum_{\mathbf{e}}\sum_{\mathbf{n}} e_s\sum_{i=1}^{N_x+1}\sum_{j=1}^{N_y+1}\lambda(j,n_i^j)\left\{(e_i+1)p(\mathbf{n},[e_1,\ldots,e_i+1,\ldots,e_{N_x+1}],t_h) - e_i p(\mathbf{n},\mathbf{e},t_h)\right\},
\end{aligned}$$

recalling that contributions from the terms describing cell dynamics only sum to zero. Now we consider two cases: $i = s$ and $i \neq s$. First, consider $i \neq s$ and use the change of variables $\bar{e}_i = e_i + 1$ in the second term, and then drop the bar:

$$\begin{aligned}
&\sum_{\mathbf{e}}\sum_{\mathbf{n}} e_s \sum_{\substack{i=1,\\ i\neq s}}^{N_x+1} \sum_{j=1}^{N_y+1} \lambda(j, n_i^j) \left\{(e_i+1)p(\mathbf{n}, [e_1, \ldots, e_i+1, \ldots, e_{N_x+1}], t_h) - e_i p(\mathbf{n}, \mathbf{e}, t_h)\right\} \\
&= \sum_{\mathbf{e}}\sum_{\mathbf{n}} e_s \sum_{\substack{i=1,\\ i\neq s}}^{N_x+1} \sum_{j=1}^{N_y+1} \lambda(j, n_i^j) \left\{\bar{e}_i p(\mathbf{n}, [e_1, \ldots, \bar{e}_i, \ldots, e_{N_x+1}], t_h) - e_i p(\mathbf{n}, \mathbf{e}, t_h)\right\} \\
&= \sum_{\mathbf{e}}\sum_{\mathbf{n}} e_s \sum_{\substack{i=1,\\ i\neq s}}^{N_x+1} \sum_{j=1}^{N_y+1} \lambda(j, n_i^j) \left\{e_i p(\mathbf{n}, \mathbf{e}, t_h) - e_i p(\mathbf{n}, \mathbf{e}, t_h)\right\} \\
&= 0.
\end{aligned}$$

Now consider the case when $i = s$ and use the change of variables $\bar{e}_s = e_s + 1$:

$$\begin{aligned}
&\sum_{\mathbf{e}}\sum_{\mathbf{n}} \sum_{j=1}^{N_y+1} e_s \lambda(j, n_s^j) \left\{(e_s+1)p(\mathbf{n}, [e_1, \ldots, e_s+1, \ldots, e_{N_x+1}], t_h) - e_s p(\mathbf{n}, \mathbf{e}, t_h)\right\} \\
&= \sum_{\mathbf{e}}\sum_{\mathbf{n}} \sum_{j=1}^{N_y+1} \lambda(j, n_s^j) \left\{\bar{e}_s(\bar{e}_s - 1)p(\mathbf{n}, [e_1, \ldots, \bar{e}_s, \ldots, e_{N_x+1}], \mathbf{e}, t_h) - e_s^2 p(\mathbf{n}, \mathbf{e}, t_h)\right\} \\
&= \sum_{\mathbf{e}}\sum_{\mathbf{n}} \sum_{j=1}^{N_y+1} e_s \lambda(j, n_s^j) \left\{(e_s - 1)p(\mathbf{n}, \mathbf{e}, t_h) - e_s p(\mathbf{n}, \mathbf{e}, t_h)\right\} \\
&= -\sum_{\mathbf{e}}\sum_{\mathbf{n}} \sum_{j=1}^{N_y+1} \lambda(j, n_s^j) e_s p(\mathbf{n}, \mathbf{e}, t_h) \\
&= -\sum_{j=1}^{N_y+1} \langle \lambda(j, n_s^j) e_s \rangle.
\end{aligned}$$

Putting this together, we get

$$\Delta_t \frac{\partial}{\partial t}\langle e_s \rangle = -\sum_{j=1}^{N_y+1} \langle \lambda(j, n_s^j) e_s \rangle.$$

Defining

$$\lim_{\Delta_t \to 0} \frac{1}{\Delta_t} \lambda(y, n(x, y, t)) = \nu(y, n(x, y, t)),$$

which we can substitute into the equation, rearrange and take limits as $\Delta_x, \Delta_y, \Delta_t \to 0$, to find that the differential equation for the density of the local environment, $e(x,t)$, is given by

$$\frac{\partial}{\partial t} e(x,t) = -\int_{y=Y_{\min}}^{y=Y_{\max}} \nu(y, n(x,y,t)) e(x,t) \mathrm{d}y.$$

No boundary conditions are required for this equation.

S2 Individual-based model functions

S2.1 Phenotypic structuring during range expansion

As per the rules described in Sec. 2, we can write functions to describe the movement and growth of cells over time. In particular, noting that in this case we have homogeneous cells, with constant random movement in all directions, then we can write

$$\beta_{\pm}(j, N_{i\pm1}, e_{i\pm1}) = 1.$$

Furthermore, when considering KPP type invasion, we know that cells grow faster in areas with higher amounts of available space, which can be modelled as

$$\gamma_K(j, N_i) = 1 - \frac{N_i}{\kappa},$$

whereas, with the addition of the Allee effect, we instead have

$$\gamma_A(j, N_i) = \left(1 - \frac{N_i}{\kappa}\right)(N_i - p^*),$$

with $p^* \in (0, 1/2)$ and $\kappa > 0$ describing the maximum total number of cells that can fit in any single site. Implementing these functional forms during the coarse-graining process described in Sec. S1, absorbing constants in the continuum limit and rescaling as appropriate, the resulting continuum equation is given by Eq. (14) with functions (15) and (16).

S2.2 A go-or-grow model of cells invading the extracellular matrix (ECM)

When describing cells moving into the extracellular matrix (ECM), we know that volume filling constraints will affect the movement in physical space. In fact, as space decreases, cells have less space in which to move. Furthermore, we implement a continuum of cell phenotypes in this case, such that cells in phenotypic state $j = Y_{\max}$ are the most proliferative, but least motile and degrading cells. As such, the individual-based functions describing movement in physical space can be written as

$$\beta_{\pm}(j, N_{i\pm1}, e_{i\pm1}) = (1 - j)\left(1 - \frac{N_{i\pm1} + e_{i\pm1}}{\kappa}\right),$$

where $\kappa > 0$ is the total number of available sites for cells and ECM elements, known as the carrying capacity.

Cells are able to proliferate more rapidly when there is a larger amount of available space,

and when they occupy a phenotypic state with higher values. As such, we have that

$$\gamma(j, N_i, e_i) = j\left(1 - \frac{N_i + e_i}{\kappa}\right).$$

Alternatively, it is cells in a lower phenotypic state, j, that degrade the surrounding ECM at a higher rate. The corresponding function to describe this is given by

$$\lambda(j, n_i^j) = (1 - j)n_i^j.$$

In Sec. 3.3, we consider a number of different functions to describe movement in phenotypic space. The first phenotypic drift term we consider is cell-dependent drift, where cells transition into a phenotypic state with lower values at an increasing rate in regions with more cells present. The second phenotypic drift term we evaluate considers the role of the ECM in determining phenotypic transitions. In this case, cells transition to phenotypic states with lower values as the number of ECM elements in the same physical site increases. Finally we consider space-dependent phenotype transitions such that cells move into phenotypic sites with higher values at an increasing rate as the available space in the same physical site increases. These options are all described in Table 1.

Phenotypic drift	$\mu_-(j, N_i, e_i)$	$\mu_+(j, N_i, e_i)$
Cell-dependent	$\frac{N_i}{\kappa}$	$1 - \frac{N_i}{\kappa}$
ECM-dependent	$\frac{e_i}{\kappa}$	$1 - \frac{e_i}{\kappa}$
Space-dependent	$\frac{N_i + e_i}{\kappa}$	$1 - \frac{N_i + e_i}{\kappa}$

Table 1: Table listing the individual-based functions used during coarse-graining, that correspond to those continuum equivalents described in Table 1. The functions shown describe the probabilities of transitions up and down the phenotype space, $\mu_+(j, N_i, e_i)$ and $\mu_-(j, N_i, e_i)$, respectively.

Implementing the individual-based functions described above during the coarse-graining process, absorbing parameters and rescaling gives the resulting continuum equations and functions as stated in Sec. 3.3 of the main text.

S2.3 T cell exhaustion

In the case where we consider T cell exhaustion, we are simulating both T cells and tumour cells. Both the T cells and tumour cells can move and grow, whilst T cells have a further variable, exhaustion, attached to them, and they are able to kill off the tumour cells.

The movement of both the tumour cells and T cells is subject to volume exclusion, but also depends on the exhaustion level of the cells for the T cells. As such, we can write that the individual-based functions describing the movement of the T cells is given by

$$\beta_{\pm}(j, N_{i\pm1}, e_{i\pm1}) = j\left(1 - \frac{N_{i\pm1} + e_{i\pm1}}{\kappa}\right),$$

where $\kappa > 0$ is the carrying capacity of each physical site, i. Alternatively, the movement of the tumour cells does not depend on the phenotype of the cells. For some rate $\bar{r_{\pm}} \geq 0$, describing the movement of the tumour cells, we can write

$$r_{\pm}(N_{i\pm1}, e_{i\pm1}) = \bar{r_{\pm}}\left(1 - \frac{N_{i\pm1} + e_{i\pm1}}{\kappa}\right),$$

which coarse-grains to become the function given by $D^C(\rho(x,t), C(x,t))$ in the main text, using the same method as for $D^m(y, \rho(x,t), C(x,t))$.

T cells are able to divide and produce a daughter cell in the same phenotypic and physical site at a rate described by $\gamma_1 \geq 0$. However the rate of reproduction depends on available space and is also greater for less exhausted cells, in a phenotypic state with higher values. T cells can also die at a rate $\gamma_0 \geq 0$ which increases as they exhaust. As such, the function describing the net growth of the T cells at an individual-level is given by

$$\gamma(j, N_i, e_i) = \gamma_1 j\left(1 - \frac{N_i + e_i}{\kappa}\right) - \gamma_0(1 - j).$$

Concurrently, the probability of tumour cell growth increases in more available space. This probability behaves in a similar manner to $\gamma(j, N_i)$ in the previous application, but without phenotype dependence. As such, we write

$$b(N_i, e_i) = 1 - \frac{N_i + e_i}{\kappa}.$$

This term coarse-grains to become the function given by $g(\rho(x,t), C(x,t))$ in the main text, using the same method as for $r(y, \rho(x,t), C(x,t))$.

T cells exhaust as a result of interactions with (being in the same site as) tumour cells. They also naturally exhaust. Both of these occur faster when a cell is less exhausted (in a higher

phenotypic state). To implement this, we write that the probability of cells moving up or down in phenotype space can be written as

$$\mu_{\pm}(j, e_i) = \frac{1}{2}\left(1 \pm jk_1 \pm jk_2 e_i\right),$$

where $k_1, k_2 \geq 0$ describe the exhaustion rate of the T cells as a result of movement and growth, and as a result of interactions with the tumour cells, respectively.

Finally, tumour cells die (and are removed from their site) as a result of interactions with T cells in the same physical site at a rate $\tilde{\lambda} \geq 0$. The individual-based description of this is given by

$$\lambda(j, n_i^j) = \tilde{\lambda} j n_i^j.$$

Using these functions in a coarse-graining process similar to that in Sec. S1 and rescaling as appropriate, we find that the resulting system of equations is given by Eqs. (20) and (21), with continuum functions as described in Sec. 3.4.

S3 Numerical methods

The deterministic, continuum counterpart of the individual-based model described in Sec. 2 is given by the PDEs in Eqs. (5) and (12), with boundary conditions given in Eqs. (7)-(10) and initial conditions given in Eqs. (6) and (13).

To solve this system numerically, we use an advection-diffusion-reaction (A-DR) scheme that discretises the spatial variable x using a central finite difference stencil. In the phenotypic axis, y, we use a finite volume scheme, which divides the axis into $N_y + 1$ sites of equal width, controlled using the Koren limiter. The discretised equations which are solved numerically to produce the simulations take the following form:

$$\frac{\mathrm{d}n_i^j}{\mathrm{d}t} = \frac{((D^m)_{i+1}^j + (D^m)_i^j)(n_{i+1}^j - n_i^j) - ((D^m)_i^j + (D^m)_{i-1}^j)(n_i^j - n_{i-1}^j)}{2\Delta x^2} + \frac{n_i^{j+1} - 2n_i^j + n_i^{j-1}}{\Delta y^2}(D^p)_i^j + \mathcal{A}_{i,j} + n_i^j r(\bar{y}_j, N_i, e_i)$$

where

$$\begin{aligned} N_i &= \sum_j n_i^j \Delta y, \\ (D^m)_i^j &= D^m(\bar{y}_j, N_i, e_i), \\ (D^p)_i^j &= D^p(\bar{y}_j, N_i, e_i), \\ \bar{y}_j &= \text{mean of } y_j \text{ and } y_{j+1}, \\ \mathcal{A}_{i,j} &= \text{flux-limited advection in } y. \end{aligned}$$

The advection term in the y-direction is discretised using a slope-limited upwind scheme and can be written as:

$$\left(\frac{\partial}{\partial y}(v^p n)\right)_i^j \approx \frac{F_i^{j+1/2} - F_i^{j-1/2}}{\Delta y},$$

where $F_i^{j+1/2}$ is the numerical flux across the interface between phenotypic points y_j and y_{j+1}, given by:

$$F_i^{j+1/2} = \begin{cases} (v^p)_i^{j+1/2} n_i^{j,+} & \text{if } (v^p)_i^{j+1/2} > 0, \\ (v^p)_i^{j+1/2} n_i^{j+1,-} & \text{if } (v^p)_i^{j+1/2} \leq 0, \end{cases}$$

which is calculated using

$$\begin{aligned} R_i^j &= \frac{n_i^{j+1} - n_i^j}{n_i^j - n_i^{j-1}}, \\ n_i^{j,+} &= n_i^j + \frac{1}{2}\phi(R_i^j)(n_i^{j+1} - n_i^j), \\ n_i^{j+1,-} &= n_i^{j+1} - \frac{1}{2}\phi(1/R_i^j)(n_i^{j+1} - n_i^j). \end{aligned}$$

Alongside this, to simulate the evolution of the density of the local environment, as described by Eq. (12), we use the finite difference scheme, with a summation to approximate the integral, which can be written as

$$\frac{de_i}{dt} = -e_i \cdot \sum_j \nu(\bar{y}_j, n_i^j)\Delta_y.$$

This resulting system of ordinary differential equations are then integrated in time using python's in-built ordinary differential equation solver `scipy.integrate.solve_ivp` with the explicit Runge-Kutta integration method of order 5 and time step $\Delta_t = 0.1$. The phenotype step is $\Delta_y = 0.02$ and the spatial step is $\Delta_x = 0.1$, both of which were chosen to be sufficiently small to ensure that we observed convergence in the solutions.

S4 Supplementary figures

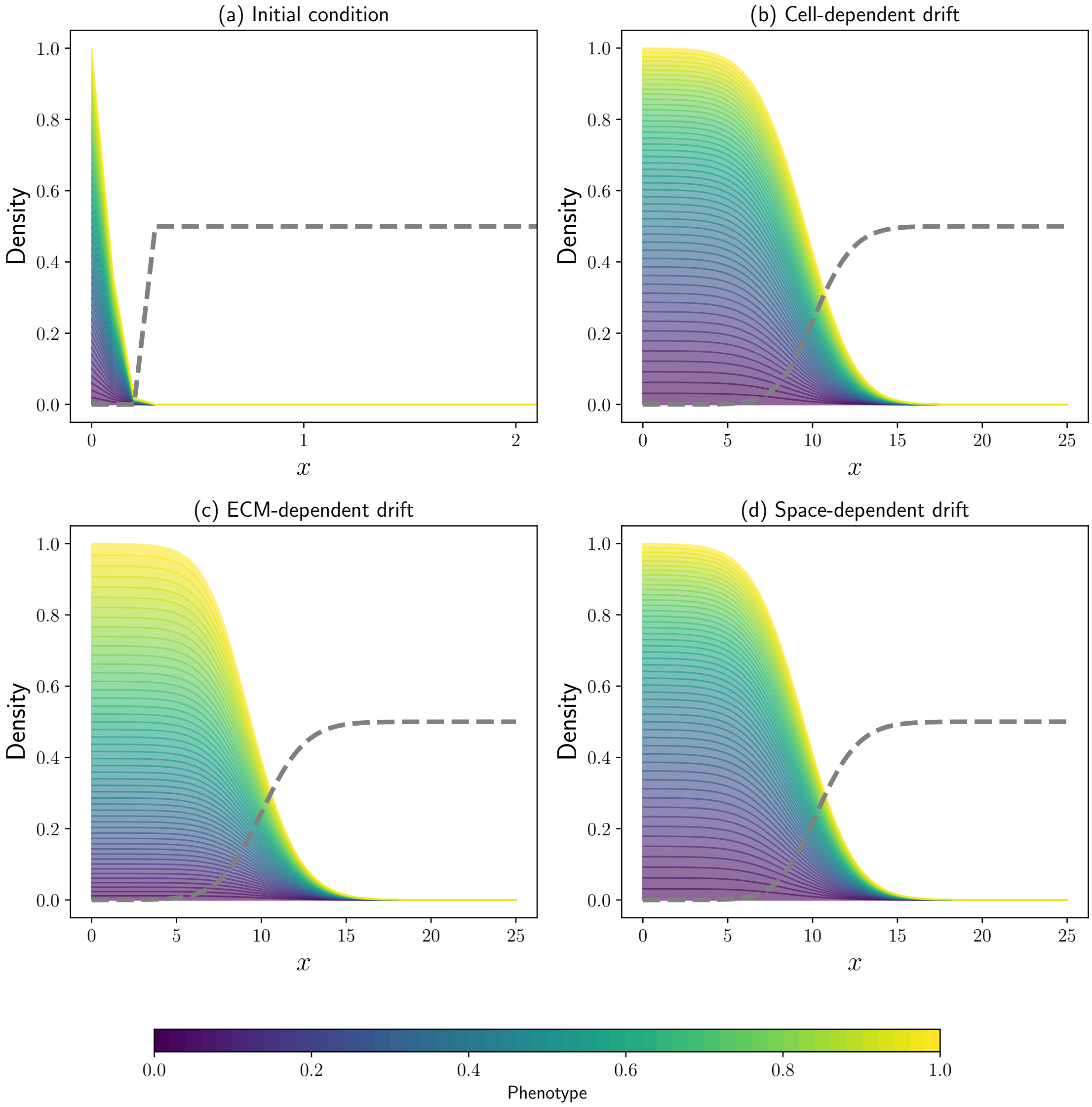

Figure S1: Evolution of the phenotypic structure of cells in Eqs. (5)–(12) subject to various phenotypic drift terms, with the corresponding ECM density shown as a dashed grey line. (a) The initial distribution of the ECM and the cells with different phenotypes. (b) The spatial structure of the invading wave subject to cell-dependent phenotypic drift. (c) The spatial structure of the invading wave subject to ECM-dependent phenotypic drift. (d) The spatial structure of the invading wave subject to space-dependent phenotypic drift. Results in (b), (c) and (d) are all plotted at time 30 and simulations are carried out with $\kappa = 1$. See Table 1 for explicit forms of the phenotypic drift terms.

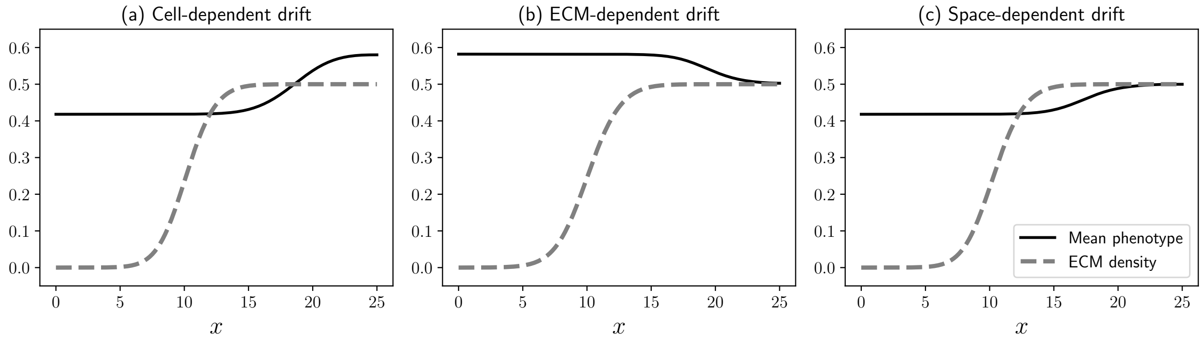

Figure S2: Evolution of the mean phenotype of the cells in Eqs. (5)–(12) subject to various phenotypic drift terms, with the corresponding ECM density shown as a dashed grey line. Results are all plotted at time $t = 30$ and simulations are carried out with $\kappa = 1$. See Table 1 for explicit forms of the phenotypic drift terms.
